# NKILA represses nasopharyngeal carcinoma carcinogenesis and metastasis by NF-κB pathway inhibition

**DOI:** 10.1371/journal.pgen.1008325

**Published:** 2019-08-20

**Authors:** Wei Zhang, Qiannan Guo, Guoying Liu, Fang Zheng, Jianing Chen, Di Huang, Linxiaoxiao Ding, Xing Yang, Erwei Song, Yanqun Xiang, Herui Yao

**Affiliations:** 1 Guangdong Provincial Key Laboratory of Malignant Tumor Epigenetics and Gene Regulation, Breast Tumor Center, Sun Yat-Sen Memorial Hospital, Sun Yat-Sen University, Guangzhou, People's Republic of China; 2 Department of Breast Surgery, The First Affiliated Hospital, Jinan University, Guangzhou, People's Republic of China; 3 Department of Nasopharyngeal Carcinoma, Sun Yat-sen University Cancer Center, Sun Yat-Sen University, Guangzhou, People's Republic of China; 4 Medical Research Center, Sun Yat-Sen Memorial Hospital, Sun Yat-Sen University, Guangzhou, People's Republic of China; 5 Department of Oncology, Sun Yat-Sen Memorial Hospital, Sun Yat-Sen University, Guangzhou, People's Republic of China; Novartis, UNITED STATES

## Abstract

The role of long non-coding RNA (lncRNA) in the progression of Nasopharyngeal carcinoma (NPC) has not been fully elucidated. The study was designed to explore the functional role of NKILA, a newly identified lncRNA, in the progression of NPC. We performed a lncRNA expression profile microarray using four NPC and paired para-cancerous tissues. NKILA was identified as a potential functional lncRNA by this lncRNA expression profile. We used 107 paraffin-embedded NPC tissues with different TNM stages to detect the expression of NKILA and analyzed the survival data by Log-rank test and Cox regression. The role of NKILA and its underlying mechanisms in the progression of NPC were evaluated by a series of experiments *in vitro and vivo* by silencing or expressing NKILA. Compared with control tissues, NKILA expression was identified to be decreased in NPC tissues. Low NKILA expression was correlated with unfavorable clinicopathological features and predicted poor survival outcome in NPC patients. After adjusting for potential confounders, low expression of NKILA was confirmed to be an independent prognostic factor correlated with poor survival outcomes. Furthermore, we found that NKILA overexpression in high-metastatic-potential NPC cells repressed motile behavior and impaired the metastatic capacity *in vitro* and *in vivo*. In contrast, RNAi-mediated NKILA depletion increased the invasive motility of cells with lower metastatic potential. Further experiments demonstrated that NKILA regulated the metastasis of NPC through the NF-κB pathway. Taken together, NKILA plays vital roles in the pathogenesis of NPC. The unique histological characteristics of NPC indicate that local inflammation plays a vital role in carcinogenesis of nasopharyngeal carcinoma.

## Author summary

NF-κB is a pivotal link between NPC and inflammation. Importantly, NF-κB was found to be overexpressed in nearly all NPC tissues, and inflammatory cytokines have also been observed in NPC tissues. Inflammatory cytokines promote the susceptibility of NPC cells to metastasize via constant NF-κB activation. Here, we found that NKILA, a newly identified lncRNA, is upregulated by inflammatory cytokines and is significantly downregulated in NPC. By a series of *in vitro and in vivo* experiments, we show that NKILA exerts its effect as a tumor suppressor via inhibiting tumorigenesis and metastasis of NPC. Further studies indicate that NKILA regulates the metastasis of NPC through NF-κB pathway. Our research demonstrates that NKILA plays a critical role in the progression of NPC. These findings are particularly important as they provide new insights into the effects of inflammation on the biology of NPC. NKILA might be a candidate molecular marker and a novel therapy target for NPC patients.

## Introduction

Nasopharyngeal carcinoma (NPC), a metastasis-prone cancer, which is particularly common in southeast Asia and southern China [1–4]. Due to the high radiosensitivity, radiotherapy has become the main treatment for locoregional NPC. Radiation oncology has improved the locoregional control(the tumor control of nasopharynx and neck lymph nodes), the development of distant metastasis becomes the major reason for treatment failure and occurs in 30–40% of patients with locoregional advanced NPC [5]. Thus, the assessment of the metastatic potential of NPC is vital for determining prognosis and treatment.

Long non-coding RNAs play pivotal regulatory roles in the physiological and pathological processes. Most lncRNAs regulate gene expression by RNA decay control, chromatin remodeling, and enhancer transcription in cis and epigenetic regulation [6–10]. Several lncRNAs are aberrantly expressed or play important roles in NPC, such as HOTAIR, ENST00000438550, and AFAP1-AS1 [11–15]. Inflammatory cytokines have been observed in NPC tissues and can promote the susceptibility to metastasis of NPC cells via constant NF-κB activation [16–18], therefore NF-κB is a pivotal link between NPC and inflammation. Interestingly, NF-κB is found to be overexpressed in nearly all NPC tissues [16, 19, 20]. NKILA is an NF-κB-interacting lncRNA [21], our previously study found it can be upregulated by inflammatory cytokines in breast cancer. By interacting with NF-κB/IκB, NKILA forms a stable complex, subsequently it masks the IκB phosphorylation motifs to repress the phosphorylation of IκB induced by IKK, then repress NF-κB pathway activation. But the role of NKILA in nasopharyngeal carcinoma remains unknown.

In our study, we examined NKILA expression in normal nasopharyngeal tissue, NPC tissue and cell lines. We proved that low NKILA expression predicts poor patient prognosis and that NKILA regulates the metastasis of NPC by the NF-κB pathway. In addition, we explored the role of NKILA in NPC carcinogenesis and metastasis.

## Results

### NKILA is significantly downregulated in nasopharyngeal carcinoma

**Fig 1:**
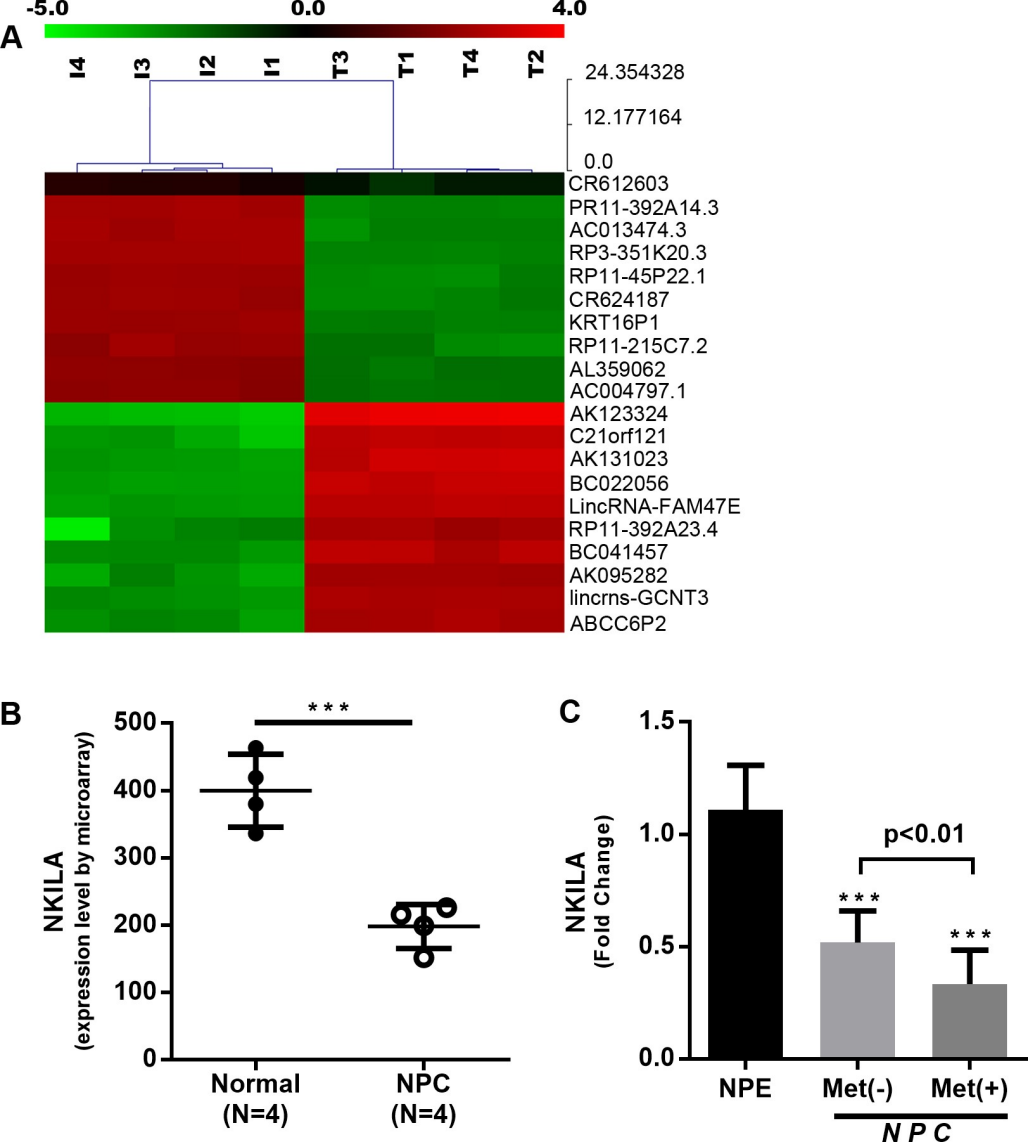
NKILA is downregulated in nasopharyngeal carcinoma. **(A)** Hierarchical clustering demonstrating distinguishable lncRNA expression profiles among samples from microarray data (P < 0.05). **(B)** NKILA expression in nasopharyngeal carcinoma as observed by microarray (mean ± SD, ***, P < 0.001 versus Normal). **(C)** Quantification of NKILA expression by qRT-PCR using NPC fresh frozen tissues. 10 cases of NPE,18 cases of NPC Met (-), 8 cases of NPC Met (+). (***, P < 0.001 versus NPE).

To evaluate the role of lncRNA in the progression of NPC, we performed lncRNA expression profiles using four paired NPC and para carcinoma tissues by microarray. We observed that 2107 lncRNAs were upregulated while 2090 lncRNAs were downregulated by more than 2-fold, NKILA among these downregulated lncRNAs (Fig 1A, GSE95166). Quantitative RT-PCR verified the significant reduction in the expression of NKILA in NPC (Fig 1B). To confirm the results, NKILA expression levels were detected in fresh frozen tissues (26 NPC and 10 control tissues) by qRT-PCR. Compared with control tissues, NKILA was significantly downregulated in NPC tissues (Fig 1C). Furthermore, patients with developed distant metastasis have a lower NKILA expression than patients with non-metastatic NPC (P < 0.05, Fig 1C, Table 1). The results imply that low expression level of NKILA is correlated with the progression of NPC.

**Table 1:** Correlation between clinicopathological features and NKILA expression.

characteristics		NKILA in situ hybridization (n = 107)		
		L	H	P
**Age (years)**	≤50	35	23	.74
	>50	28	21	
**Gender**	Female	12	10	.64
	Male	51	34	
**Clinical stage**	I-II	20	27	.00
	III-IV	43	17	
**T stage**	T1-2	32	33	.01
	T3-4	31	11	
**N stage**	N0-1	45	36	.22
	N2-3	18	8	
**Metastasis**	No	42	38	.02
	Yes	21	6	

Abbreviations: L, Low level; H, High level; P, P value.

### Association between NKILA expression and clinicopathological features in nasopharyngeal carcinoma

**Fig 2:**
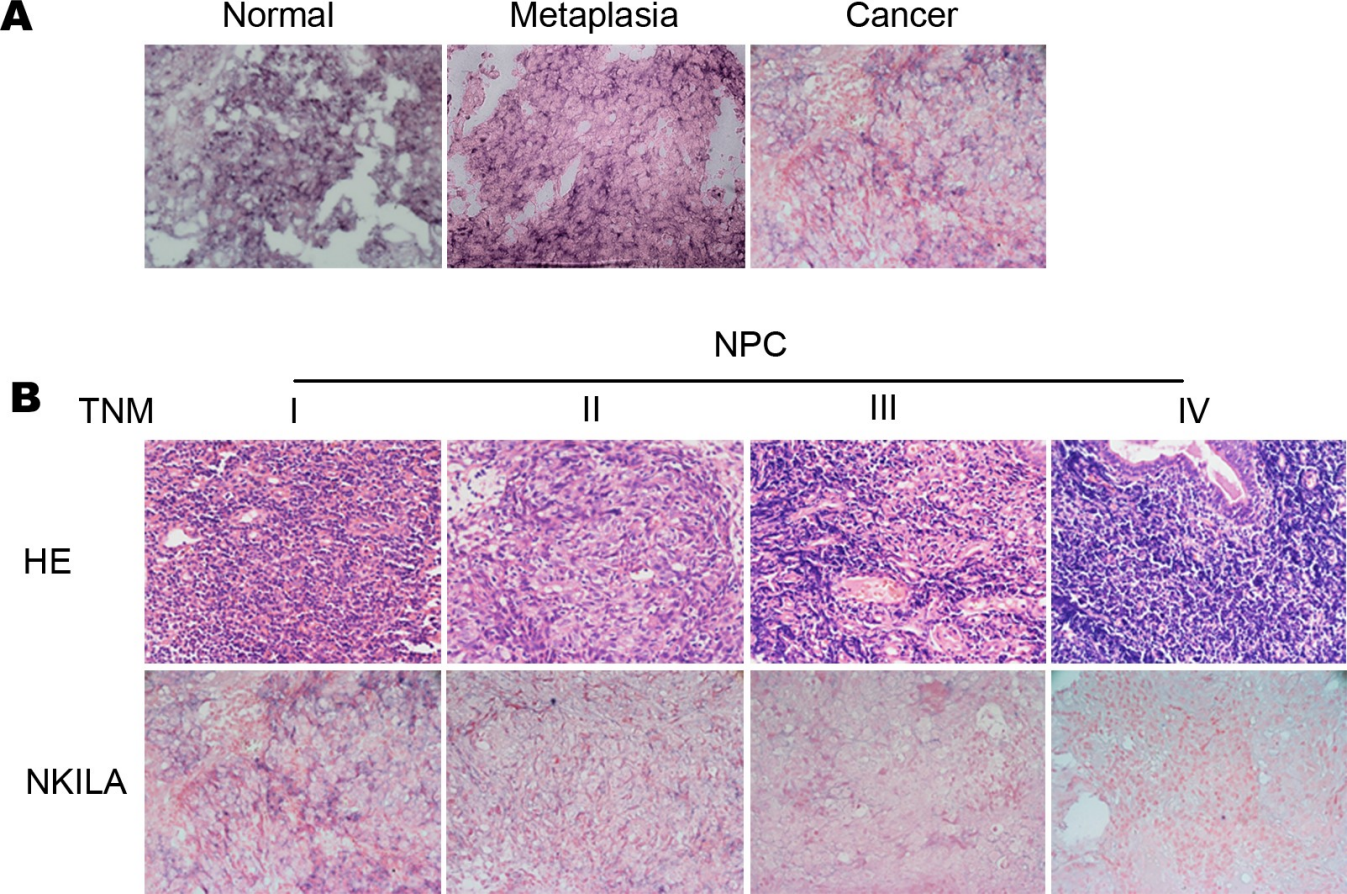
Low expression of NKILA is correlated with NPC progression. Representative images (×400) **(A)** ISH for NKILA were performed using normal nasopharyngeal tissue, metaplasia nasopharyngeal tissue with atypical hyperplasia and NPC tissue. **(B)** H&E staining and ISH for NKILA were performed using paraffin-embedded NPC tissue with stage I to IV.

We further detected the expression of NKILA in 107 paraffin-embedded NPC tissues to evaluate the clinical significance of NKILA in patients with NPC. Scattered NKILA staining was observed in NPC cells cytoplasm, and the mean optical density (MOD) was used to quantify the NKILA staining. NKILA expression levels were compared in normal nasopharyngeal epithelia and samples from different stages of NPC. As shown in Fig 2A, NKILA was found abundantly expressed in normal nasopharyngeal epithelia and nasopharyngeal cells of metaplasia with atypical hyperplasia (Fig 2A) (P < 0.01), with a significantly higher MOD of NKILA staining compared with NPC tissues. Furthermore, NKILA staining decreased significantly with advanced disease staging in TNM stage I to III NPC, and staining was almost absence in stage IV tumors (Fig 2B).

NKILA expression was associated with the clinicopathological features of NPC patients (Table 1). Low expression of NKILA was correlated with metastasis (P < 0.05), larger tumor size (P <0.05), and late clinical stage of NPC patients (P < 0.005, Table 1). Other parameters (i.e. age, gender and lymph node status) were not found direct association with NKILA expression (P > 0.05).

### Low NKILA expression predicts poor prognosis of NPC patients.

**Fig 3:**
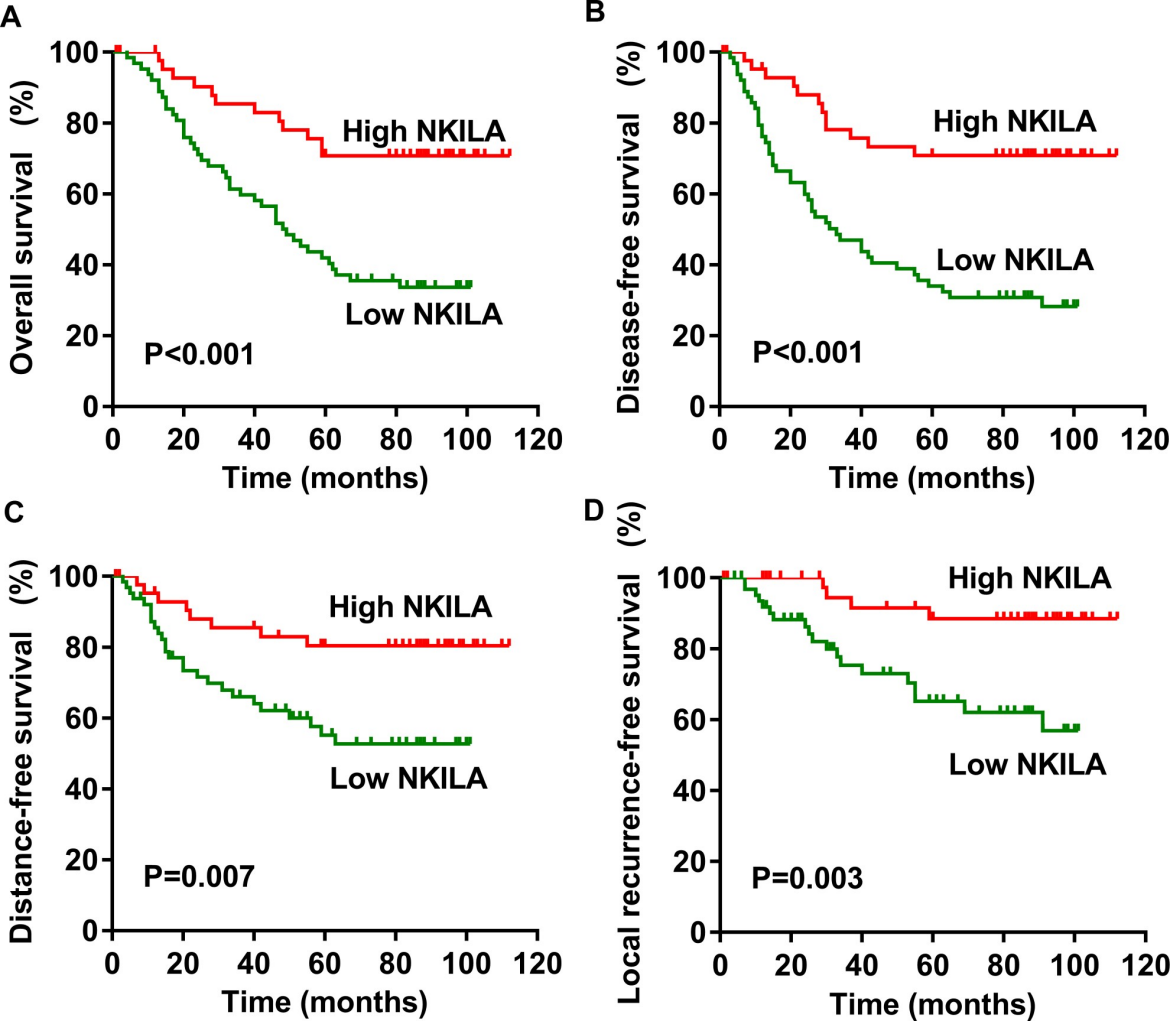
Low NKILA expression is correlated with poor survival in patients with NPC. Kaplan–Meier survival curve for NPC patients with low NKILA expression (SI ≤ 2) or high NKILA expression (SI > 2), median follow-up period is 83 months. **(A)** Overall survival. **(B)** Disease-free survival. **(C)** Distant metastasis-free survival. **(D)** Local recurrence-free survival.

This study suggested that NPC patients with high expression of NKILA had a significantly longer survival, the median follow-up time is 83 months (Fig 3A, P < 0.001). Furthermore, high expression of NKILA predicts a longer disease-free survival (DFS) (Fig 3B, P < 0.001), distant metastasis-free survival (DMFS) (Fig 3C, P <0.01), and local recurrence-free survival (LRFS) (Fig 3D, P <0.01). After adjusting for potential confounders, the multivariate analysis showed that high expression of NKILA was significantly correlated with improved OS (HR, 0.327; 95% CI, 0.171–0.623; P < 0.001), DFS (HR, 0.290; 95% CI, 0.153–0.549; P < 0.001), DMFS (HR, 0.353; 95% CI, 0.159–0.781; P = 0.010), and LRFS (HR, 0.227; 95% CI, 0.077–0.670; P < 0.01, Table 2).

**Table 2:** Multivariable analysis for the 107 cohorts.

Variable	Cox Multivariable Analysis for OS			Cox Multivariable Analysis for DFS			Cox Multivariable Analysis for DMFS			Cox Multivariable Analysis for LRFS		
	*HR*	*95% Cl*	*P*	*HR*	*95% Cl*	*P*	*HR*	*95% Cl*	*P*	*HR*	*95% Cl*	*P*
**Gender**												
**Female**	reference			reference			reference			reference		
**Male**	1.102	0.534–2.273	0.793	1.280	0.622–2.632	0.503	1.804	0.669–4.864	0.244	0.807	0.278–2.347	0.694
**Age (years)**												
**≤50**	reference			reference			reference			reference		
**>50**	1.405	0.821–2.402	0.215	1.284	0.760–2.169	0.351	1.849	0.939–3.641	0.076	0.820	0.359–1.873	0.638
**T stage**												
**T1–T2**	reference			reference			reference			reference		
**T3–T4**	1.081	0.440–2.655	0.865	0.968	0.403–2.328	0.943	0.891	0.302–2.628	0.835	1.142	0.264–4.946	0.859
**N stage**												
**N0–N1**	reference			reference			reference			reference		
**N2–N3**	1.172	0.464–2.948	0.737	1.027	0.415–2.545	0.954	1.249	0.404–3.860	0.699	0.883	0.207–3.766	0.866
**Clinical stage**												
**I–II**	reference			reference			reference			reference		
**III–IV**	1.219	0.438–3.395	0.704	1.201	0.443–3.258	0.718	1.322	0.382–4.574	0.659	1.009	0.196–5.181	0.992
**chemotherapy**												
**No**	reference			reference			reference			reference		
**Yes**	0.536	0.277–1.037	0.064	0.570	0.307–1.058	0.075	0.586	0.262–1.308	0.192	0.686	0.270–1.741	0.427
**NKILA expression**												
**Low**	reference			reference			reference			reference		
**High**	0.327	0.171–0.623	0.001	0.290	0.153–0.549	0.001	0.353	0.159–0.781	0.010	0.227	0.077–0.670	0.007

Abbreviations: HR, hazard ratio; 95%CI, 95% confidence interval; OS, overall survival; DFS, disease-free survival; DMFS, distant metastasis-free survival; LRFS, local recurrence-free survival

### The tumorigenesis of NPC cells is repressed by the expression of NKILA

**Fig 4:**
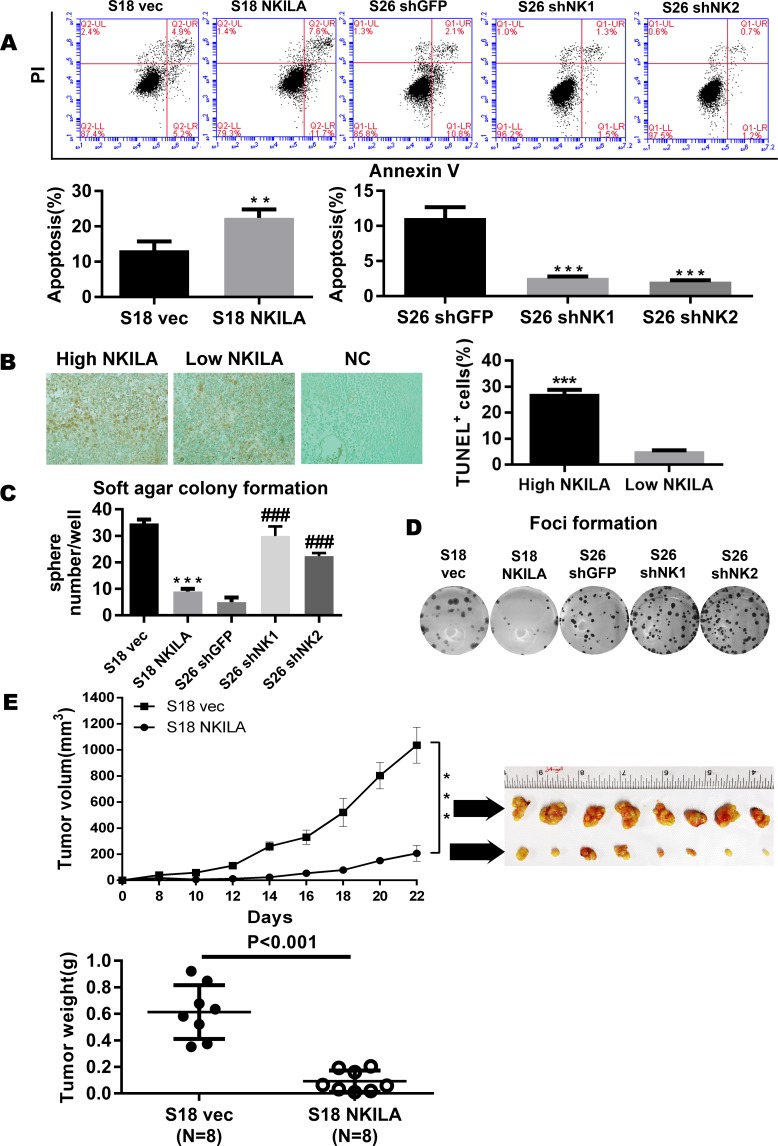
NKILA represses the tumorigenesis of NPC. **(A)** Apoptosis of control vector or NKILA-overexpressing S18 cells and shGFP or shNKILA S26 cells as assayed by ANNEXIN-V/PI staining(mean±SD, n = 3,***<0.001 versus S26 shGFP,**<0.01versus S18 vec). **(B)** Tunnel assay revealing the correlation between NKILA and apoptosis in 65 paraffin-embedded NPC specimens. **(C)** Anchorage-independent growth of control vector or NKILA-overexpressing S18 cells and shGFP or shNKILA S26 cells in soft agar. Over 14 days of culture, number of colonies was calculated at 10 randomly ten fields of view. Original magnification, ×100. (Error bar represent SD. Mean ± SD, n = 3, ***, P < 0.001 versus S18 vec; ###, P < 0.001 versus S26 shGFP). **(D)** Foci formation by control vector or NKILA-overexpressing S18 cells and shGFP or shNKILA S26 cells were performed as described in the Methods. **(E)** Tumor formation in nude mice: S18 (vec, NKILA) cells were injected and tumor volumes were calculated and plotted as described in the Methods. The upper panel shows the tumor growth and tumor volume in mice (S18 vector control, S18 NKILA overexpressing cells). The tumor weight is also shown in the lower panel, and tumors treated with vec are much heavier, indicating that NKILA can inhibit tumor growth.

NKILA suppressed the activation of NF-κB, which in turn inhibited tumorigenesis induced by aberrant NF-κB signaling by modulating apoptosis and invasion [21–23]. We observed that enforcing NKILA expression increased apoptosis in S18 cells (S18 vec vs S18 NKILA: 10.1% vs 19.3%, p<0.01). Conversely, silencing NKILA in S26 cells reduced apoptosis (Fig 4A, P < 0.05), suggesting that NKILA modulates apoptosis in NPC cells. We next verified the association of the NKILA expression level with apoptosis. We examined the apoptosis level in clinical NPC tissues using a TUNEL assay and the expression level of NKILA using *in situ* hybridization. The expression level of NKILA was positively associated with the tumor apoptosis level (Fig 4B, P < 0.001). Next, the regulation of NKILA in the tumorigenic activity during anchorage-independent growth in NPC cells was evaluated. As shown in Fig 4C and 4D, overexpression NKILA induced significant inhibition of anchorage-independent growth in S18 cells, as revealed by a decrease in the number and size of colonies (P < 0.001). Conversely, the depletion of endogenous NKILA in S26 cells induced a significant increase in the number and size of colonies (P < 0.001).

In addition, to explore the role of NKILA in tumorigenesis *in vivo*, xenograft tumor experiments were performed. S18 cells overexpressing NKILA or carrying a control vector were injected subcutaneously into the flank of nude mice, and then we measured the tumor size every 2 days to calculate the tumor volume. As shown in Fig 4E, NKILA overexpression in S18 cells greatly inhibited the tumor growth (P < 0.001), demonstrating that downregulation of NKILA is required for the malignant transformation of nasopharyngeal epithelial cells.

### Upregulation of NKILA represses the metastatic potential of cells in NPC

**Fig 5:**
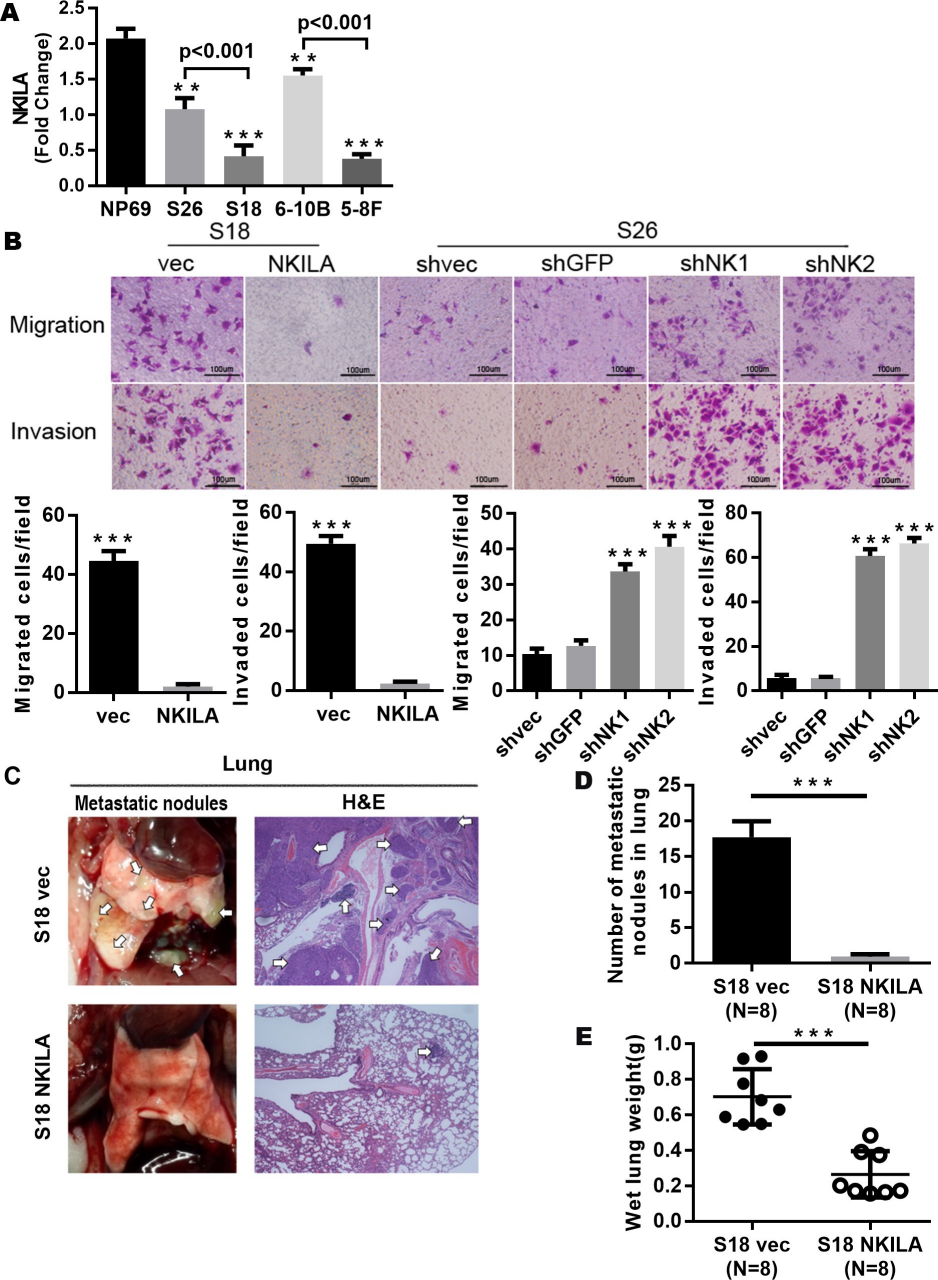
NKILA repress the metastasis of NPC by inhibiting the NF-kB pathway. **(A)** NKILA expression in NPC cell lines determined by qRT-PCR assay (Mean ± SD, n = 3, ***, P < 0.001, **, P < 0.01versus NP69). **(B)** Representative images of migrated and invaded NPC cells with overexpressed or silenced NKILA in Boyden chamber assay (Mean ± SD, n = 3, ***, P < 0.001, versus control). **(C)** Representative images of the lung metastasis, metastatic nodes were indicated by Arrowheads. Left: Representative lungs, Right: Representative image (×200) of lung with metastasis by H&E staining. **(D)** 6 weeks after tail vein injection, number of metastases in lungs of each mice (mean±SD) were calculated. **(E)** The wet lung weight of the mice treated with the control vehicle was significantly higher, suggesting that mouse in control group had more or greater metastases, indicating that NKILA inhibited tumor metastasis.

Next, we detected NKILA expression in NPC cell line to further explore the regulatory function of NKILA in NPC. Two paired NPC cell lines with high metastatic potential (S18 and 5-8F) and low metastatic potential (S26 and 6-10B) were used in the experiment. We found that NKILA expression levels increased by 2.6-fold in S26 (P < 0.001, versus S18) and by 4.1-fold in 6–10B (P < 0.001, versus 5-8F) (Fig 5A). It was shown that NPC cell lines with low metastatic potential cells had a higher NKILA expression level.

To explore the effect of NKILA on metastatic potential of NPC cells, we overexpressed NKILA in S18 NPC cells and examined the resulting metastatic potential. As shown in Fig 5B, S18 NPC cells overexpressing NKILA exhibited reduced migration and invasiveness (Fig 5B). Conversely, silencing NKILA significantly promoted the mobility of S26 cells, the metastatic potential of cells was enhanced.

Experimental metastasis assay was performed to evaluate the effect of NKILA on metastasis *in vivo*. We injected S18 cells with enforced overexpression of control vector or NKILA into the lateral tail vein of nude mice (4 weeks old), metastatic nodules in lungs were evaluated, numbers of metastatic nodules in lungs were markedly decreased in mice injected with S18 cells overexpressing NKILA, as shown in Fig 5C–5E. The number and volume of micrometastases in lungs of mice injected with S18 cells overexpressing NKILA were proven significantly reduced by H&E staining (Fig 5C). The results suggest that NKILA is extremely important for the metastatic development of S18 cells.

### NKILA represses the NF-κB pathway activation via inhibiting IκBα phosphorylation

**Fig 6:**
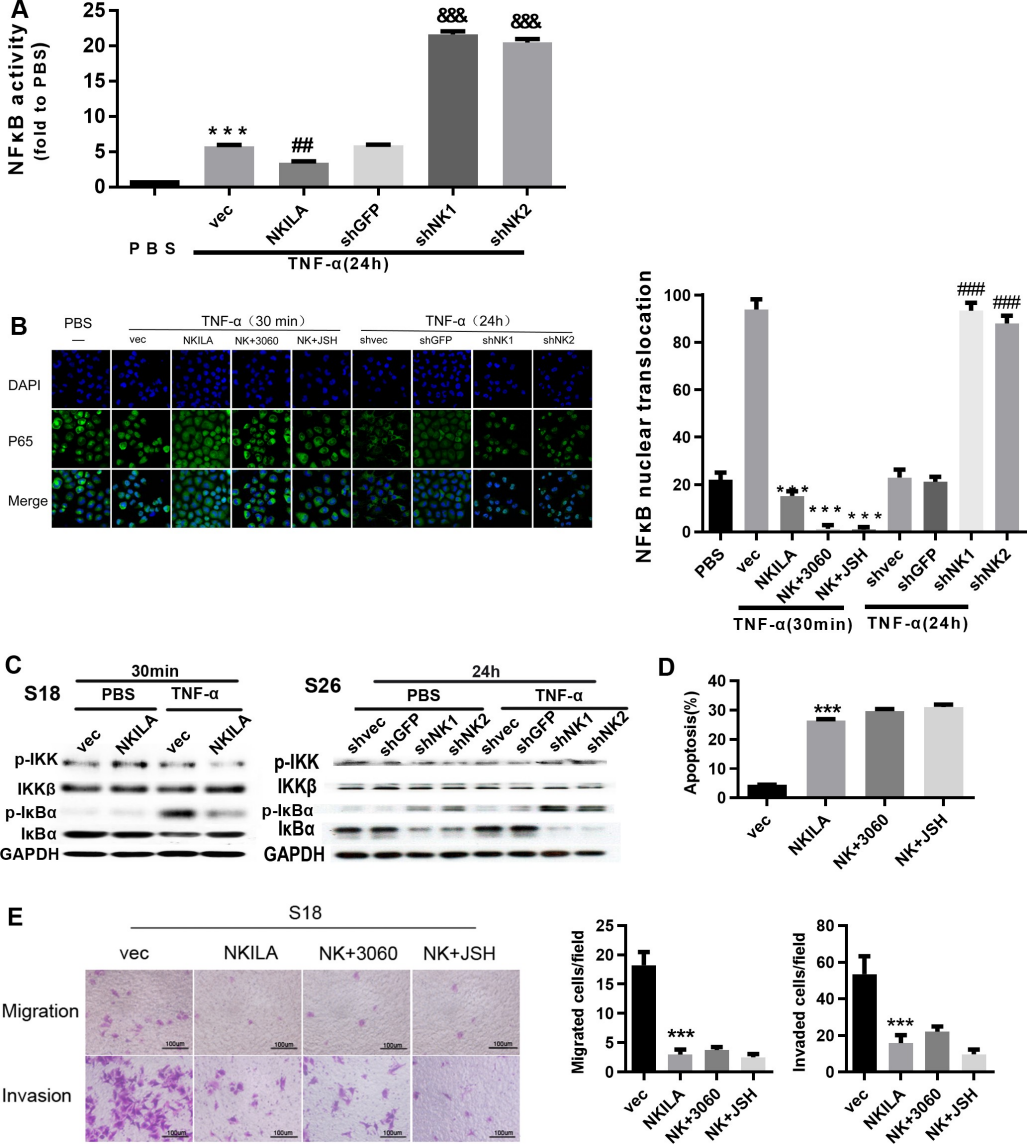
NKILA inhibits the activation of NF-κB by repressing IκB phosphorylation. **(A)** Luciferase reporter assay for detection of NF-κB activity in S26 cells treated with TNF-α (mean ± SD, n = 3, ^**&&&**^, P < 0.001 versus shGFP; ^**##**^, P < 0.01 versus vec; ***, P < 0.001 versus PBS). **(B)** Immunofluorescence confocal microscopy for detection of P65 nuclear translocation in S26 cells with overexpression or depletion of NKILA treated with TNF-α(mean ± SD, n = 3,***, p < 0.001 versus vec, ###, P < 0.001 versus shvec).**(C)** Total and phosphorylated IKK and IκBα assayed by Western blotting in S26 or S18 cells. **(D)** Annexin-V/PI staining for detection of apoptosis in S18 cells stably expressing NKILA (48 h after seeding). (mean ± SD, n = 3, ***, P < 0.001 versus vec; 3060:10 mM; JSH:5 mM). **(E)** Migration and invasion in S18 cells with stably overexpression or depletion of NKILA, assayed by Boyden Chamber assay (16h for migration and 22 h for invasion after seeding, respectively) (mean ± SD, n = 3, ***, P < 0.001 versus S18 vec).

NKILA is up-regulated by inflammatory cytokines, which resulted in the inhibition of IKK -induced IκB phosphorylation and then repressed NF-κB pathway activation [21]. Nuclear translocation of P65 is emerging as a central feature of NF-κB pathway activation. Moreover, NKILA binding to the NF-κB: IκBα complex is essential for inhibition of NF-κB activation [21]. Thus, we evaluated the NF-κB activation by detecting NF-κB transcription activity and P65 translocation in NPC cells stimulated by inflammatory cytokines. We found that NKILA suppressed the enhancement of NF-κB transcriptional activity by TNFα. Conversely, silencing NKILA increased NF-κB transcriptional activity by more than 3-fold (Fig 6A). The enhancement of NF-κB transcriptional activity in S26 was suppressed by 40%, but further increased by 3.5-fold after NKILA was silenced. In addition, upregulation of the expression of NKILA resulted in retention of most of the P65 in the cytoplasm upon TNF-α stimulation, whereas nearly all of the P65 translocated to the nucleus in cells carrying empty vector (Fig 6B and S2 Fig). Conversely, silencing of endogenous NKILA expression led to prolonged P65 translocation to the nucleus upon TNF-α stimulation in S26 cells. Our results suggest that NKILA inhibits the activation of NF-κB pathway in S26 cells.

**Fig 7:**
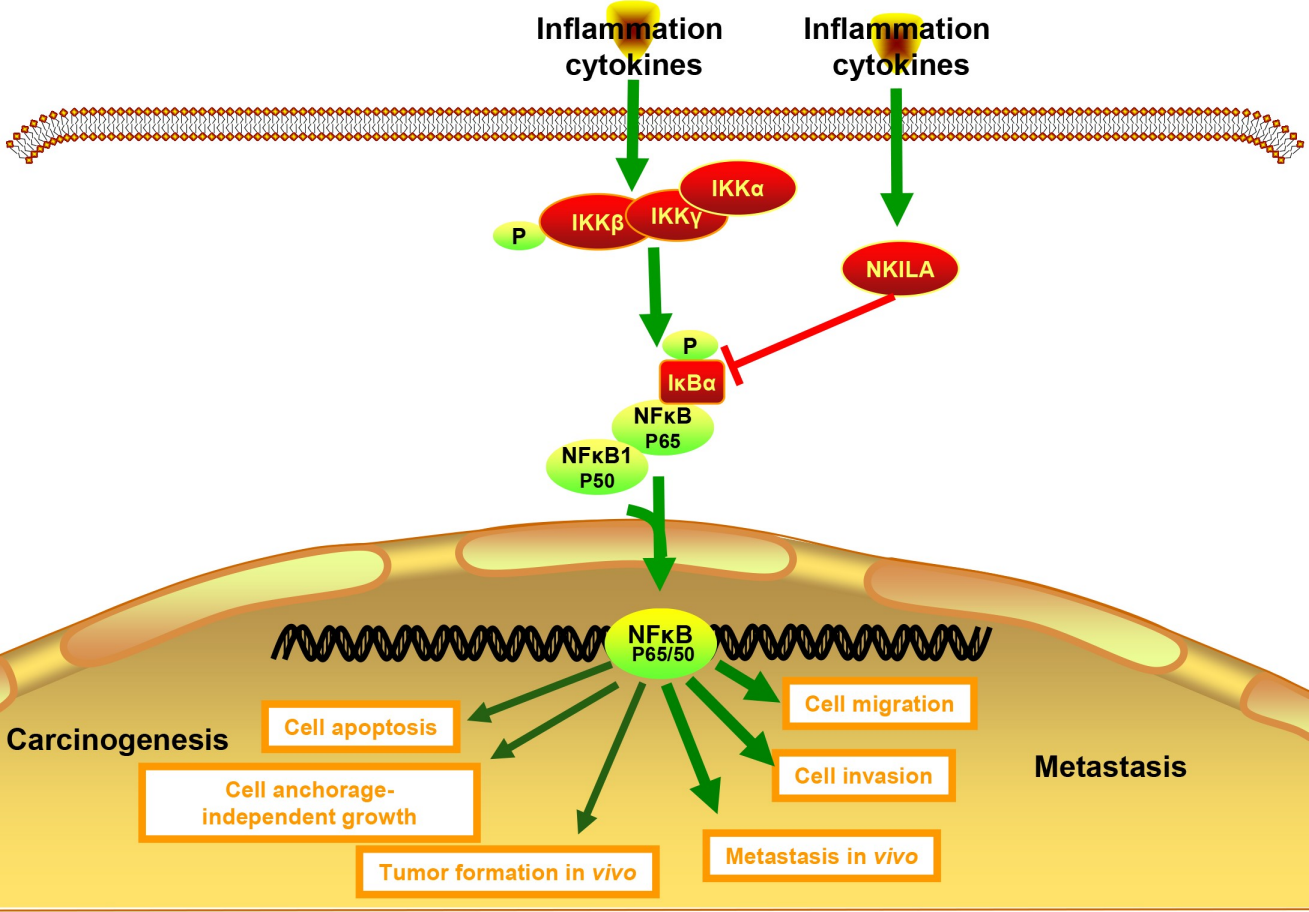
NKILA inhibits the NPC carcinogenesis and metastasis by NF-κB pathway inhibition. Schematic figure depicting the role and mechanism of NKILA in NPC carcinogenesis and metastasis.

Subsequently, we evaluated the role of NKILA on IκBα and IKK phosphorylation and explored the mechanisms by which NKILA inhibits NF-κB activation in NPC. It revealed that both basal phosphorylation and TNF-α-induced phosphorylation of IκBα were repressed by enforced expression of NKILA in S18 cells; additionally, silencing NKILA increased the phosphorylation of IκBα with or without the stimulation of TNF-α in S26 cells (Fig 6C and S1 Fig). However, the phosphorylation of IKK was not influenced by NKILA expression (Fig 6C). Our study indicates that NKILA inhibits the activation of NF-κB primarily by inhibiting the phosphorylation of IκBα but not IKK in S18 and S26 cells. To confirm that NKILA works by inhibiting NF-κB, we used sc-3060 and JSH-23 to abrogate the nuclear translocation of NF-κB. As shown in Fig 6D and 6E, NKILA did not further increase the apoptosis of S18 cells, nor did it reduce the migration and invasion of S18 cells, suggesting that role of NKILA in NPC cell lines is dependent on IκBα.Collectively, our data show that NKILA represses the progression of NPC by inhibition of NF-κB pathway (Fig 7).

## Discussion

NPC, a malignant tumor with high tendency for metastasis, is very common in southern China. Patients of NPC presented with distant metastasis at diagnosis accounts for 5–8% of all cases; furthermore, after standard treatment, the proportion of distant metastasis in stage III–IV NPC is still as high as 30% [5, 24]. This tendency for metastasis emphasizes the urgency of elucidating the molecular mechanism underlying tumorigenesis and metastasis and possibly to develop new treatment for NPC. In our study, we first discovered that a long non-coding RNA named NKILA which was down-regulated in nasopharyngeal carcinoma, and we have demonstrated that overexpression of NKILA repressed the motile behavior and metastatic capacity of NPC cells. As previously reported, NKILA represses NF-κB activation by directly or indirectly inhibiting phosphorylation of IκBα in breast and hepatocellular carcinoma[21, 25]. We further demonstrated that NKILA could repress the metastasis of NPC by inhibiting NF-κB pathway. Furthermore, we identified that decreased NKILA expression was correlated with unfavorable clinicopathological features and poor survival outcomes in NPC patients.

The roles of lncRNAs (eg: HOTAIR, MALAT1, HOTTIP) have been confirmed by functional studies [26–30]. Dysregulated lncRNAs have been observed in NPC, and clusters of lncRNAs are dysregulated during the metastasis cascade [11, 12]. Nevertheless, the expression profiles of lncRNA in paired NPC and para carcinoma tissues have never been reported, and most of the dysregulated lncRNAs in NPC are largely unknown. Human lncRNA microarray was firstly performed in our study to detect the expression profiles in paired NPC and para carcinoma tissues and found that 4197 lncRNAs were dysregulated by more than 2-fold. Among these dysregulated RNAs, NKILA was found significantly downregulated in NPC, the decline of NKILA was more pronounced in patients with distant metastases, consistent with previously reported in breast cancer, Hepatocellular carcinoma and laryngeal cancer [21, 25, 31–33]. Second, we showed that NKILA expression decreased significantly with advanced disease staging in NPC clinical tissue samples, further research showed low expression of NKILA was correlated with metastasis (P< 0.05), larger tumor size (T stage, P <0.05), and late clinical stage (TNM stage, P < 0.01). No association was observed between NKILA expression and lymph node states (N stage) in NPC, but NKILA expression was significantly correlated with the lymph node in breast cancer (P < 0.001) [21]. As previously reported in other type of cancers, our study indicated that NPC patients with high NKILA expression survived significantly longer (OS, P < 0.001) or longer DFS (P < 0.001) [21, 31, 32]. We further provided evidence that patients with high expression of NKILA had longer DMFS and LRFS (P = 0.01, P <0.01 respectively), which was clinically significant for patients with NPC. Our study is the first to demonstrate that low NKILA expression predicts poor prognosis of NPC patients, with reinforcement data from multivariate analysis (Table 2). Additionally, aberrant activation of NF-κB may promote chronic inflammation even tumorigenesis in certain conditions which is correlated with NPC progression [18, 34–36]. Here, we demonstrate that overexpression of NKILA promotes apoptosis and represses the invasion of NPC cell lines as reported in breast cancer cells. NKILA can also enhance the effect of baicalein on cell apoptosis and metastasis in HCC [21, 25]. Furthermore, NKILA is firstly proved to be positively associated with apoptosis in human NPC tissues. The present study further confirms that NKILA represses tumorigenic and metastatic ability of NPC cells.

NKILA is firstly identified as upregulated by inflammatory cytokines through NF-κB pathway in breast cancer, in return NKILA regulate the metastasis of breast cancer via NF-κB pathway [21]. Whether NKILA regulates tumorigenesis and metastasis in NPC via NF-κB and its mechanism remains unclear. In the present study, we verified that NKILA suppressed the enhancement of NF-κB transcriptional activity by TNFα. In addition, upregulation of the expression of NKILA resulted in retention of most of the P65 in the cytoplasm upon TNF-α stimulation, In contrast, the depletion of NKILA expression significantly prolonged the sustained activation time of NF-κB pathway, our study firstly demonstrated that NKILA can regulate the NF-κB activation in NPC. The results are consistent with our previous study in breast cancer [21]. P65 was also found upregulated in laryngeal cancer tissues. P65 positively regulates the NKILA expression, however, NKILA inhibits the translocation of P65 to reduce the resistance of cells to X-ray radiation in laryngeal cancer [33]. Subsequently, we showed that NKILA mainly inhibits the phosphorylation of IκBα, rather than activating IKK to repress NF-κB activation in NPC cells. In addition, we used sc-3060 and JSH-23 to abrogate P65 nuclear translocation, and no further increased apoptosis or reduced migration and invasion was observed in NKILA overexpressing NPC cells. Our previous study shows that NKILA has a high affinity for P65 [21]. By binding to P65, NKILA forms a complex with the IKB/NF-κB complex, and then masks the IKK phosphorylation site to inhibit IκB phosphorylation. In the present study, it is confirmed that NKILA exerts its effect on NPC by inhibiting NF-κB activation. The important effect of NF-κB pathway in NPC progression is well known, but most studies focus on the activation of P50/P65 in NPC cells[37–40].It has been reported that FN1 regulates apoptosis of NPC cells though P65 in the NF-κB pathway [41]. Epstein-Barr virus (EBV) expresses high levels of BamHI-A rightward transcripts (BARTs) in NPC.LMP1 binds P50 to NF-κB sites in the promoter of BART and activates the BART promoters via NF-κB pathway, an autoregulatory loop is formed in NPC cells to maintain EBV latency [42]. The upstream component of NF‐κB or the targeted gene of NF-κB has also been reported to contribute to aberrant NF-κB pathway activation [37, 43, 44], MiR-125b has been shown to regulate NF-κB pathway activity by targeting A20 in NPC cells [45]. To our knowledge, no studies have demonstrated that NF-κB activation associated with NPC progression is primarily regulated by inhibition of IκBα phosphorylation in NPC, and more importantly, in the current study, we extended the understanding of the autoregulatory loop of inflammatory factors and NF-κB activation. Consistent with the important regulatory role of NKILA in breast cancer [21, 46], we demonstrate that NKILA regulates the progression of NPC cells by regulating NF-κB pathway activity.

Nearly all NPCs are EBV-associated tumor. EBV-encoded LMP1, a transmembrane protein, has been identified as a viral oncogene of NPC [47]. LMP1 induces constitutive activation of NF-κB by transforming effector sites 1 and 2, which is required for efficient B-lymphocyte transformation. NF-κB activation maintains the survival of EBV-transformed lymphoblastoid cells, and blocking NF-κB signal leads to the death of these malignant cells [48]. Interestingly, we demonstrate for the first time that NKILA, a lncRNA that is upregulated by inflammatory cytokines, inhibits NF-κB activation by repressing IκB phosphorylation induced by IKK in NPC. In addition, NKILA exerts its effect as a tumor suppressor via inhibiting tumorigenesis and metastasis of NPC, and overexpressing NKILA reverses tumorigenesis and metastasis of NPC. NKILA might be a vital gene for repressing the role of EBV and become one of the most important therapeutic targets for patients with nasopharyngeal carcinoma.

In summary, NKILA plays a critical role in NPC progression. The unique histological features of NPC indicate that local inflammation is essential in NPC tumorigenesis. The present study provides new insights into the effects of inflammation on NPC biology. NKILA might be a candidate molecular marker and a novel therapy target for NPC patients.

## Methods

### Ethics statement

This study was performed in accordance with the Institutional Review Board of Sun Yat-sen University Cancer Center (GZR2016-210). Written informed consent was obtained from each patient, including signed consent for tissue analysis and consent to be recorded for potential medical research at the time of sample acquisition.

### Cell culture

S18 and S26 are subclones of NPC cell lines CNE-2, 6-10B and 5-8F are subclones of NPC cell lines SUNE-1 that were reported previously [49]. S18 and 5-8F has high metastatic potential, whereas S26 and 6-10B has low metastatic potential. All cell lines were cultured in complete RPMI 1640 medium.

### Patients and tissue specimens

Tissue specimens were obtained from the Nasopharyngeal Department of Sun Yat-sen University Cancer Center. A total number of 107 paraffin-embedded NPC and 20 normal control tissues obtained between August 1999 and February 2001 were examined in the present study. Fresh frozen normal nasopharyngeal epithelial and NPC tissues were obtained by biopsy.

### Quantitative PCR assay

Total RNA extraction and qRT-PCR were performed as we have reported[13]. Primer sequences of NKILA were as follows: sense, 5′-AACCAAACCTACCCACAACG-3′; antisense: 5′-ACCACTAAGTCAATCCCAGGTG-3′.

### Anchorage-independent growth assays

It was performed by soft agar colony formation assay and foci formation assay. Soft agar colony formation assay was performed in Six-well plates with a layer of 0.66% agar. Preparation of cells in 2 -fold concentration of 1640 complete medium and 0.33% agar, after evenly mixed and seeded at 3 different dilutions:1×10^3^, 2×10^3^,3×10^3^. After culturing for 12–14 days, clone(>50 cells) numbers were assessed at an original magnification of ×100, the scale bar is 100μm, which is approximately the diameter of 50 cell clusters. Spheres in ten random fields of view were counted each well. Foci formation assay was performed in six well plates, cells were seeded in triplicate at 3 different dilutions: 100, 200, 300. Cells were cultured for a period of 14 days. Clone (>50 cells) numbers were calculated. All experiments were repeated separately at least 3 times.

### In situ hybridization and data analyses

The expression of NKILA in paraffin-embedded samples was detected by in situ hybridization (ISH). ISH was performed and analyzed as we previously reported[21]. An SI score of 2 was used as the cut-off value, SI>2 was defined as high NKILA expression, SI≤2 was defined as low NKILA expression. The probes used in the ISH assays were as follows: NKILA: TCTCCAGACAGAATCAACTTCG; NKILA antisense: CGAAGTTGATTCTGTCTGGAGA.

### Stable overexpression of NKILA in NPC cells

S18 and S26 cells stably expressing lentiviral particles with NKILA or control vector were obtained from Gene Pharma (Shanghai, China). For cell transduction, NPC cells (30–50% confluency) were infected according to the instructions. After an incubation of 12–16 h, the medium was changed. Forty-eight hours later, the selection reagent puromycin (Sigma-Aldrich, St. Louis, MO) was used to select stably transfected clones. After 21days of continuous selection, cells infected with LV5-NKILA were designated S26 NKILA, cells infected with LV5-NC were designated S26 Vec.

### Dual luciferase reporter assay

Luciferase activity was determined by the Dual-Luciferase Reporter Assay System (E1910, Promega, Madison, WI)). Cells were transfected with the pGL3-basic or pNFκB-luc constructs together with pRL-TK at 50:1, and then then the indicated treatments were performed. Cells were harvested and assayed 24h later, and all of the experiments were performed in triplicate.

### Boyden chamber set-up for migration assay

2 × 10^4^ NPC cells in 100μl RPMI 1640 medium with 2% FBS were plated in the upper chamber of transwell (Corning, New York, NY, USA), the bottom chamber was filled with 600μl RPMI 1640 medium with 10% FBS. After 20h of incubation, fixed the cells on the lower membrane surface in 4% paraformaldehyde and stained with crystal violet, then counted. The cells were counted in ten random optical fields (×200 magnification), the average number was obtained from triplicate filters. Data are shown as the average ±SD. For the invasion assay, coated the upper chamber with Basement Membrane (R&D, Minneapolis, MN, USA) first. All experiments were performed at least three times.

### Experimental tumorigenesis and metastasis assay *in vivo*

Female BALB/c(nu/nu) nude mice (4–6 weeks of age) were used. For tumorigenesis experiments, cancer cells (10^5^/mouse) were injected subcutaneously into the flank of the mouse, and measured the tumor size every two days. Calculated the tumor volumes as follows: tumor volume (mm^3^) = length×width^2^×0.5. Metastasis of NKILA-overexpressing cancer cells and control cells was evaluated by tail vein intravenous injection. 4 weeks after the first inoculation, the experiment was terminated. After euthanasia, the lungs of mouse were harvested and weighed separately, then prepared for H&E staining. Metastatic nodules in mouse was counted respectively.

### Immunofluorescence (IF)

1 × 10^3^ S26 cells overexpressing NKILA or NKILA shRNA were cultured on coverslips overnight prior to the experiment. After fixing with 4% paraformaldehyde, IF was done and imaged as previously reported [50], primary antibodies against P65 was used, followed by FITC-conjugated secondary antibodies (Invitrogen, Carlsbad, CA). Sc-3060(Santa Cruz, CA) and JSH-23(Millipore, Billerica, MA) are drugs which inhibit NF-kB nuclear translocation. 30min before specified treatment,10uM Sc-3060 and 5uM JSH-23 were added into the culture.

### Statistical analyses

Statistical analyses were done by SPSS 18.0. Correlation between NKILA expression and clinicopathological features was analyzed by chi-square test. The survival data were plotted and analyzed by Kaplan-Meier, log-rank test, and multivariate Cox regression analyses. All experiments for cell culture were performed in triplicate at least three times. The data were shown as means ± SD. P values were calculated by Student's t-test. A P value of no more than 0.05 was considered statistically significant.

## Supporting information

S1 Fig Related to Fig 6. Efficiencies of NKILA overexpressing in S18 cells and NKILA-shRNA in S26 cells.
**(A)**Expression of NKILA in S18 stably expressing pcDNA3.1 vector control or NKILA determined by qRT-PCR assay (Mean ± SD, ***, P < 0.001 versus S18 vec PBS). **(B)**Expression of NKILA in S26 with stably depletion of ctrl or NKILA examined by qRT-PCR assay (Mean ± SD, ***, P < 0.001 versus S26 shvec TNFα, ###, P < 0.001 versus shvec PBS).
(TIF)

S2 Fig Related to Fig 6. The expression of P65/P50 in cytoplasmic and nuclear fraction examined by western-blot.
(A)The expression of P65/P50 in nuclear of S26. (B) The expression of P65/ P50 in cytoplasm of S26.
(TIF)
