# Expression of Concern: *NKILA represses nasopharyngeal carcinoma carcinogenesis and metastasis by NF-κB pathway inhibition*

**DOI:** 10.1371/journal.pgen.1010332

**Published:** 2022-08-16

**Authors:** The PLOS Genetics Editors

Following publication of this article [1], the authors requested correction to address errors in the assembly of Figs 4A, 5A, 5B and 6E by providing replacement image panels and underlying data.

**Fig 4:**
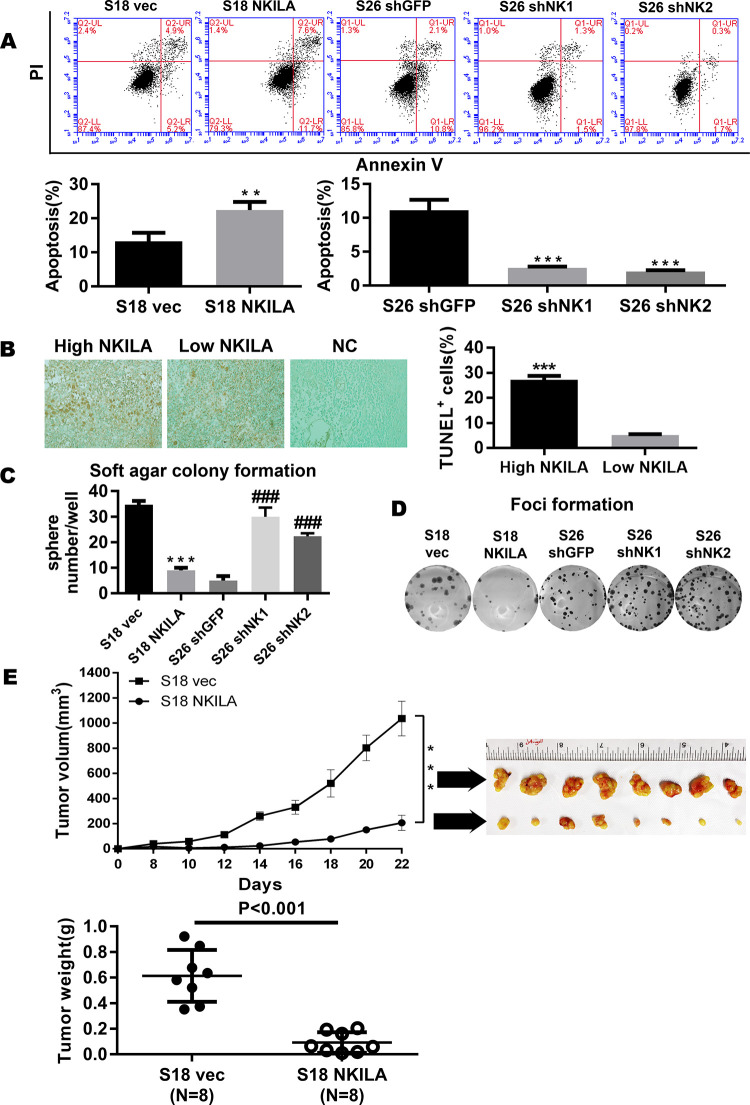
NKILA represses the tumorigenesis of NPC. (A) Apoptosis of control vector or NKILA-overexpressing S18 cells and shGFP or shNKILA S26 cells as assayed by ANNEXIN-V/PI staining (mean±SD, n = 3,***<0.001 versus S26 shGFP,**<0.01versus S18 vec). (B) Tunnel assay revealing the correlation between NKILA and apoptosis in 65 paraffin-embedded NPC specimens. (C) Anchorage-independent growth of control vector or NKILA-overexpressing S18 cells and shGFP or shNKILA S26 cells in soft agar. Over 14 days of culture, number of colonies was calculated at 10 randomly ten fields of view. Original magnification, ×100. (Error bar represent SD. Mean ± SD, n = 3, ***, P < 0.001 versus S18 vec; ###, P < 0.001 versus S26 shGFP). (D) Foci formation by control vector or NKILA-overexpressing S18 cells and shGFP or shNKILA S26 cells were performed as described in the Methods. (E) Tumor formation in nude mice: S18 (vec, NKILA) cells were injected and tumor volumes were calculated and plotted as described in the Methods. The upper panel shows the tumor growth and tumor volume in mice (S18 vector control, S18 NKILA overexpressing cells). The tumor weight is also shown in the lower panel, and tumors treated with vec are much heavier, indicating that NKILA can inhibit tumor growth.

**Fig 5:**
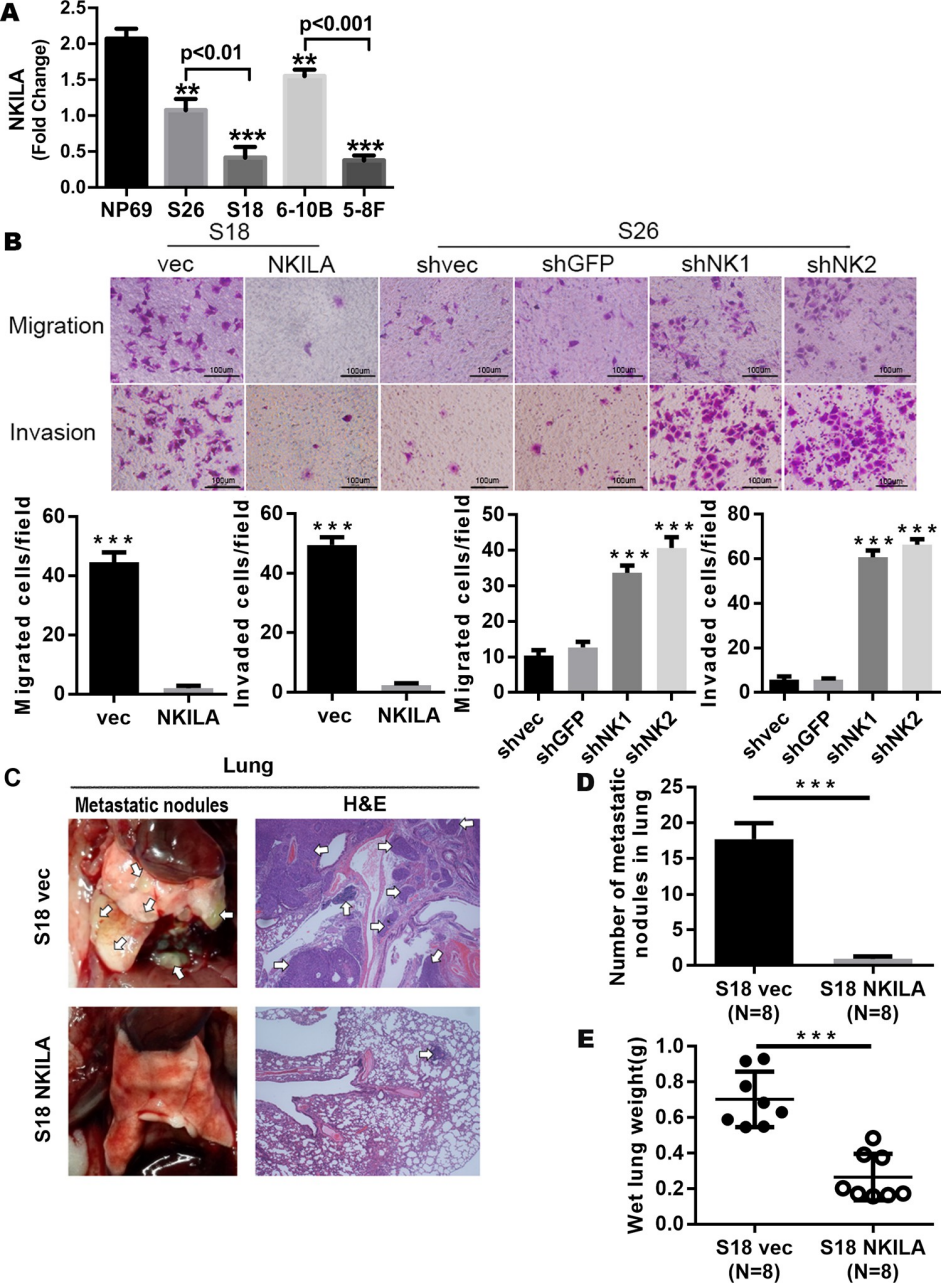
NKILA repress the metastasis of NPC by inhibiting the NF-kB pathway. (A) NKILA expression in NPC cell lines determined by qRT-PCR assay (Mean ± SD, n = 3, ***, P < 0.001, **, P < 0.01versus NP69). (B) Representative images of migrated and invaded NPC cells with overexpressed or silenced NKILA in Boyden chamber assay (Mean ± SD, n = 3, ***, P < 0.001, versus control). (C) Representative images of the lung metastasis, metastatic nodes were indicated by Arrowheads. Left: Representative lungs, Right: Representative image (×200) of lung with metastasis by H&E staining. (D) 6 weeks after tail vein injection, number of metastases in lungs of each mice (mean±SD) were calculated. (E) The wet lung weight of the mice treated with the control vehicle was significantly higher, suggesting that mouse in control group had more or greater metastases, indicating that NKILA inhibited tumor metastasis.

**Fig 6:**
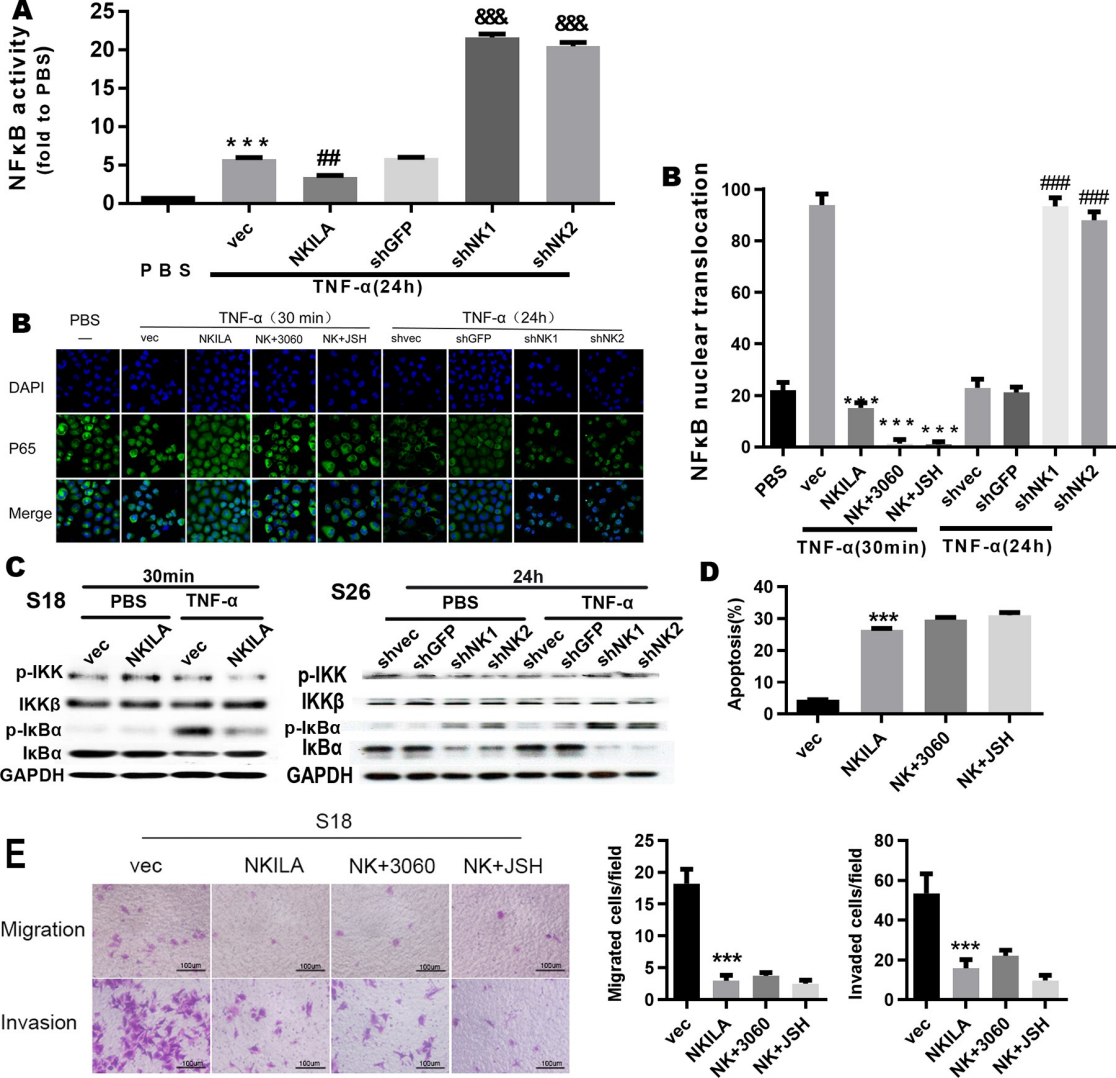
NKILA inhibits the activation of NF-κB by repressing IκB phosphorylation. **(A)** Luciferase reporter assay for detection of NF-κB activity in S26 cells treated with TNF-α (mean ± SD, n = 3, ^**&&&**^, P < 0.001 versus shGFP; ^**##**^, P < 0.01 versus vec; ***, P < 0.001 versus PBS). **(B)** Immunofluorescence confocal microscopy for detection of P65 nuclear translocation in S26 cells with overexpression or depletion of NKILA treated with TNF-α (mean ± SD, n = 3,***, p < 0.001 versus vec, ###, P < 0.001 versus shvec).**(C)** Total and phosphorylated IKK and IκBα assayed by Western blotting in S26 or S18 cells. **(D)** Annexin-V/PI staining for detection of apoptosis in S18 cells stably expressing NKILA (48 h after seeding). (mean ± SD, n = 3, ***, P < 0.001 versus vec; 3060:10 µM; JSH:5 µM). **(E)** Migration and invasion in S18 cells with stably overexpression or depletion of NKILA, assayed by Boyden Chamber assay (16h for migration and 22 h for invasion after seeding, respectively) (mean ± SD, n = 4, ***, P < 0.001 versus S18 vec).

During editorial follow up on these issues, it came to the attention of the *PLOS Genetics* Editors that the nasopharyngeal carcinoma cell lines used in the *in vitro* and *in vivo* experiments reported in Figs 4–6 have been reported elsewhere to be possibly contaminated or misidentified (S18 and S26 are subclones of CNE-2; 6-10B and 5-8F are subclones of SUNE-1; see ICLAC Register of Misidentified Cell Lines (iclac.org/databases/cross-contaminations/) [2], Cellosaurus (https://web.expasy.org/cellosaurus/) [3] and [4–6]). One of the corresponding authors has provided cell line authentication reports dated 18 September 2017 for cell lines S18 and S26, including short tandem repeat (STR) profiles (S1 File); however, the *PLOS Genetics* Editors consider that the data provided do not fully resolve the concerns.

The following figure corrections are based on information provided by one of the corresponding authors regarding errors in Figs 4, 5, and 6:

Errors were made in the assembly of Figs 4A, 5A-B, and 6E, resulting in the incorporation of incorrect representative images in these figures.

In the originally published Fig 4A, the S26 shNK2 panel is incorrect. The flow cytometry data for this experiment underwent a second analysis because of a software issue encountered during the first analysis. The incorrect panel was included during figure assembly by mistake and shows the exported dot plot from the first analysis of S26 shNK1. Here the authors provide a revised Fig 4 using the correct S26 shNK2 panel from the original experiment. Dot plots for all of the replicates (n = 3) underlying Fig 4A are provided as Supporting Information S2 File. The raw.fcs files underlying the representative dot plot panels in Fig 4A are provided as Supporting Information S3 File.

In the originally published Fig 5A, there is an error in the p value for the comparison between S26 and S18. The correct p value is <0.01.

In the originally published Fig 5B, there is a region of overlap between the Invasion panels for shNK1 and shNK2. The shNK2 panel is incorrect and was included during figure assembly by mistake. Here the authors provide a revised Fig 5 using the correct shNK2 Invasion panel from the original experiment. The underlying image files for all replicates (n = 3) for Fig 5B are provided as Supporting Information S4 File.

There are errors in the figure legend for Fig 6. Units of drug concentration are incorrectly reported as mM for Fig 6D. The correct unit is µM. Additionally for Fig 6E, the number of replicates is incorrectly reported as n = 3. The correct number of replicates for this experiment is n = 4.

In the originally published Fig 6E, there is a region of overlap between the Migration panels for NK+3060 and NK+JSH. The Migration NK+JSH panel is incorrect and was included during figure assembly by mistake. Here the authors provide a revised Fig 6 using the correct Migration NK+JSH panel from the original experiment and a corrected figure legend to address the above issues. The underlying image files for all replicates (n = 4) for Fig 6E are provided as Supporting Information S5 File.

The quantitative data underlying the charts in Figs 4A, 5A, 5B and 6E are provided as Supporting Information S6 File.

The corresponding author has additionally clarified that the Fig 2A cancer NKILA ISH panel and the Fig 2B NPC stage I NKILA ISH panel are both correct and use the same image showing a representative example of Stage I nasopharyngeal carcinoma.

Methodological information for the flow cytometry experiments was not provided in the original article. Additional information is provided as follows:

Apoptosis was performed by using Annexin V Apoptosis Detection Kit (eBioscience). Cells were dissociated by 0.25% trypsin-EDTA and harvested by centrifugation at 300g for 5min. And then cell pellets were resuspended in 200ul binding buffer containing 5ul Annexin V antibody and incubated for 15min at room temperature. After that, cells were washed and resuspended in 200ul binding buffer containing 5ul propidium and analyzed by flow cytometry.

An evaluation of the revised figures by a member of the *PLOS Genetics* Editorial Board determined that the corrected figures and the accompanying replicate dot plots, and microscopy image files, support the reported results of the article. However, in light of the cell line concerns, the *PLOS Genetics* Editors issue this Expression of Concern to alert readers to the use of cell lines previously reported to be misidentified/contaminated, which may affect whether the findings are representative of nasopharyngeal carcinoma biology.

## Supporting information

S1 File Cell Line Authentication Reports. (ZIP)

S2 File Fig 4A dot plots. (PPTX)

S3 File Fig 4A raw.fcs files. (RAR)

S4 File Fig 5B replicate images. (ZIP)

S5 File Fig 6E replicate images. (ZIP)

S6 File Quantitative data. (XLSX)
