# Supplementary figures and images for: NKILA represses nasopharyngeal carcinoma carcinogenesis and metastasis by NF-κB pathway inhibition
Source: PLoS Genet. 2019 Aug 20;15(8):e1008325. doi: 10.1371/journal.pgen.1008325 (PMC6716677; doi:10.1371/journal.pgen.1008325)

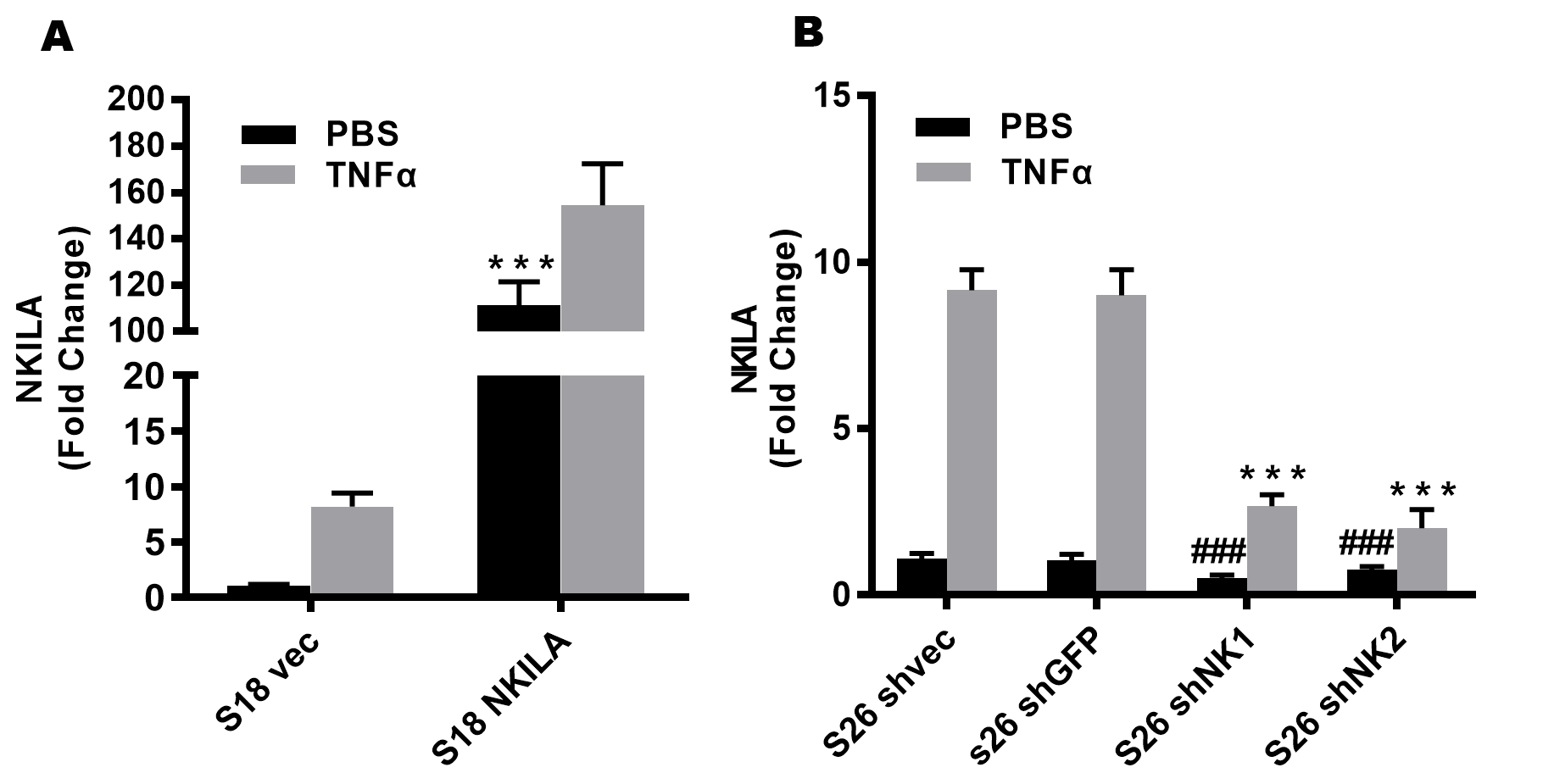

Supplement: S1 Fig — Efficiencies of NKILA overexpressing in S18 cells and NKILA-shRNA in S26 cells. (A)Expression of NKILA in S18 stably expressing pcDNA3.1 vector control or NKILA determined by qRT-PCR assay (Mean ± SD, ***, P < 0.001 versus S18 vec PBS). (B)Expression of NKILA in S26 with stably depletion of ctrl or NKILA examined by qRT-PCR assay (Mean ± SD, ***, P < 0.001 versus S26 shvec TNFα, ###, P < 0.001 versus shvec PBS). (TIF) [file pgen.1008325.s001.tif]

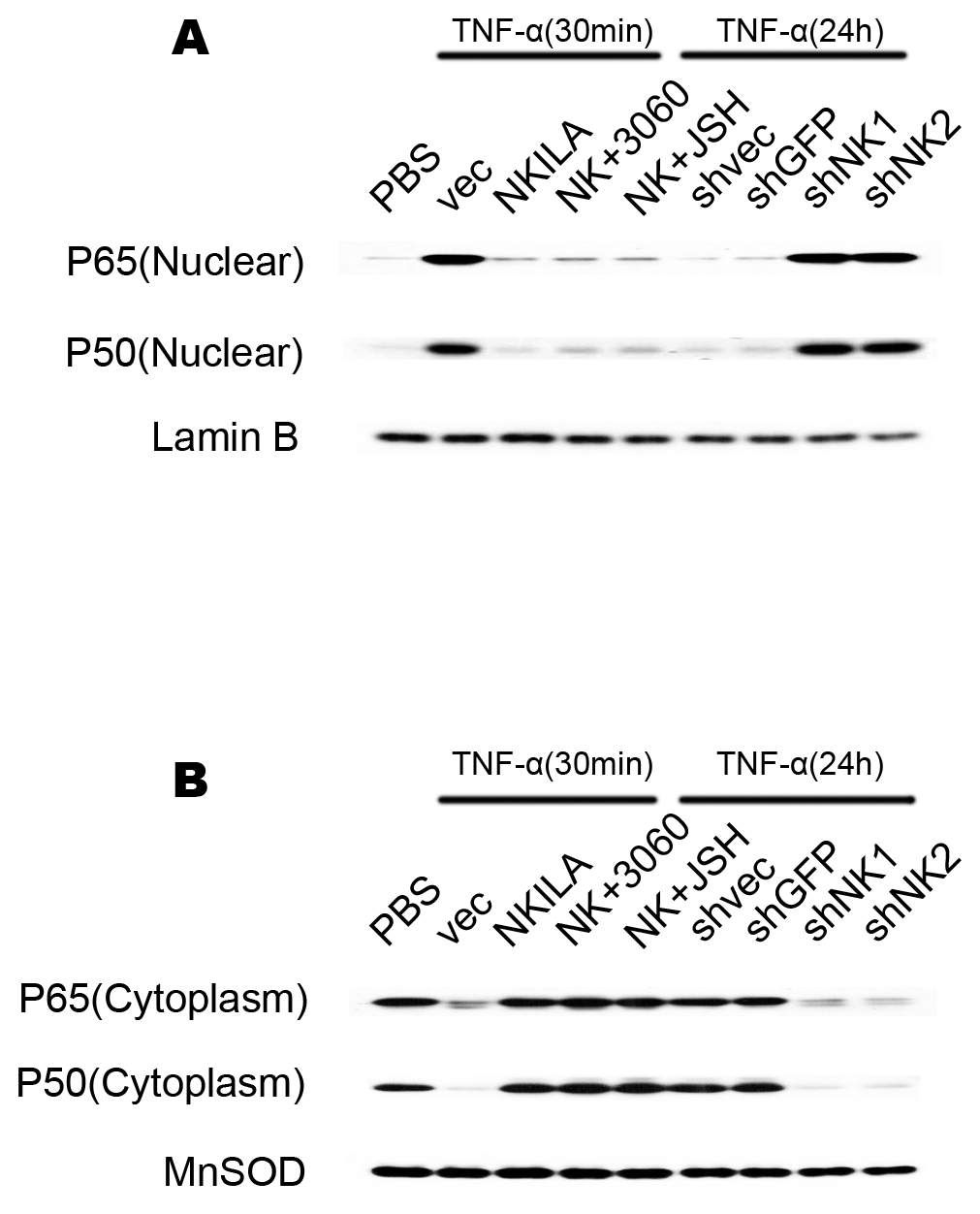

Supplement: S2 Fig — The expression of P65/P50 in cytoplasmic and nuclear fraction examined by western-blot. (A)The expression of P65/P50 in nuclear of S26. (B) The expression of P65/ P50 in cytoplasm of S26. (TIF) [file pgen.1008325.s002.tif]
