# Supplementary material for: Expression of Concern: NKILA represses nasopharyngeal carcinoma carcinogenesis and metastasis by NF-κB pathway inhibition
Source: PLoS Genet. 2022 Aug 16;18(8):e1010332. doi: 10.1371/journal.pgen.1010332 (PMC9380937; doi:10.1371/journal.pgen.1010332)
Supplement: S1 File — (ZIP) [file pgen.1010332.s001.zip › Excerpt of S18 Cell Line Authentication Report.pdf]

Figure 1. STR profiles of S18 cell line

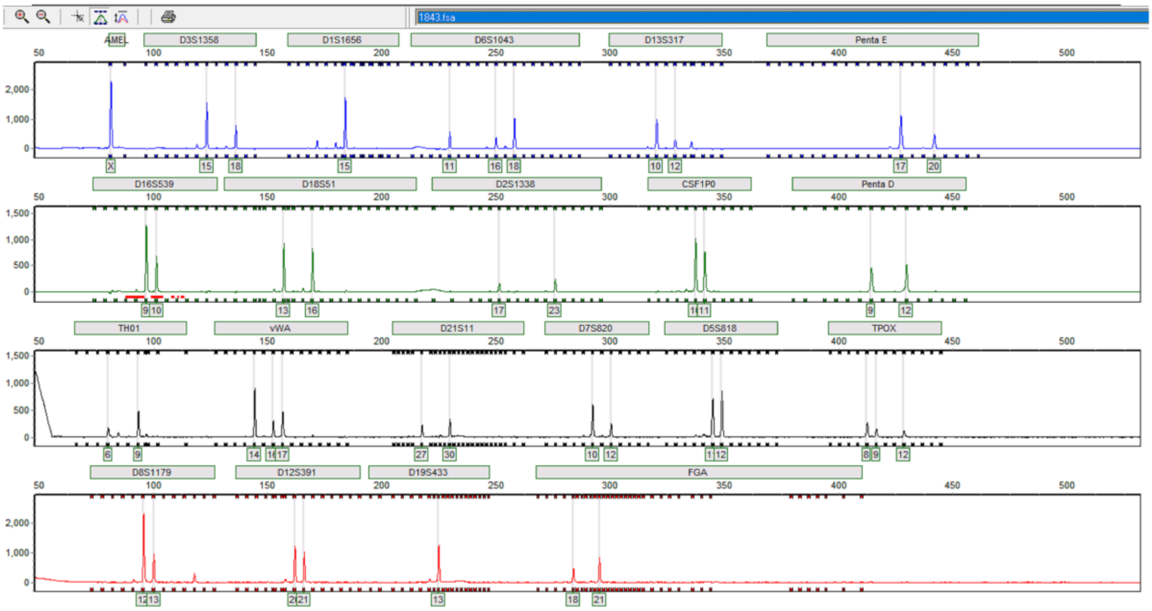

Table 1. STR profiles of S18 cell line

	Allele1	Allele2	Allele3
AMEL	X		
D3S1358	15	18	
D1S1656	15		
D6S1043	11	16	18
D13S317	10	12	
Penta E	17	20	
D16S539	9	10	
D18S51	13	16	
D2S1338	17	23	
CSF1PO	10	11	
Penta D	9	12	
TH01	6	9	
vWA	14	16	17
D21S11	27	30	
D7S820	10	12	
D5S818	11	12	
TPOX	8	9	12
D8S1179	12	13	
D12S391	20	21	
D19S433	13		
FGA	18	21	

Figure 2. Search result in DSMZ database

Result of STR matching analysis by your data.

- DSMZ Profile Database -

A graphical presentation is shown at the bottom of this page.

EV	Cell No.	Cell name	Locus names									Figures
			D5S818	D13S317	D7S820	D16S539	VWA	TH01	AM	TPOX	CSF1PO	
	Query (Your Cell)		*11,12*	*10,12*	*10,12*	*9,10*	*14,16,17*	*6,9*	*x,x*	*8,9,12*	*10,11*	

Figure 3. Authentication of the species of the sample

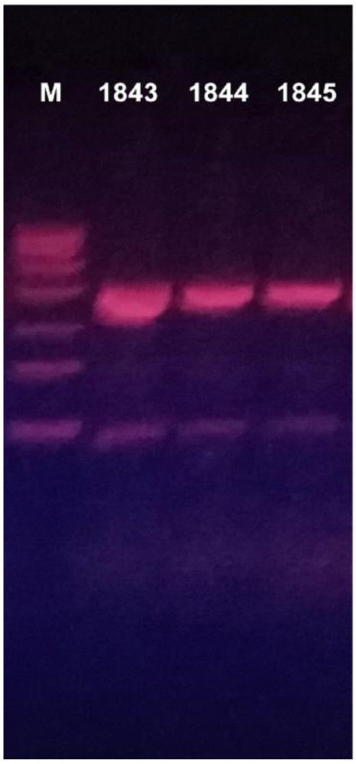

M: Marker. As the size of 700, 600, 500, 400, 300, 200 and 100bp from up to down.

Nine species are checked, as follow: *Homo sapiens* 391bp, *Cricetulus griseus*315bp, *Macaca mulatta*287bp, *Cercopithecus aethiops*222bp, *Rattus norvegicus*196bp, *Canis familiaris*172bp, *Mus musculus*150bp, *Bos Taurus*102bp, IC 70bp

JD1843: The sample. The band size is 391bp which matches the size of human.
