# Supplementary material for: Expression of Concern: NKILA represses nasopharyngeal carcinoma carcinogenesis and metastasis by NF-κB pathway inhibition
Source: PLoS Genet. 2022 Aug 16;18(8):e1010332. doi: 10.1371/journal.pgen.1010332 (PMC9380937; doi:10.1371/journal.pgen.1010332)
Supplement: S2 File — (PPTX) [file pgen.1010332.s002.pptx]

## Slide 1
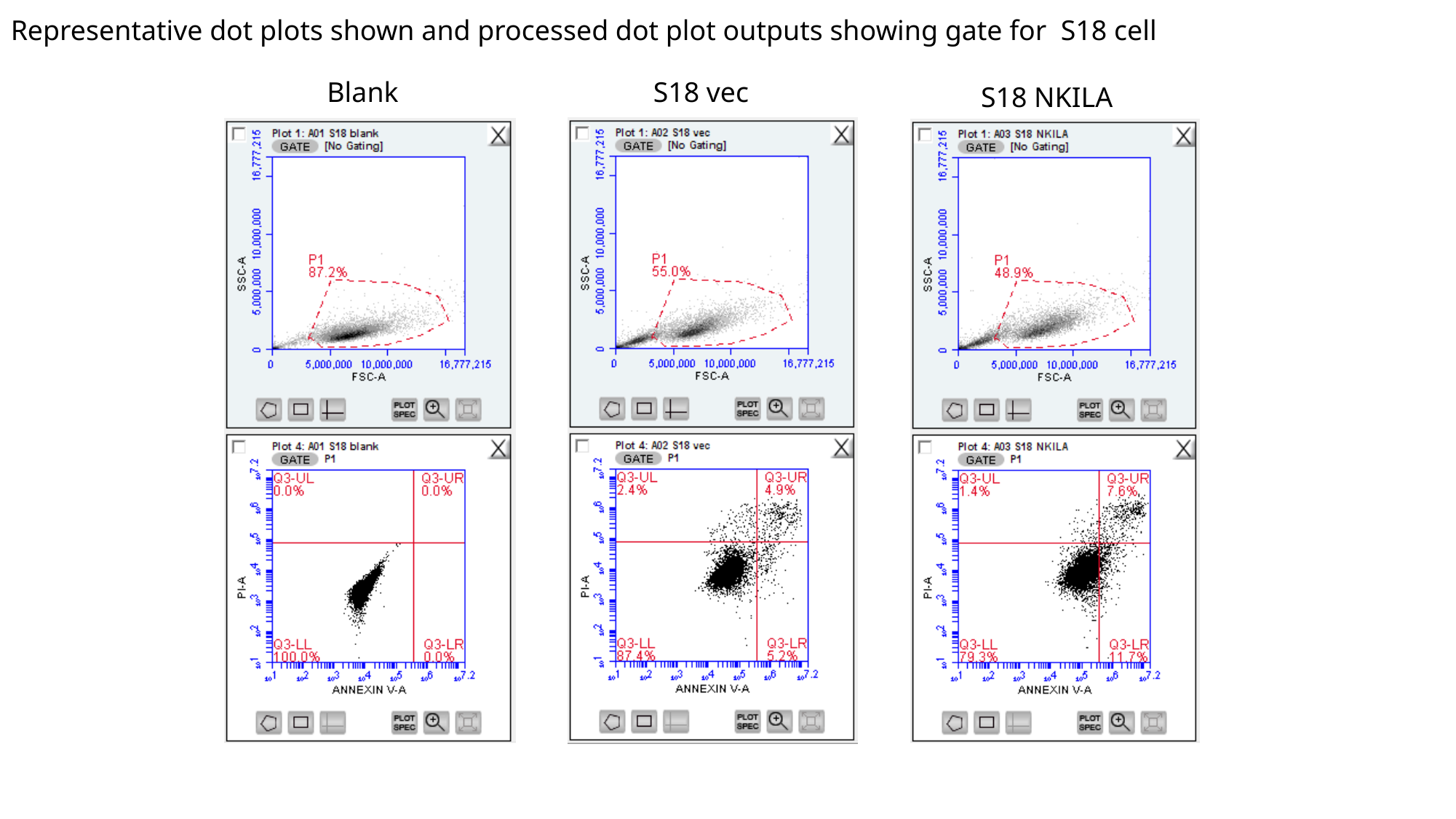

Representative dot plots shown and processed dot plot outputs showing gate for S18 cell
Blank
S18 vec
S18 NKILA

## Slide 2
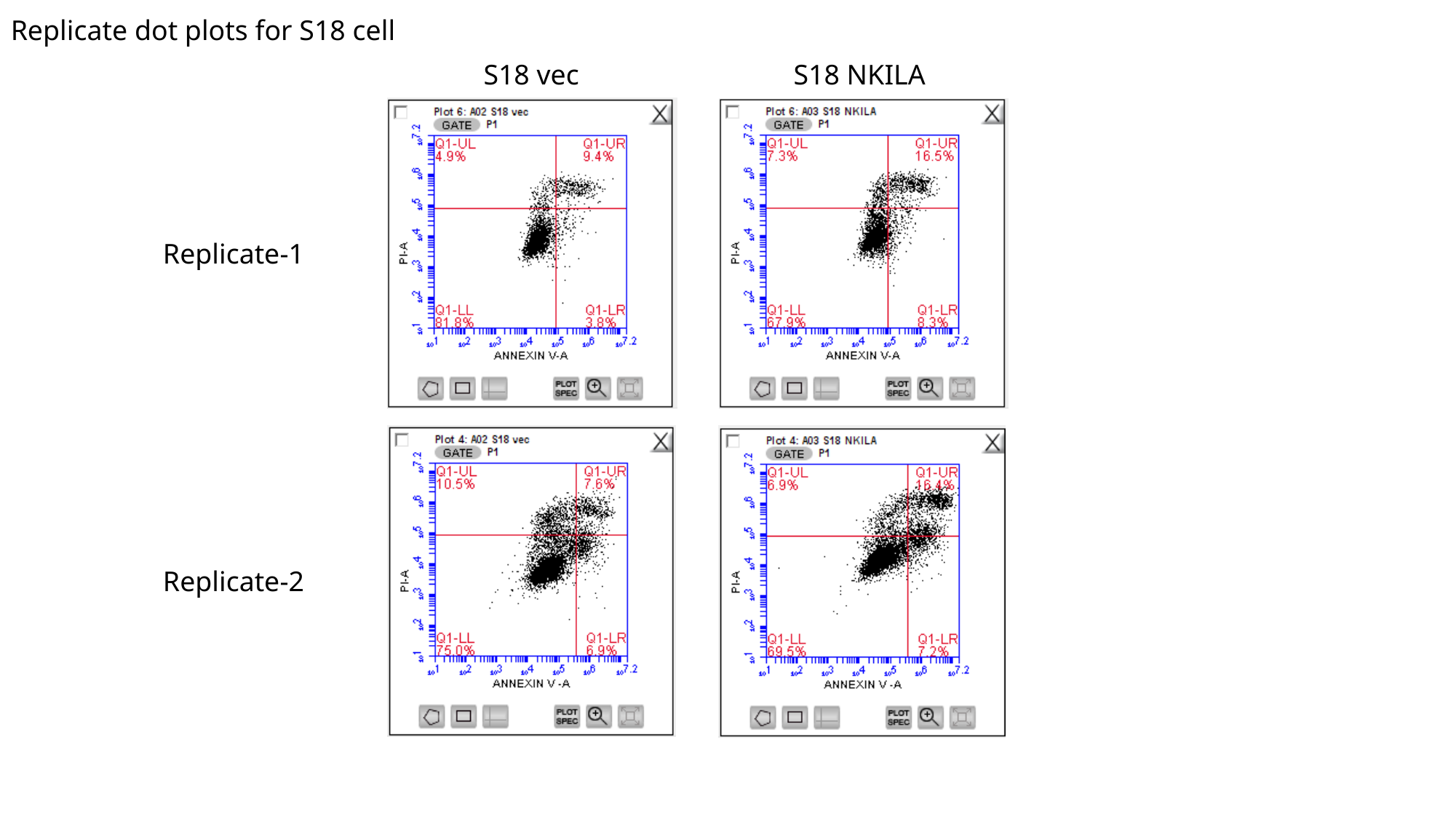

Replicate dot plots for S18 cell
S18 vec
S18 NKILA
Replicate-1
Replicate-2

## Slide 3
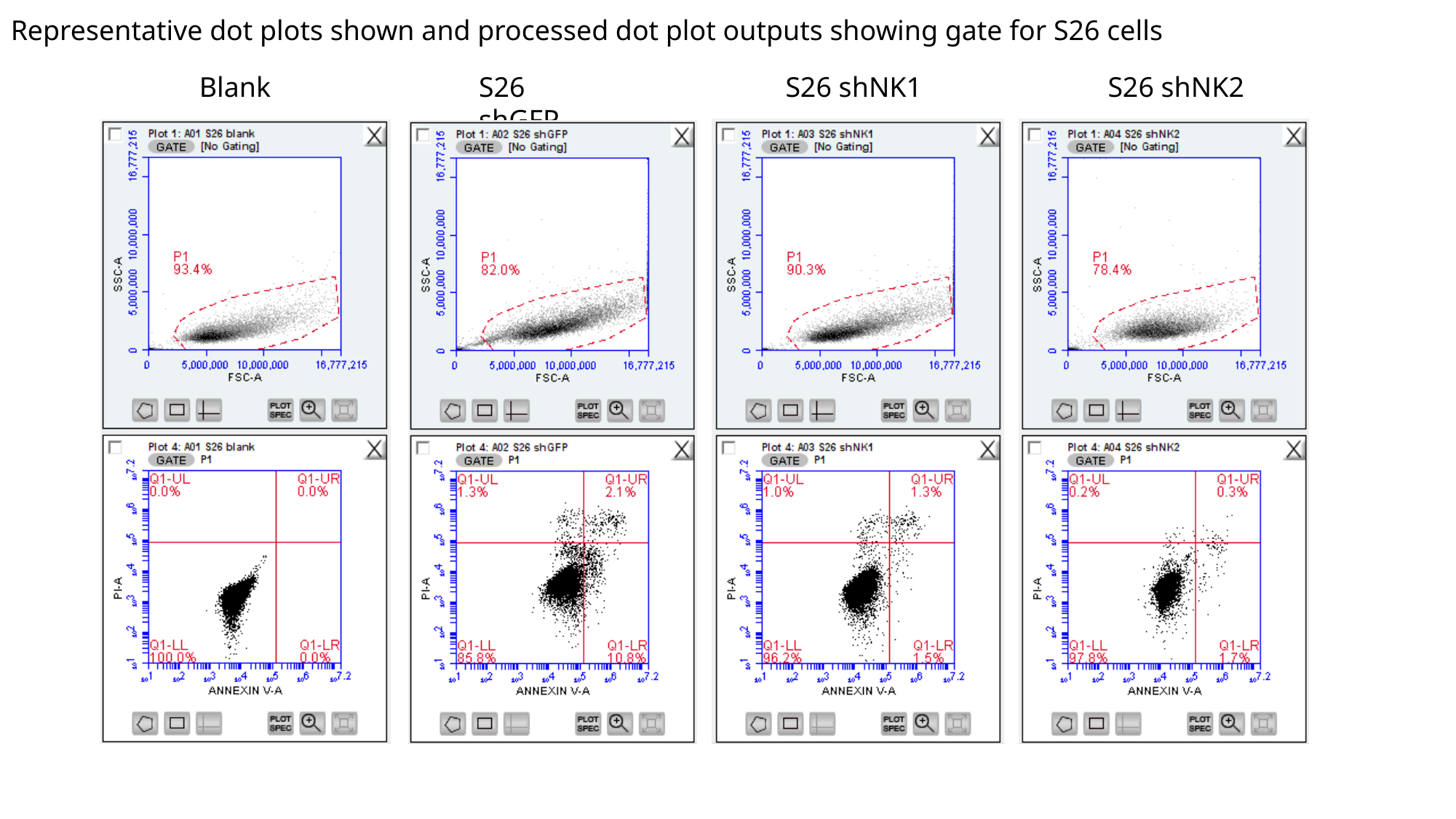

Representative dot plots shown and processed dot plot outputs showing gate for S26 cells
Blank
S26 shGFP
S26 shNK1
S26 shNK2

## Slide 4
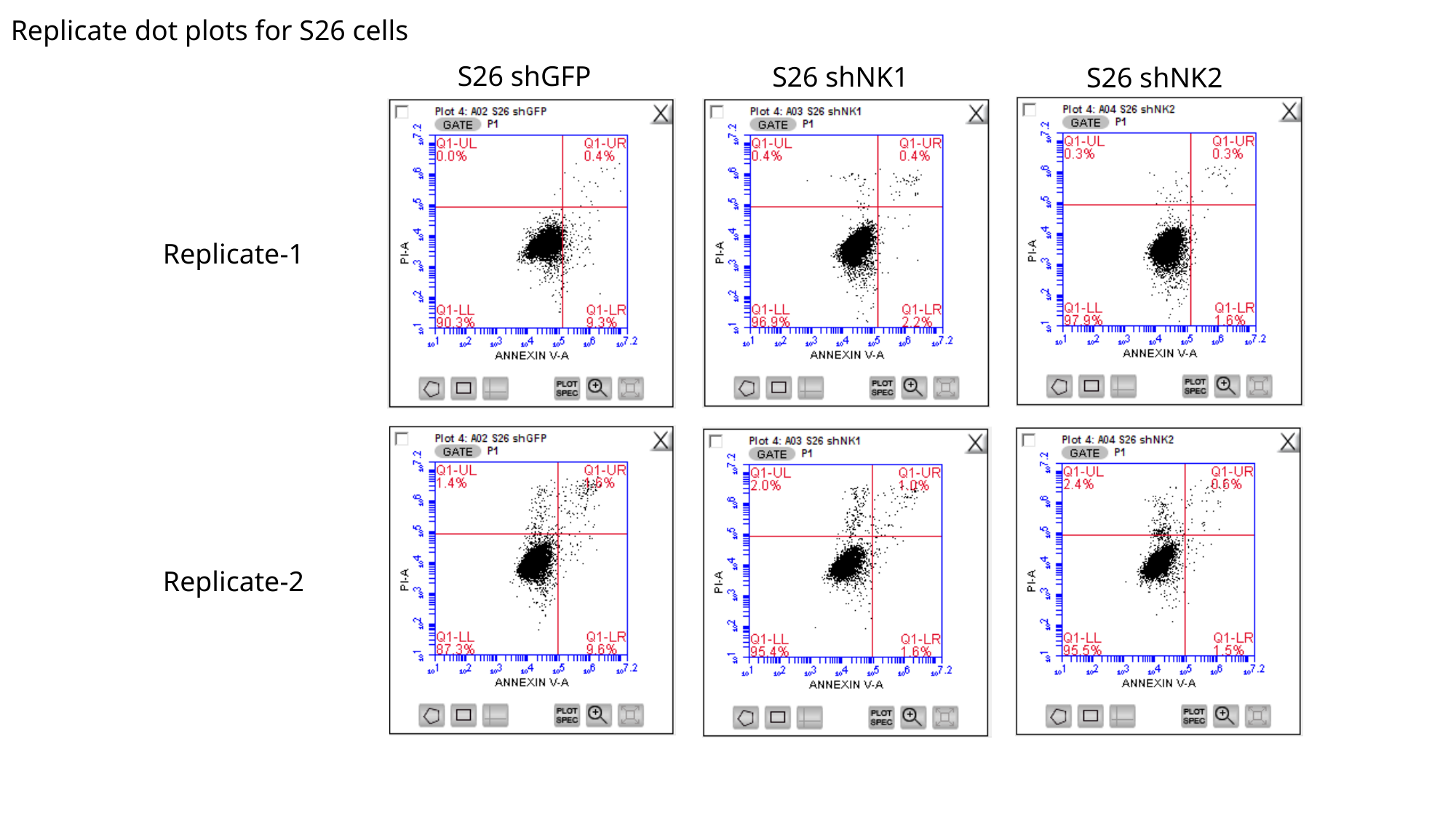

Replicate dot plots for S26 cells
S26 shGFP
S26 shNK1
S26 shNK2
Replicate-1
Replicate-2
