# Supplementary figures and images for: Expression of Concern: NKILA represses nasopharyngeal carcinoma carcinogenesis and metastasis by NF-κB pathway inhibition
Source: PLoS Genet. 2022 Aug 16;18(8):e1010332. doi: 10.1371/journal.pgen.1010332 (PMC9380937; doi:10.1371/journal.pgen.1010332)

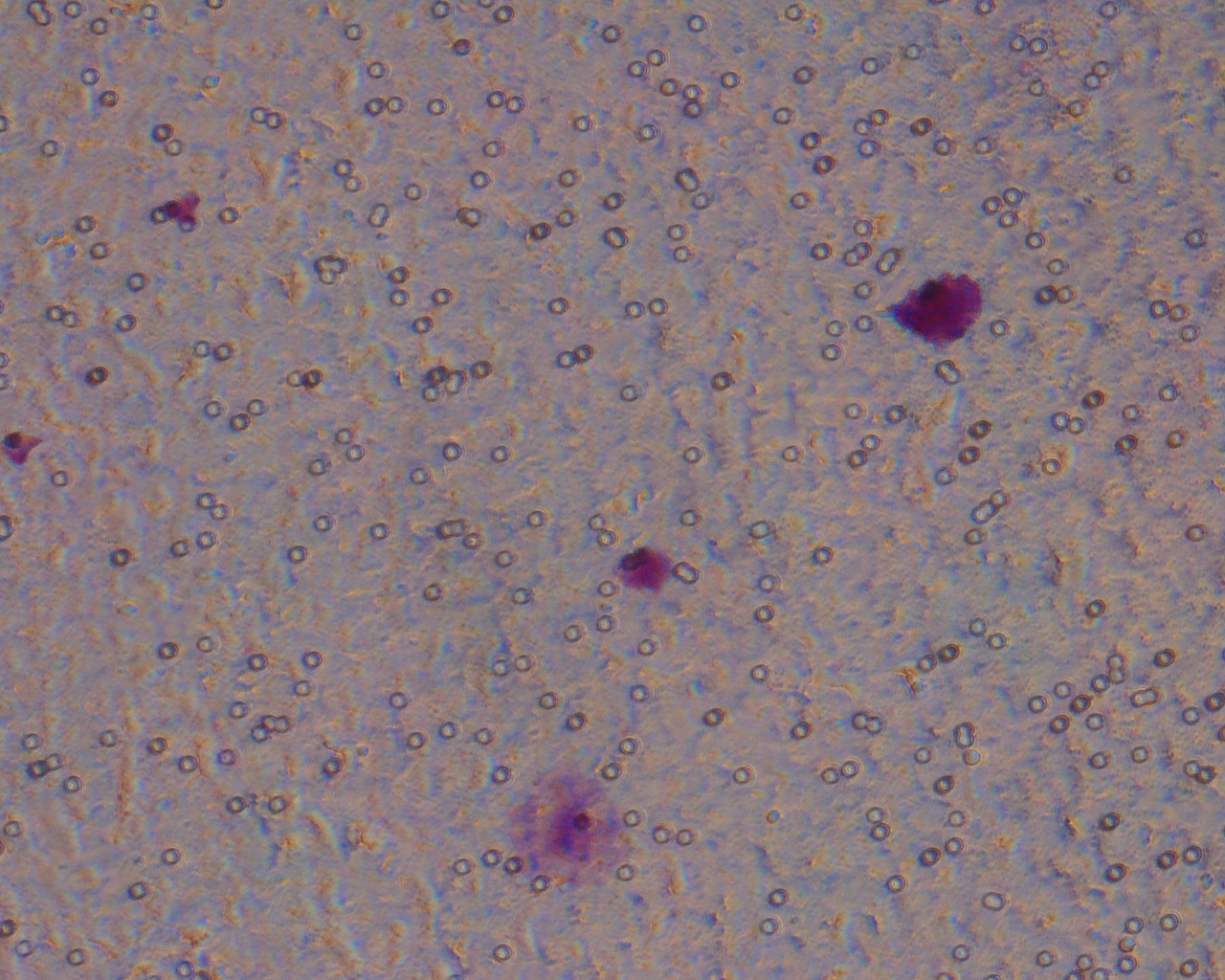

Supplement: S4 File — (ZIP) [file pgen.1010332.s004.zip › S4 File/S18 Invasion NKILA.tif]

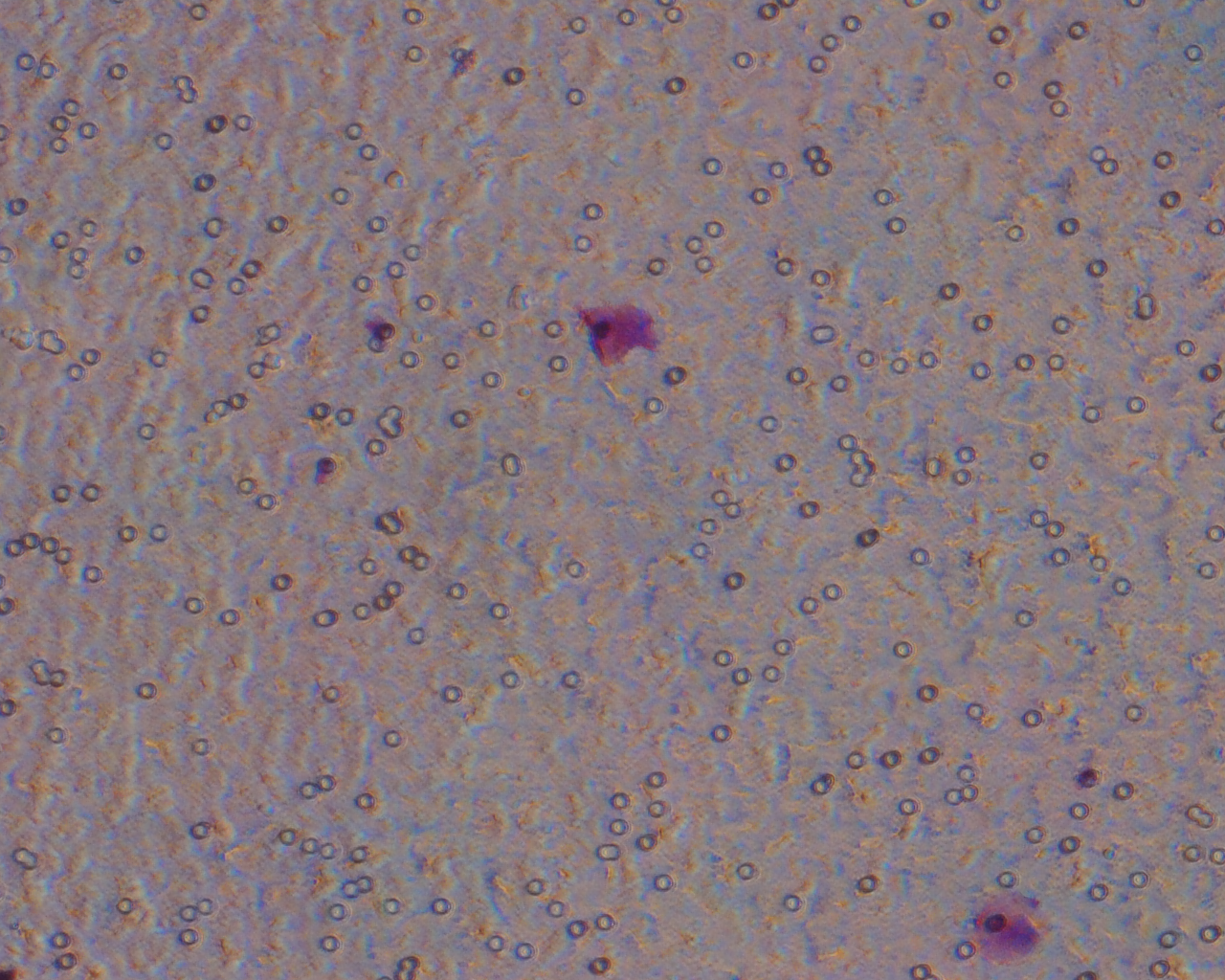

Supplement: S4 File — (ZIP) [file pgen.1010332.s004.zip › S4 File/S18 Invasion NKILA-1.tif]

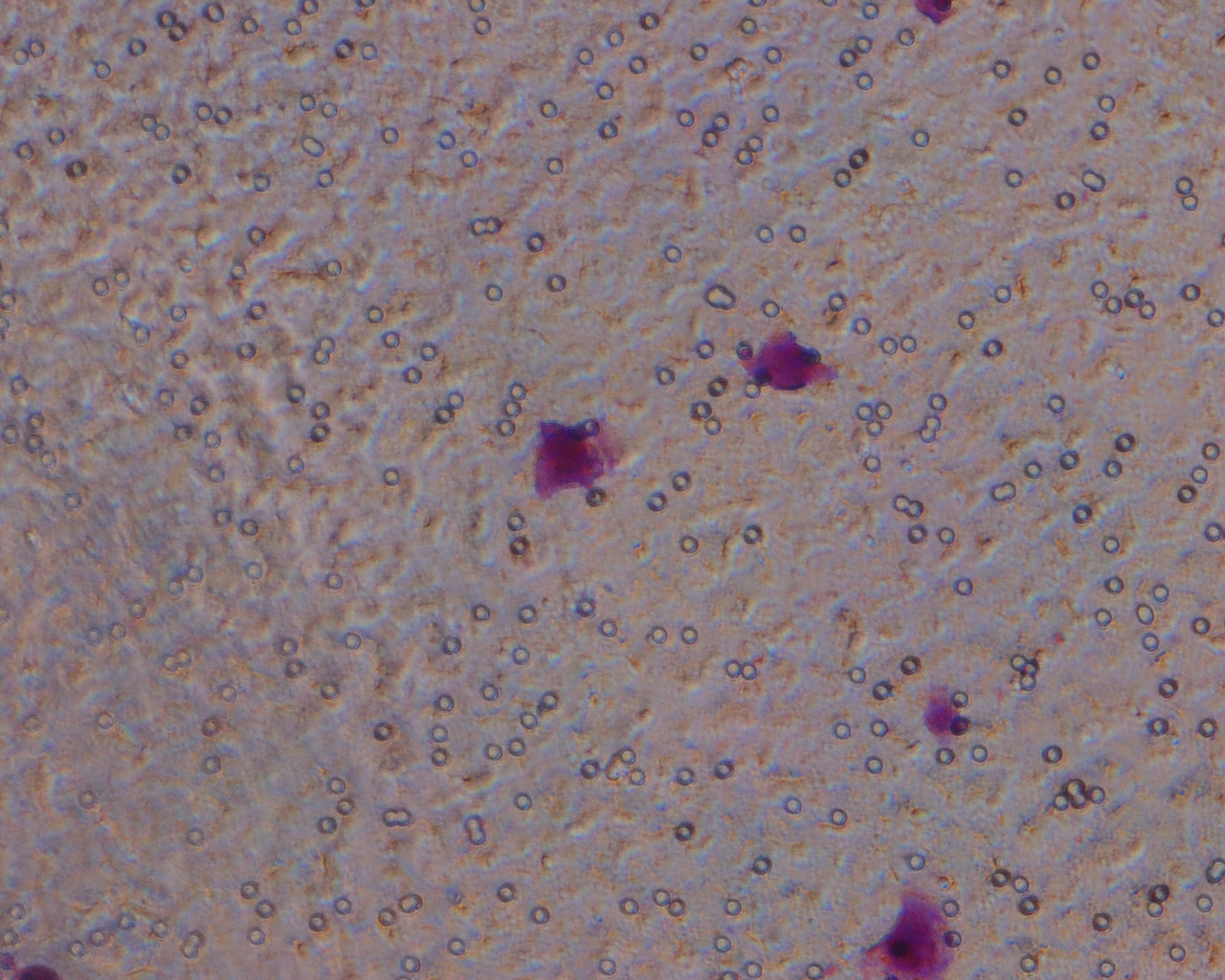

Supplement: S4 File — (ZIP) [file pgen.1010332.s004.zip › S4 File/S18 Invasion NKILA-2.tif]

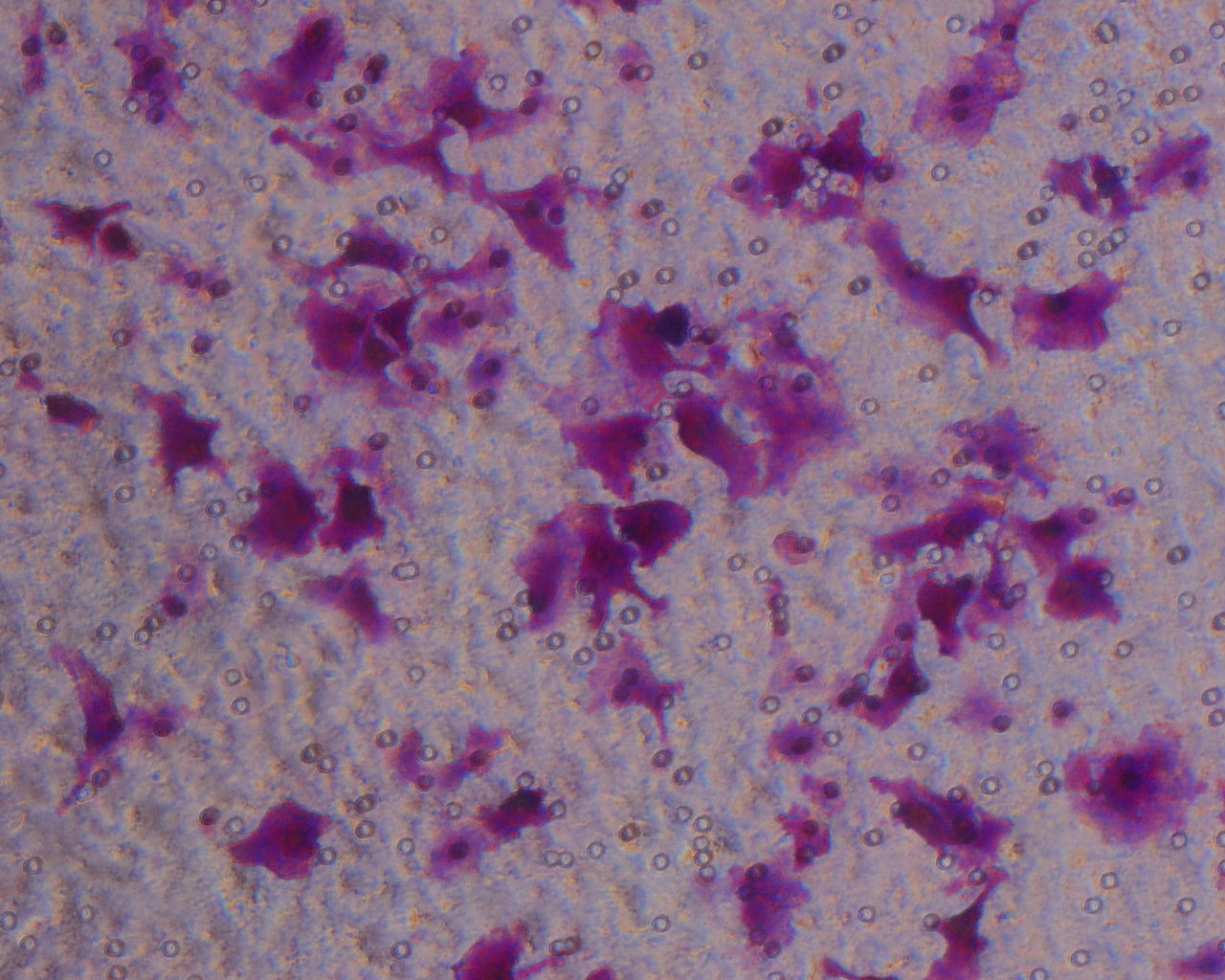

Supplement: S4 File — (ZIP) [file pgen.1010332.s004.zip › S4 File/S18 Invasion vec.tif]

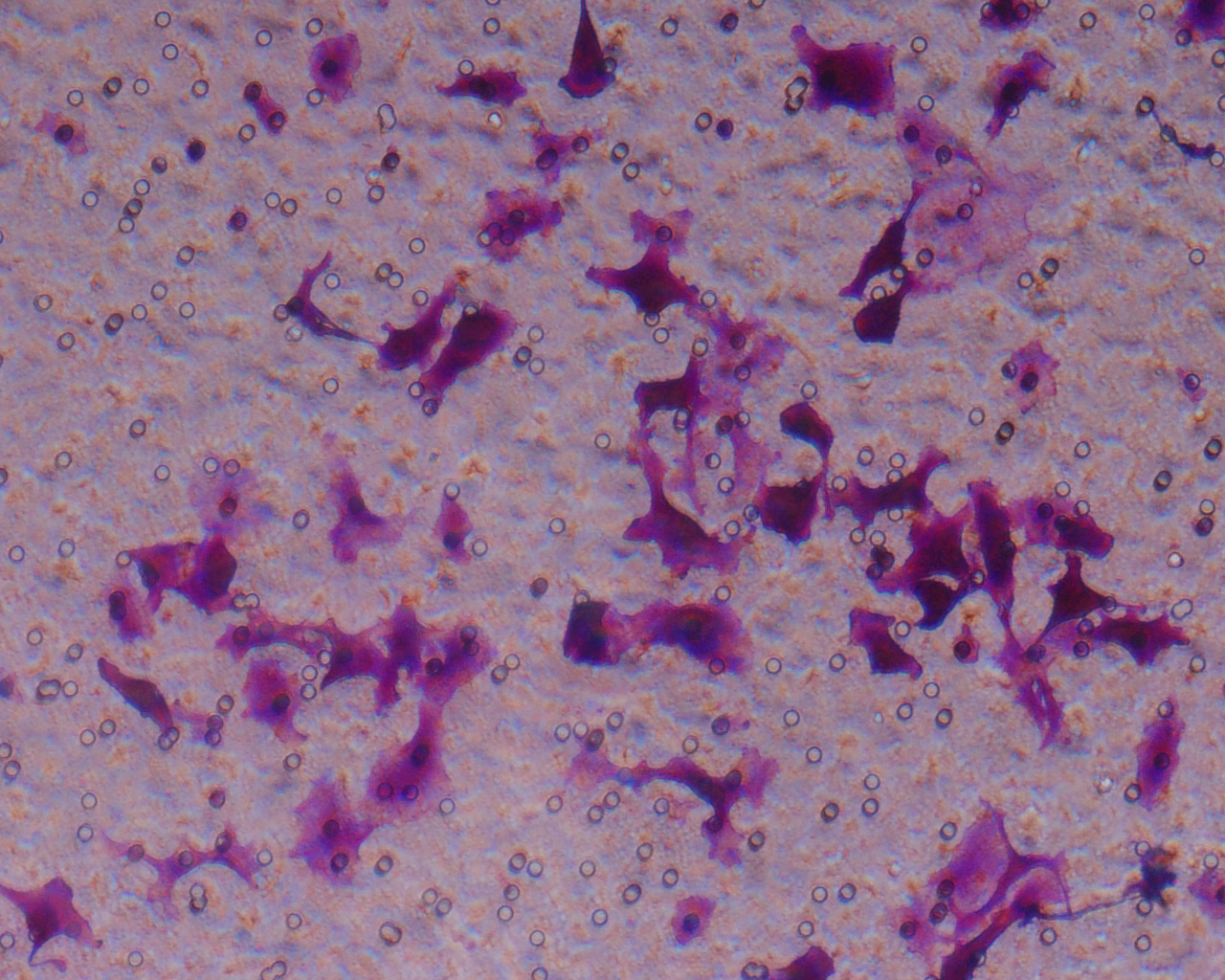

Supplement: S4 File — (ZIP) [file pgen.1010332.s004.zip › S4 File/S18 Invasion vec-1.tif]

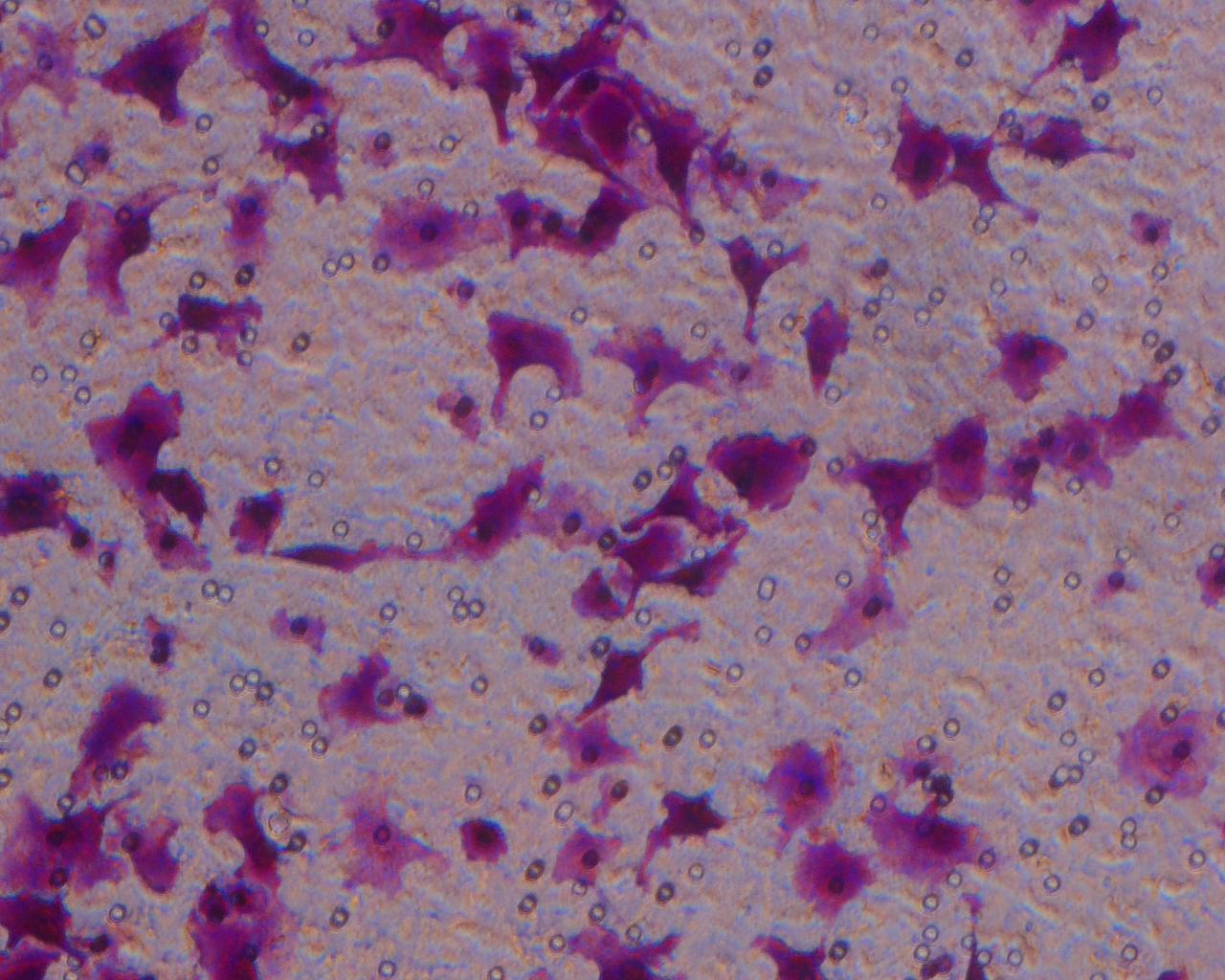

Supplement: S4 File — (ZIP) [file pgen.1010332.s004.zip › S4 File/S18 Invasion vec-2.tif]

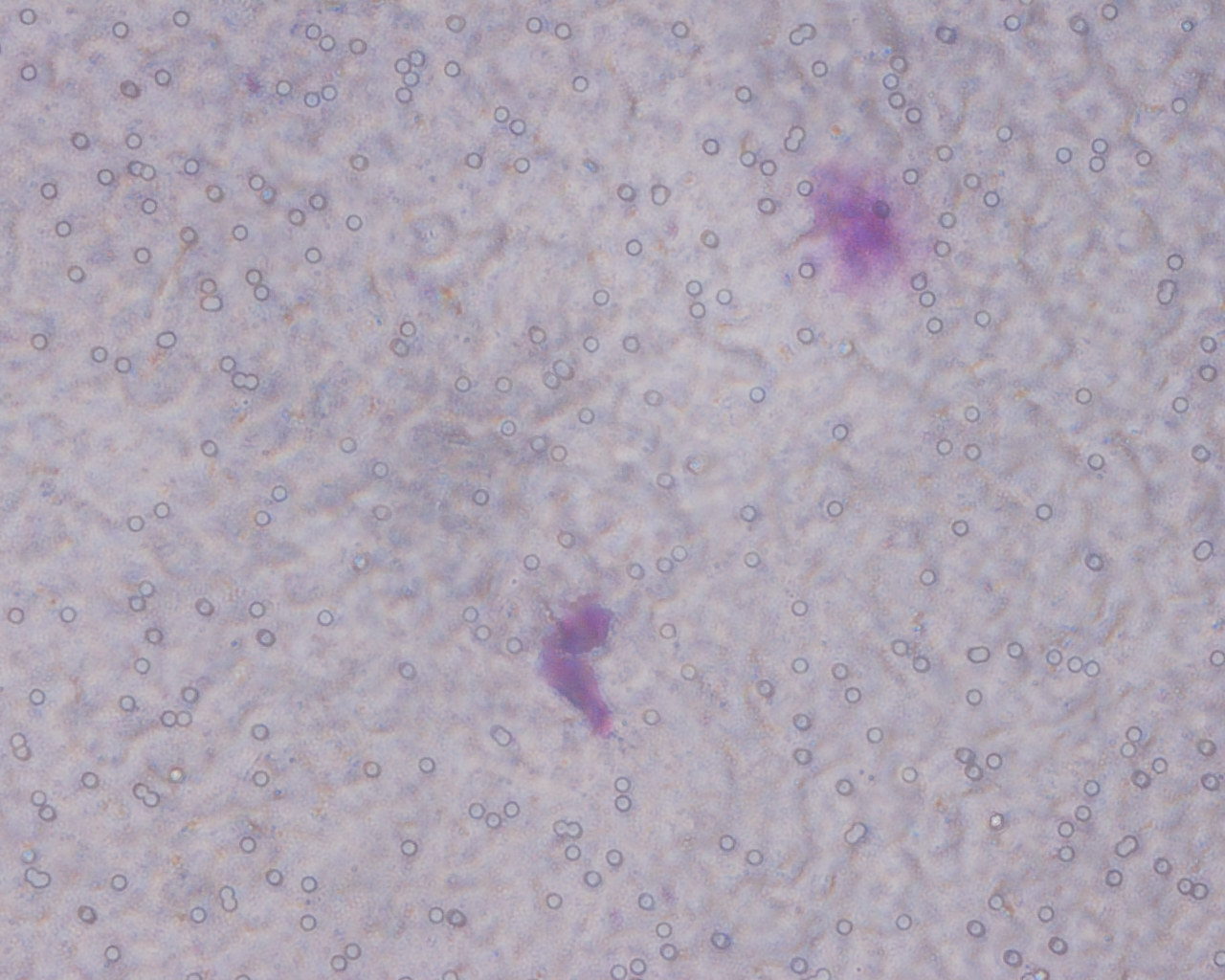

Supplement: S4 File — (ZIP) [file pgen.1010332.s004.zip › S4 File/S18 Migration NKILA.tif]

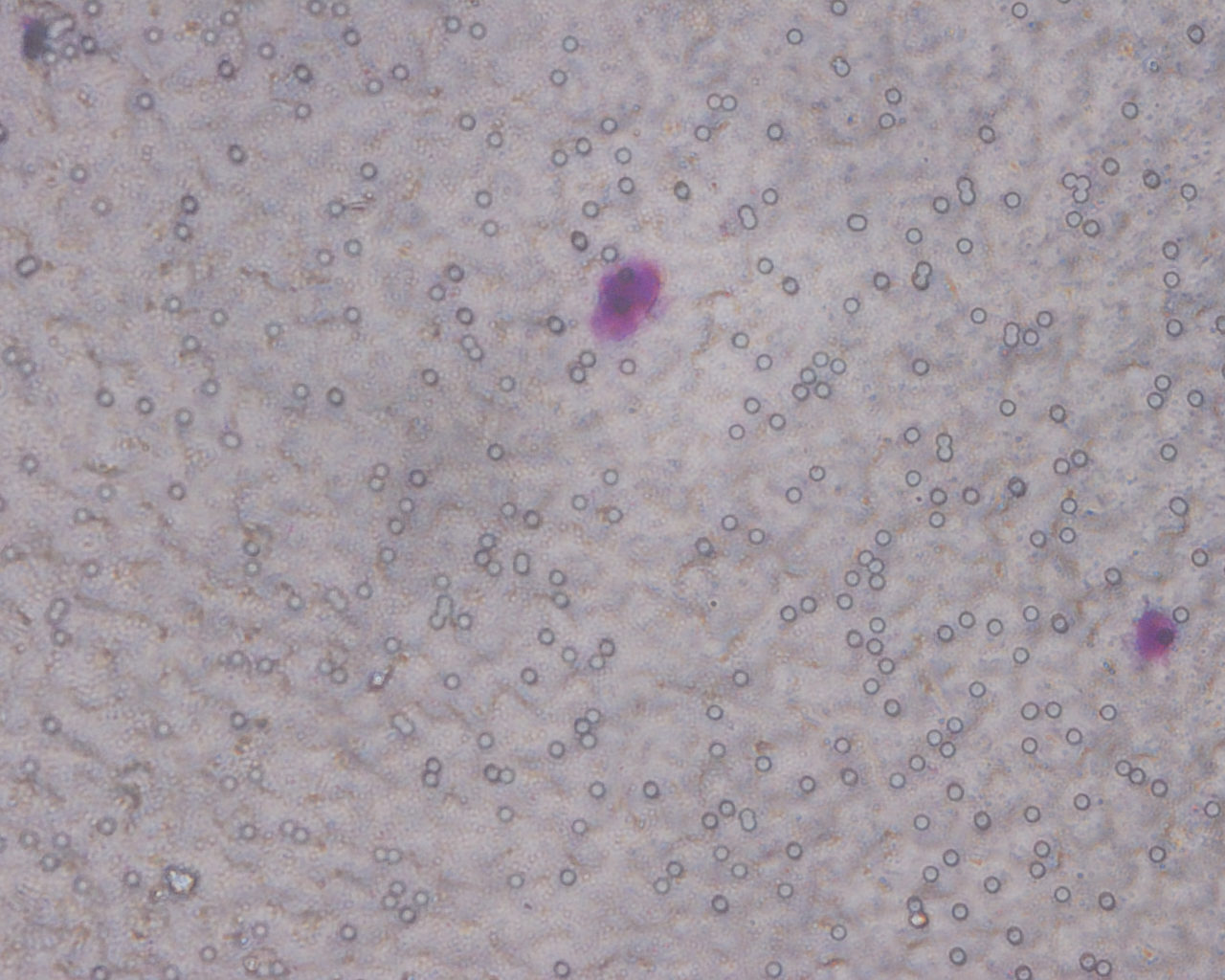

Supplement: S4 File — (ZIP) [file pgen.1010332.s004.zip › S4 File/S18 Migration NKILA-1.tif]

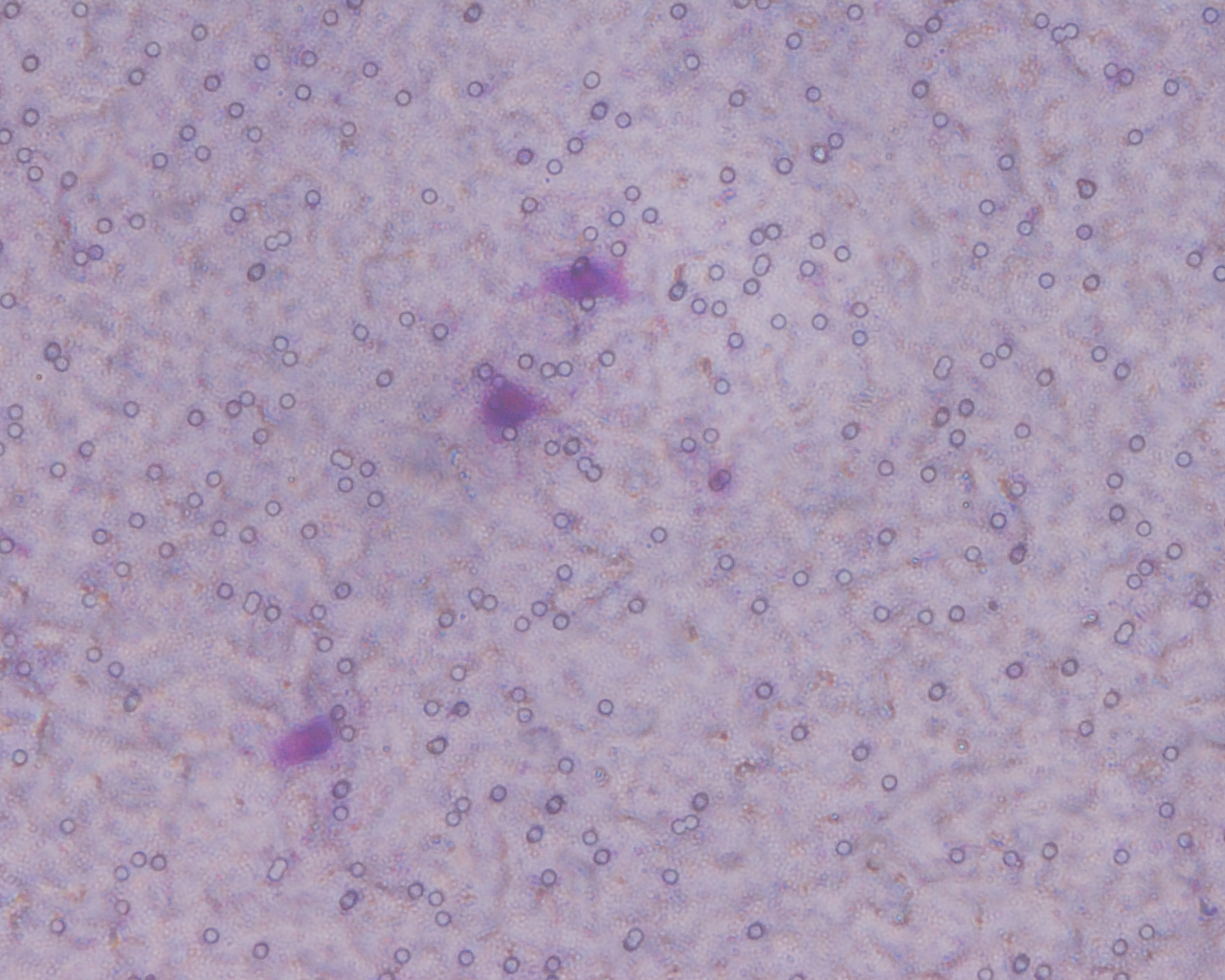

Supplement: S4 File — (ZIP) [file pgen.1010332.s004.zip › S4 File/S18 Migration NKILA-2.tif]

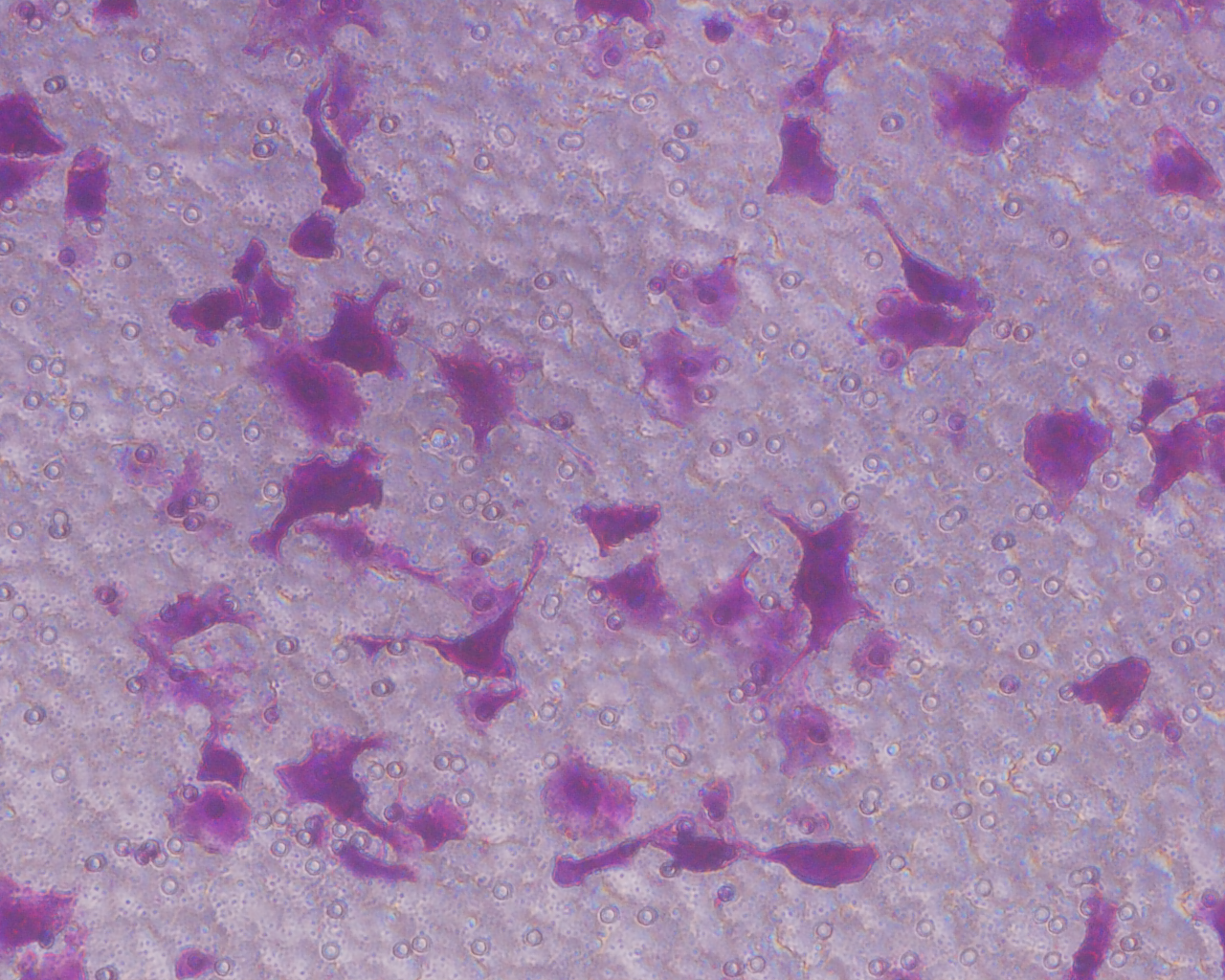

Supplement: S4 File — (ZIP) [file pgen.1010332.s004.zip › S4 File/S18 Migration vec.tif]

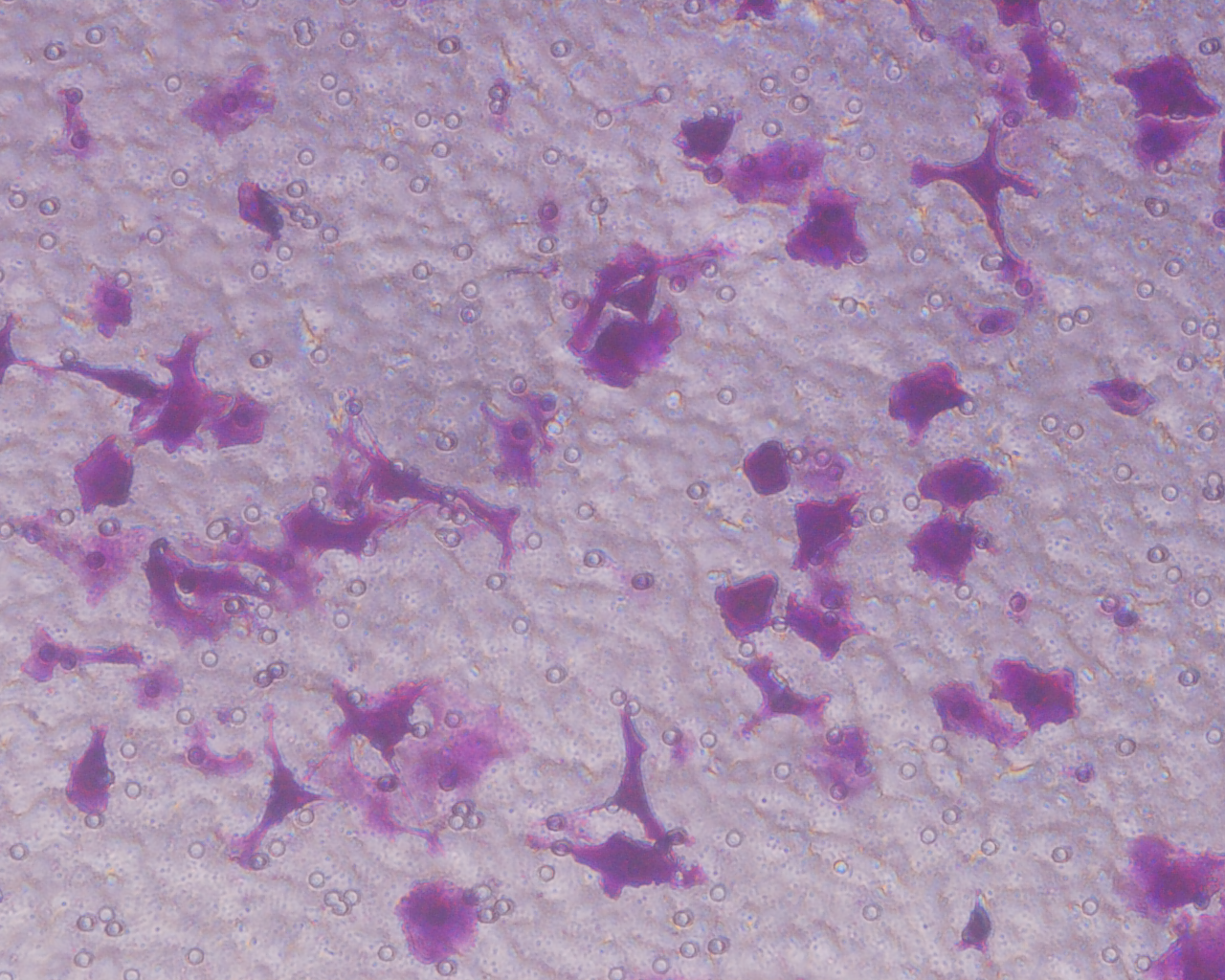

Supplement: S4 File — (ZIP) [file pgen.1010332.s004.zip › S4 File/S18 Migration vec-1.tif]

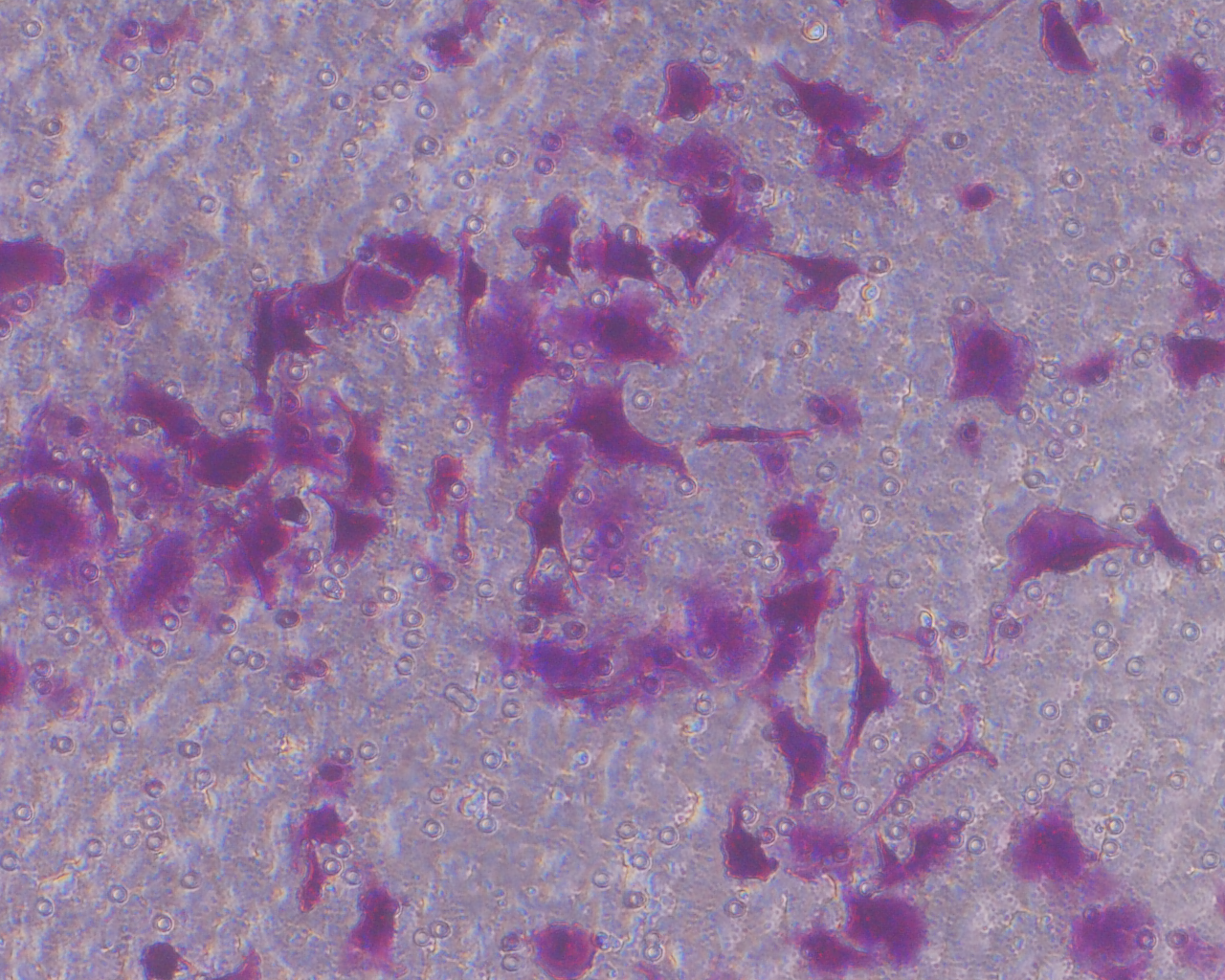

Supplement: S4 File — (ZIP) [file pgen.1010332.s004.zip › S4 File/S18 Migration vec-2.tif]

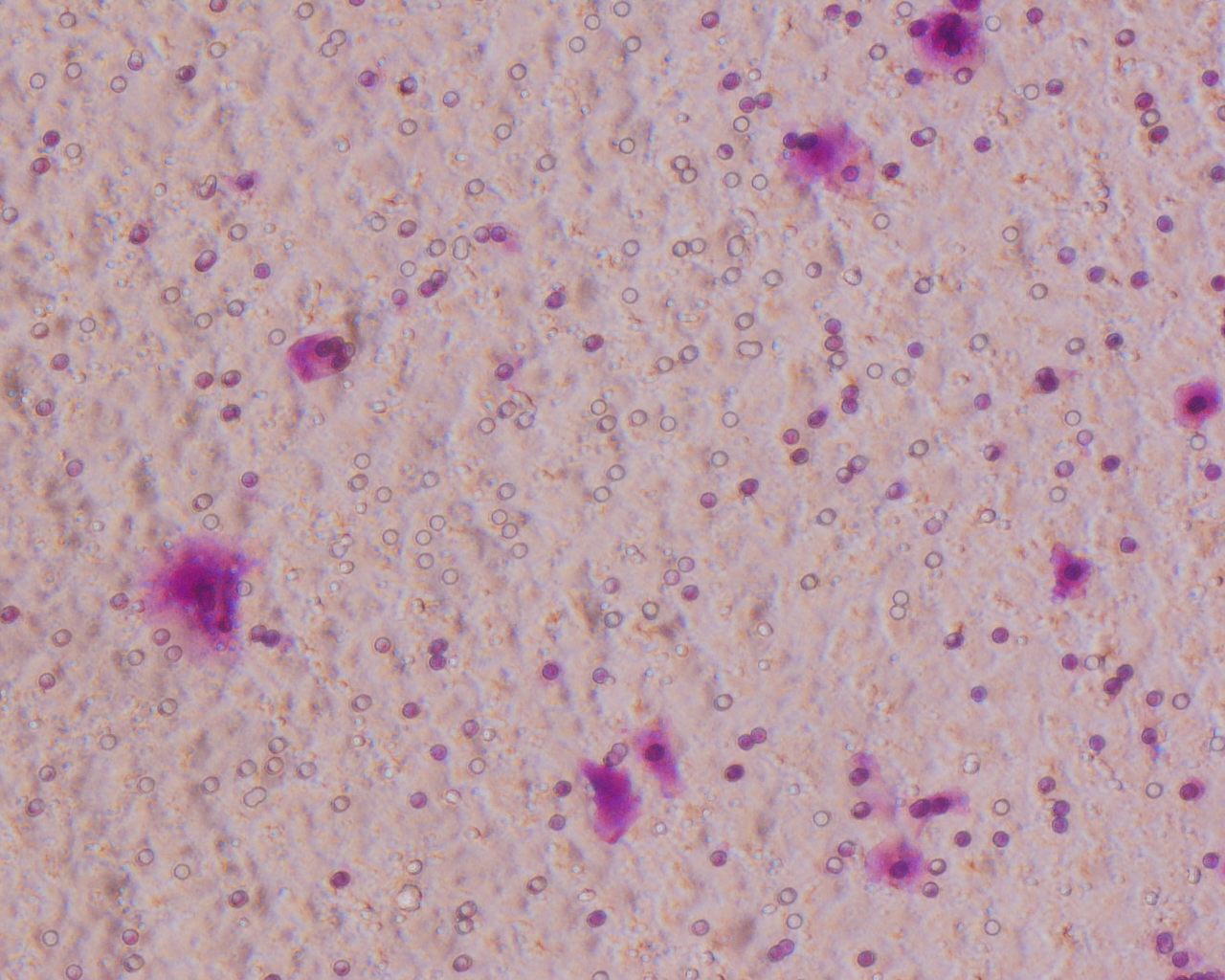

Supplement: S4 File — (ZIP) [file pgen.1010332.s004.zip › S4 File/S26 Invasion shGFP.tif]

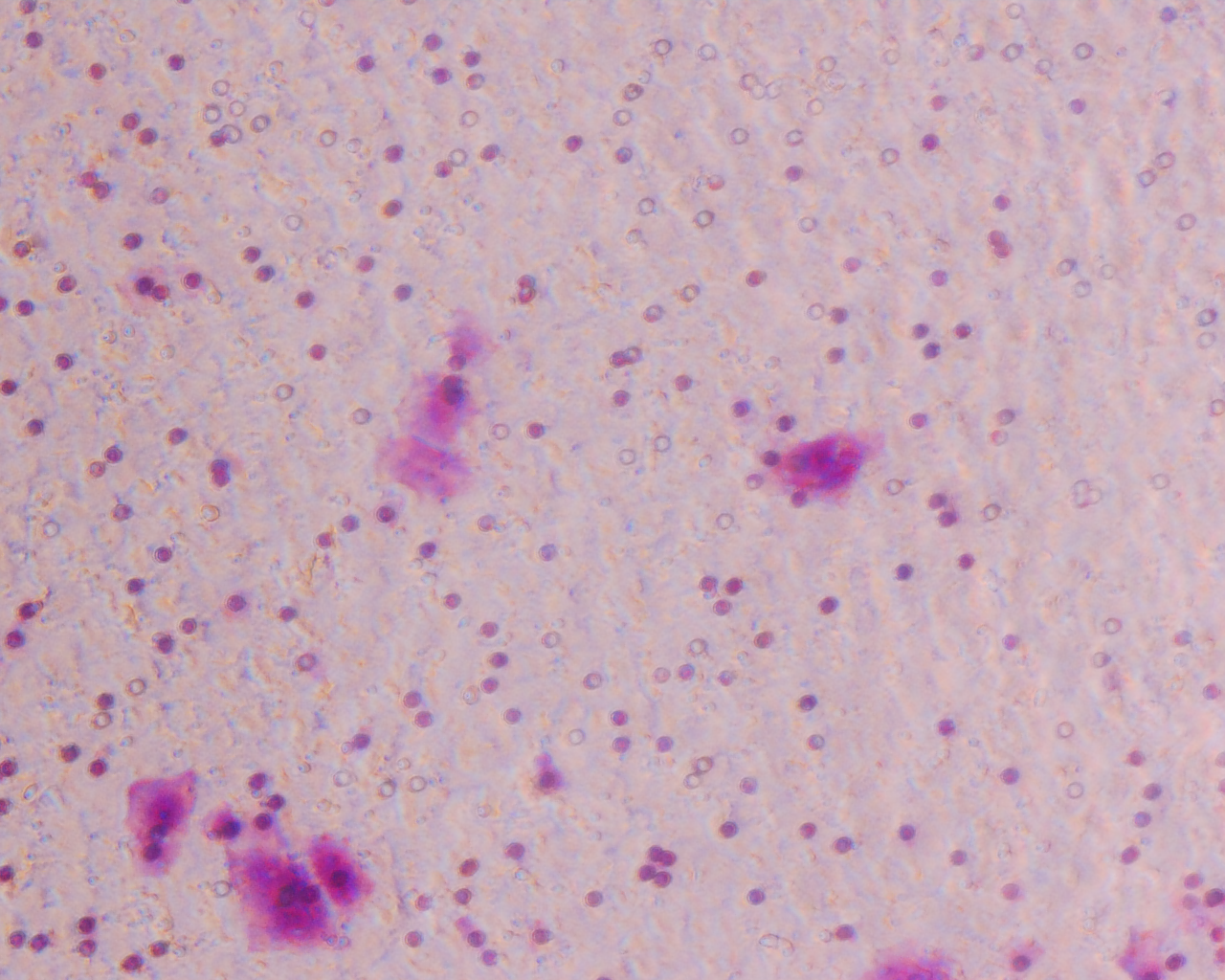

Supplement: S4 File — (ZIP) [file pgen.1010332.s004.zip › S4 File/S26 Invasion shGFP-1.tif]

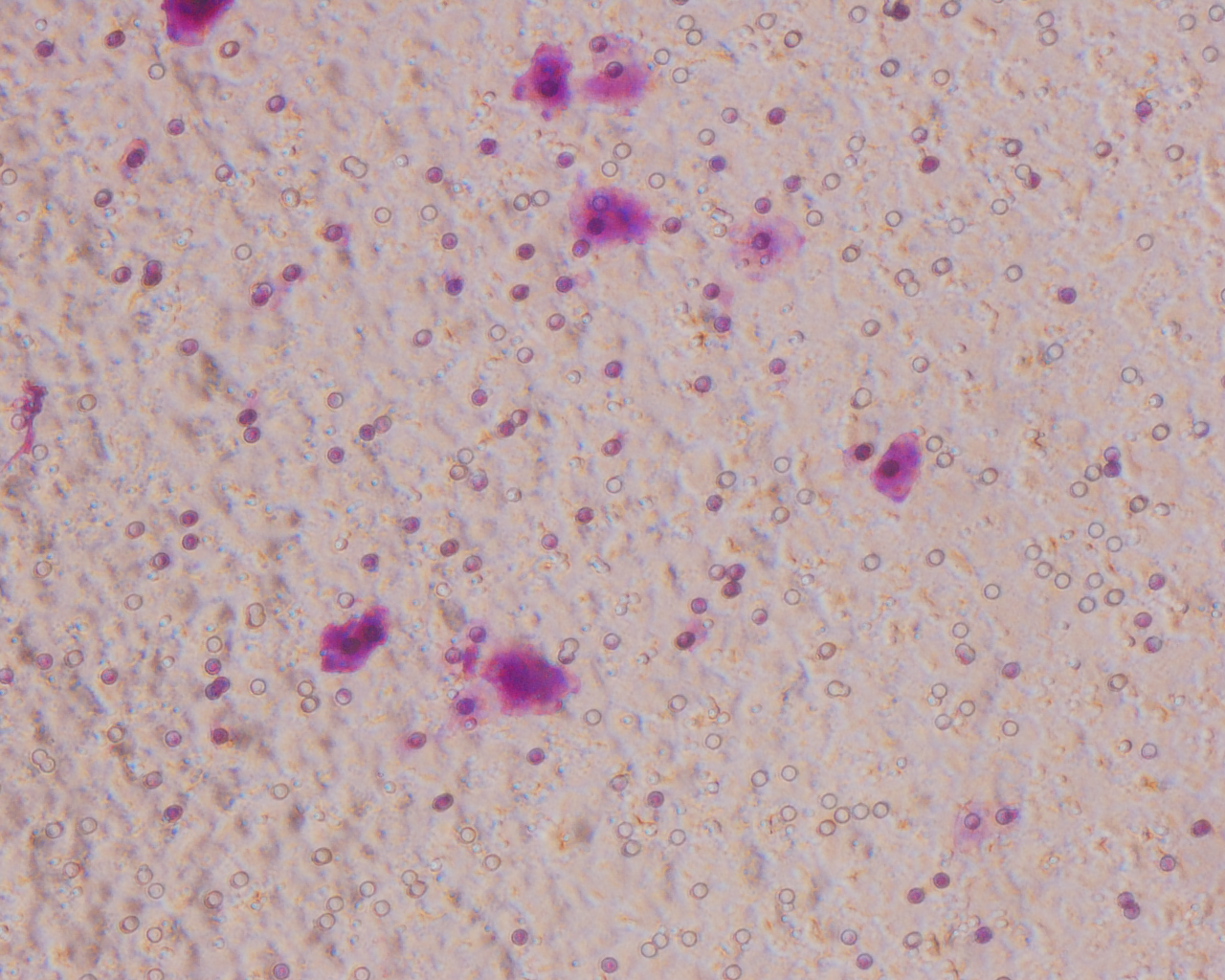

Supplement: S4 File — (ZIP) [file pgen.1010332.s004.zip › S4 File/S26 Invasion shGFP-2.tif]

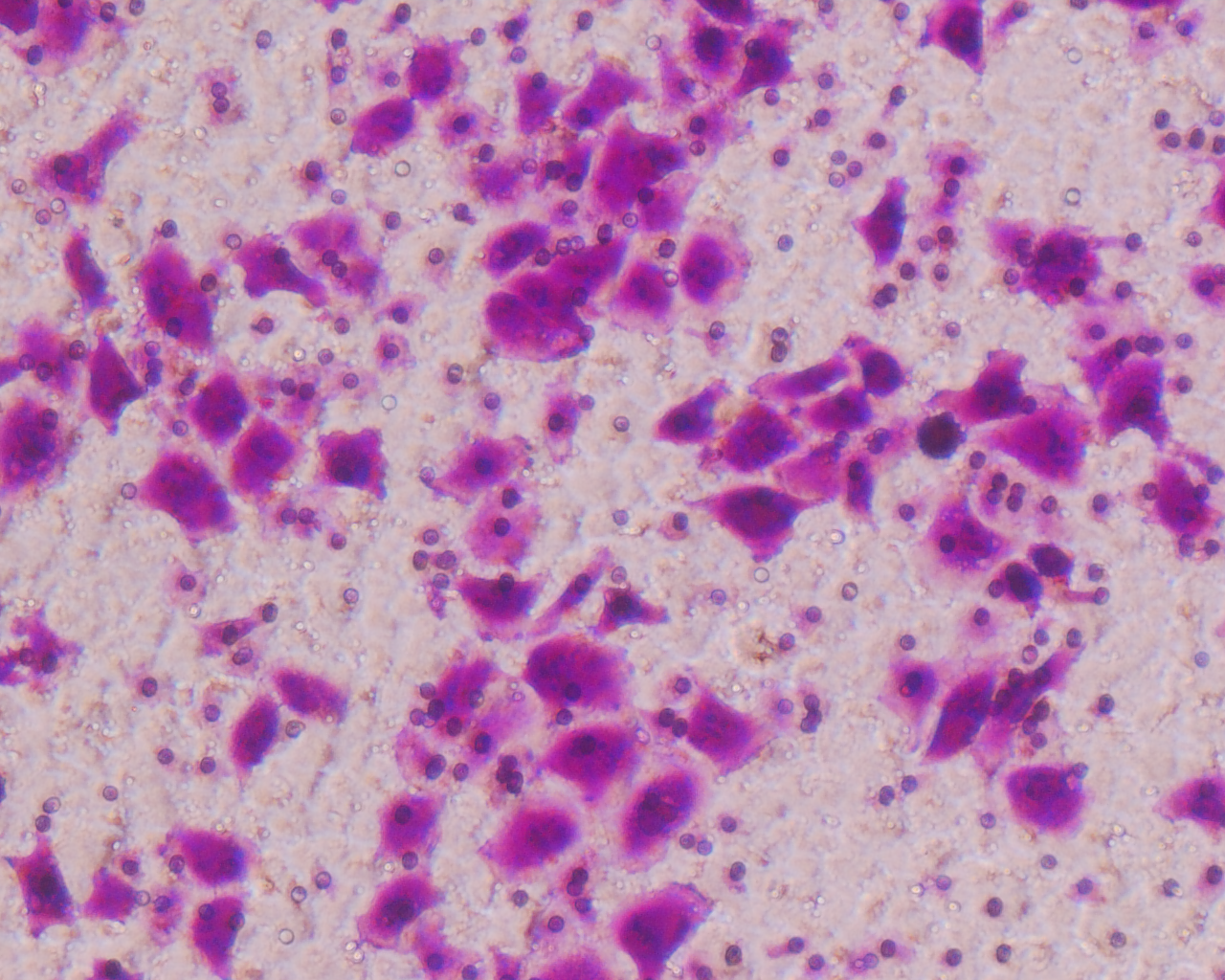

Supplement: S4 File — (ZIP) [file pgen.1010332.s004.zip › S4 File/S26 Invasion shNK1.tif]

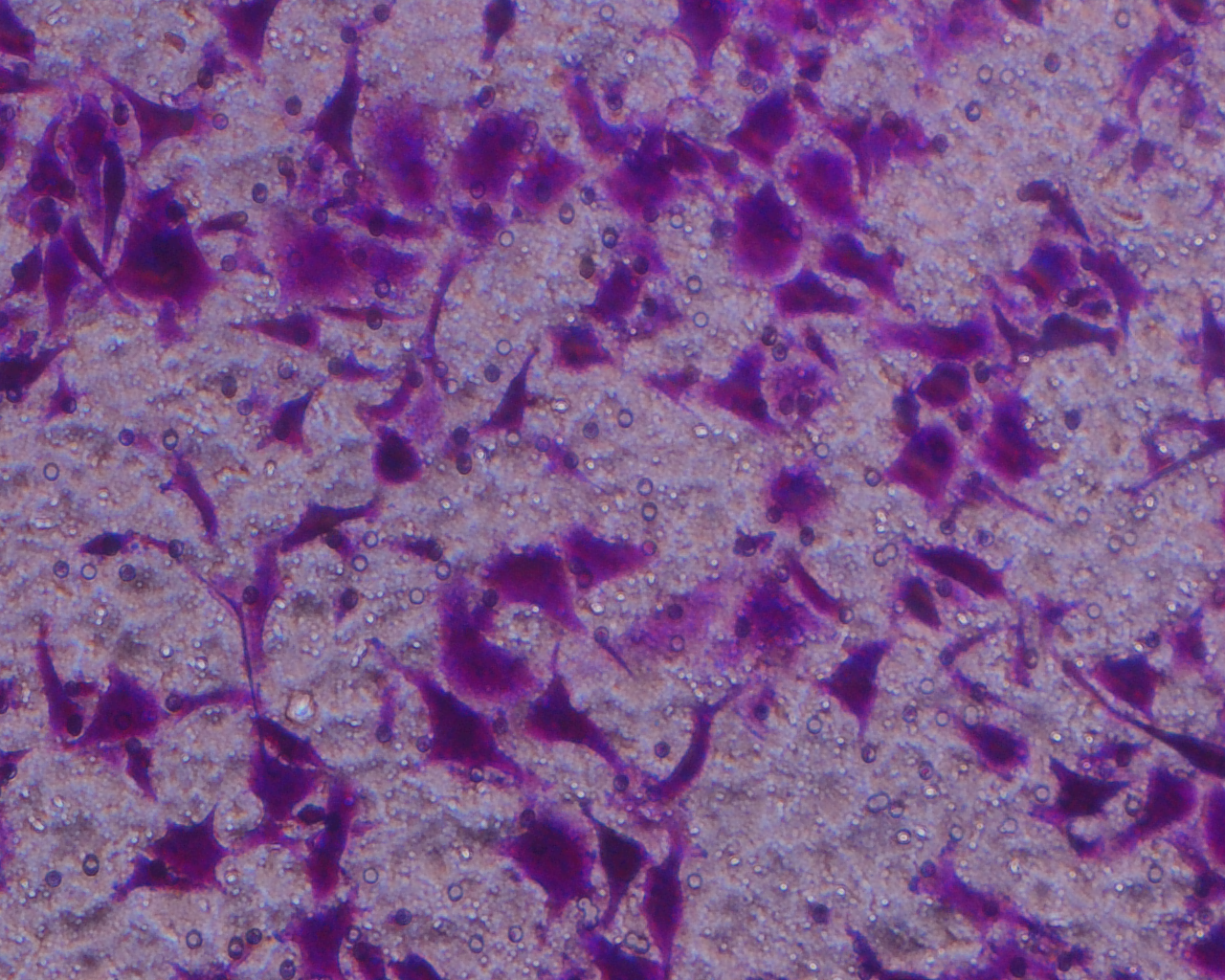

Supplement: S4 File — (ZIP) [file pgen.1010332.s004.zip › S4 File/S26 Invasion shNK1-1.tif]

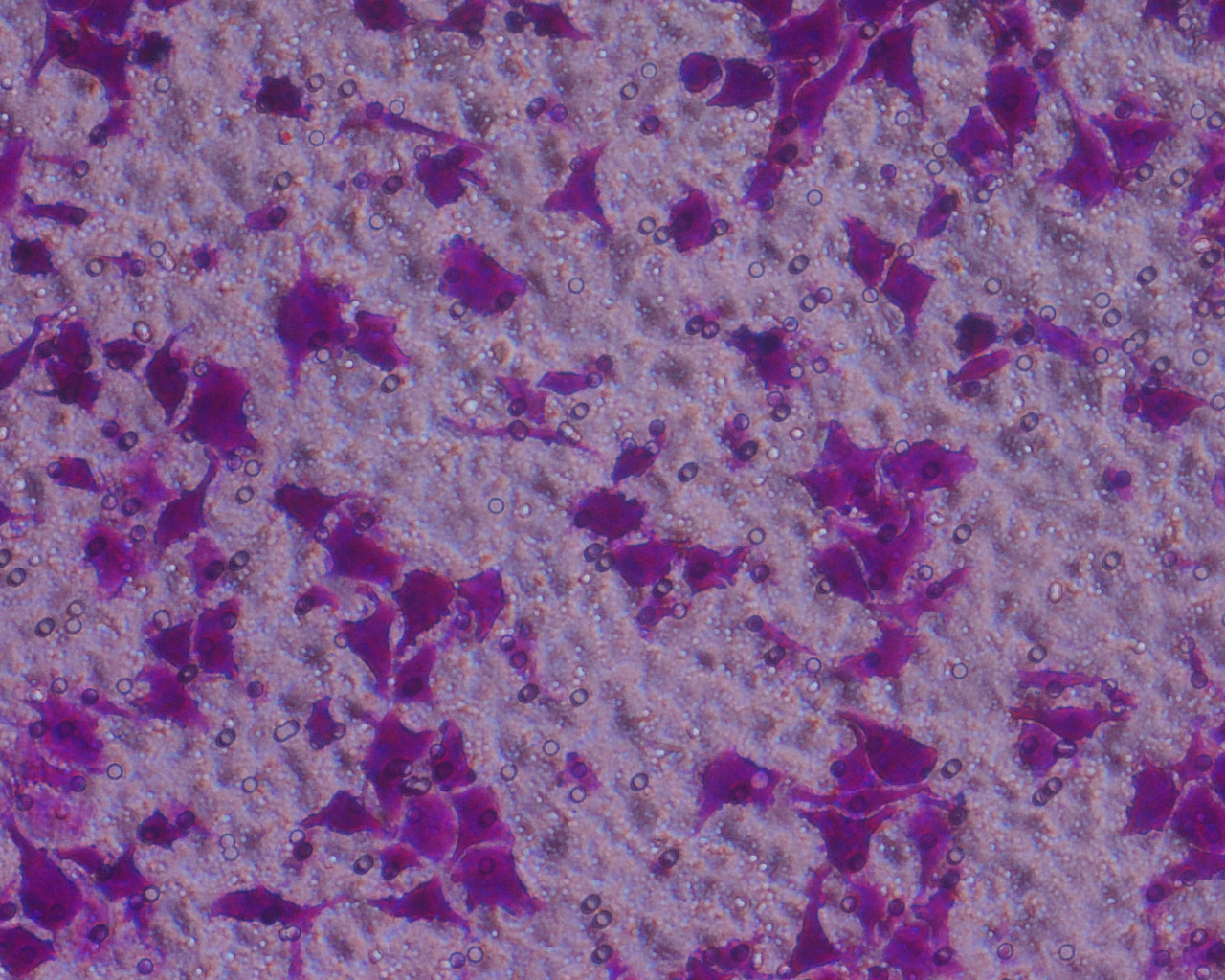

Supplement: S4 File — (ZIP) [file pgen.1010332.s004.zip › S4 File/S26 Invasion shNK1-2.tif]

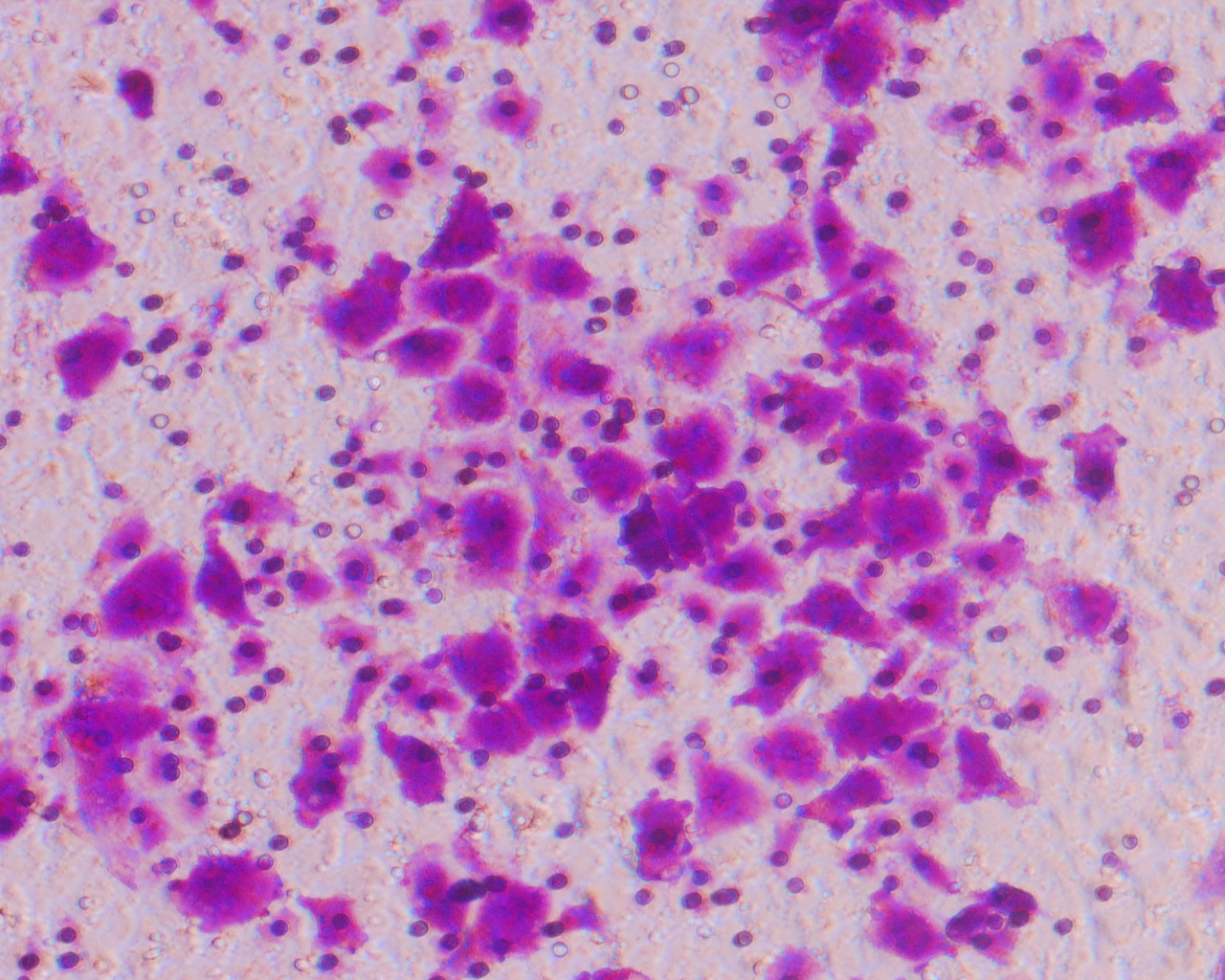

Supplement: S4 File — (ZIP) [file pgen.1010332.s004.zip › S4 File/S26 Invasion shNK2.tif]

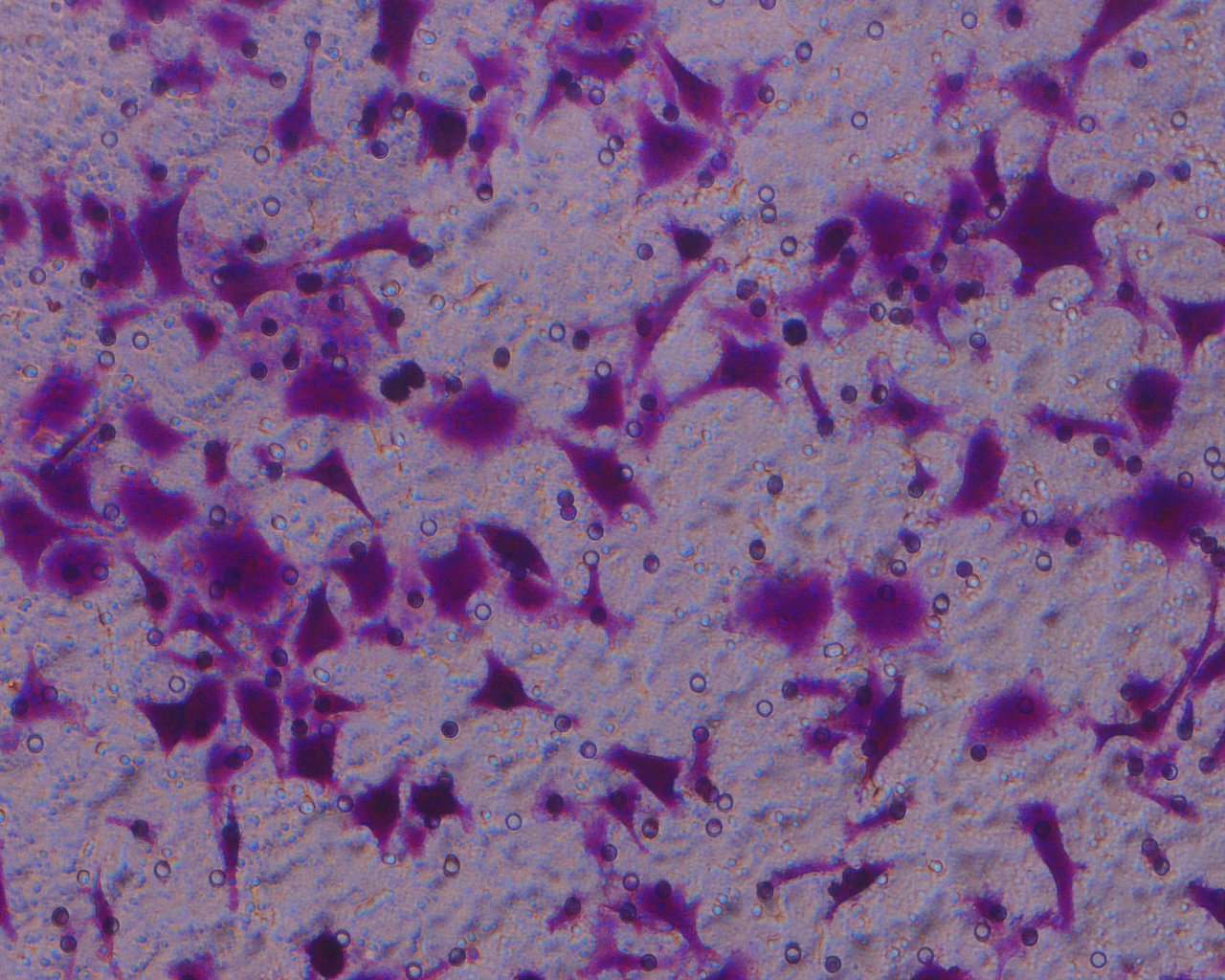

Supplement: S4 File — (ZIP) [file pgen.1010332.s004.zip › S4 File/S26 Invasion shNK2-1.tif]

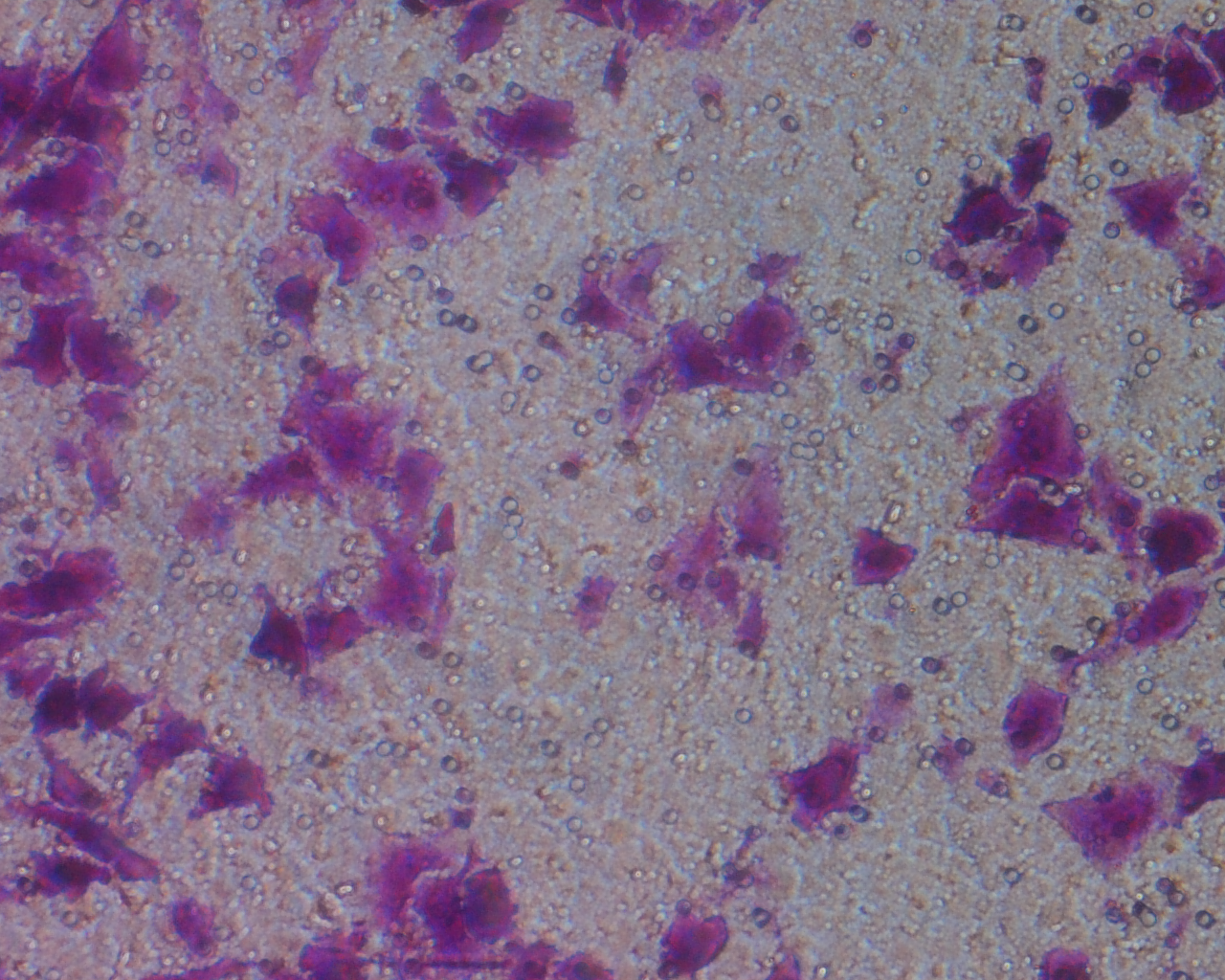

Supplement: S4 File — (ZIP) [file pgen.1010332.s004.zip › S4 File/S26 Invasion shNK2-2.tif]

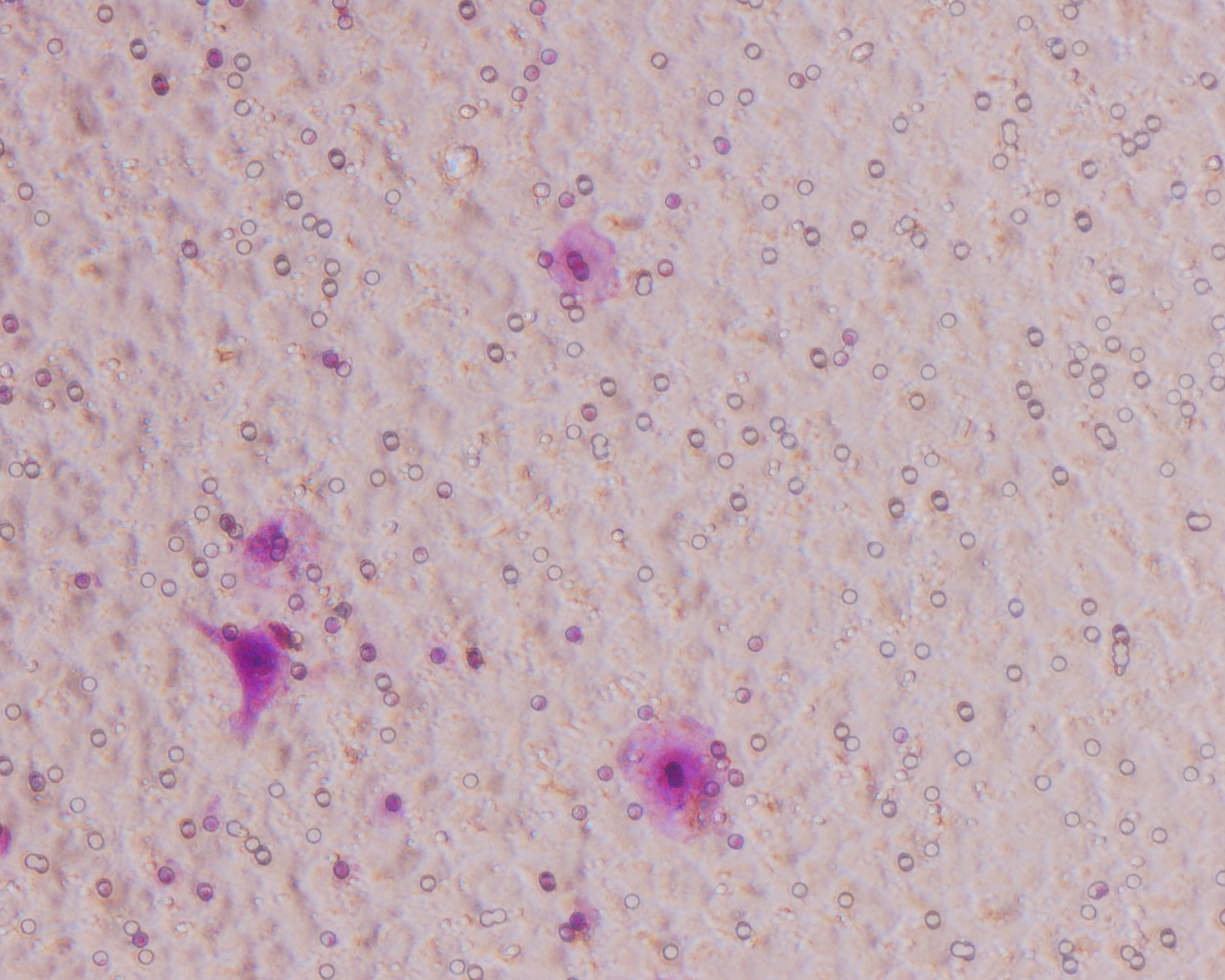

Supplement: S4 File — (ZIP) [file pgen.1010332.s004.zip › S4 File/S26 Invasion shvec.tif]

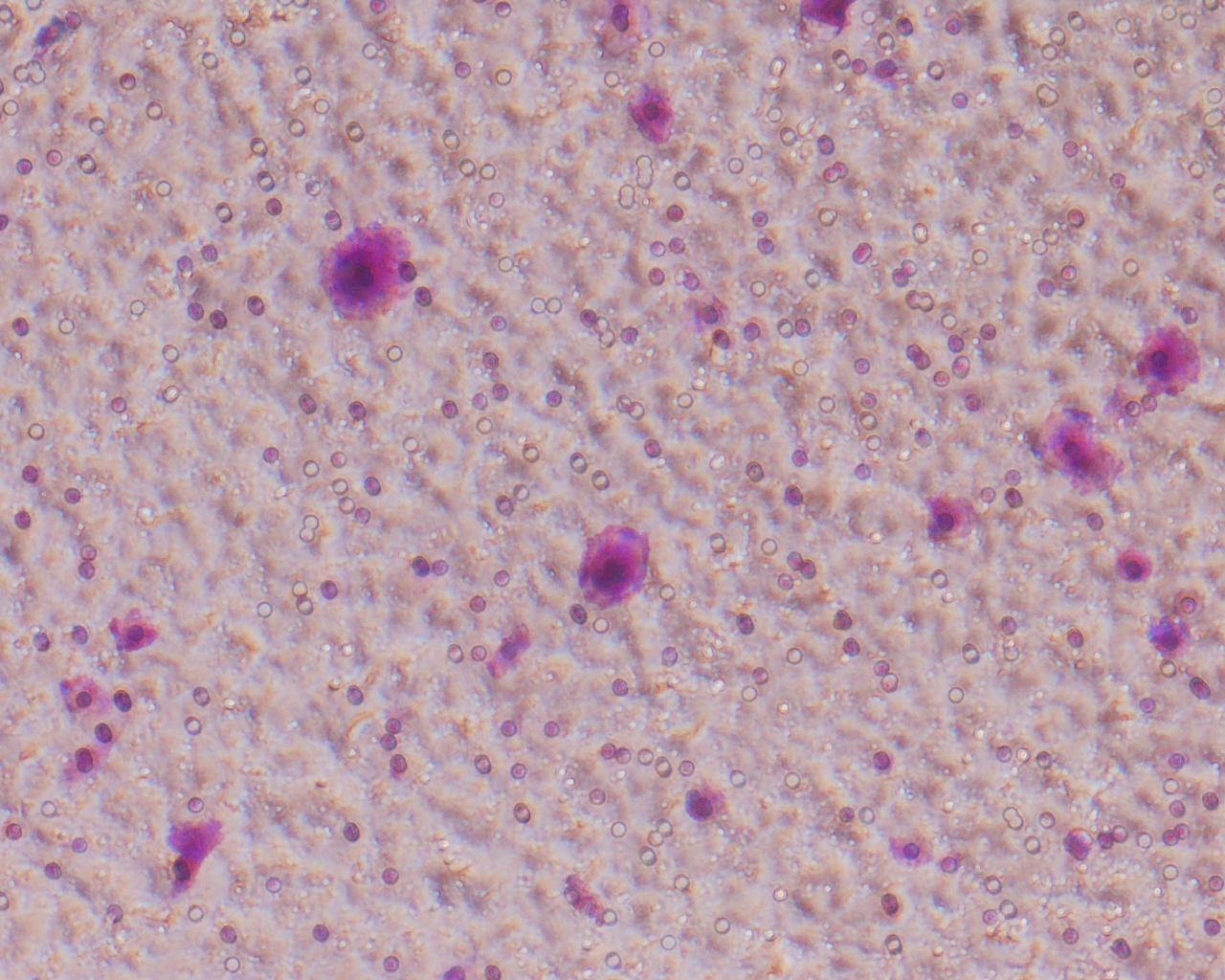

Supplement: S4 File — (ZIP) [file pgen.1010332.s004.zip › S4 File/S26 Invasion shvec-1.tif]

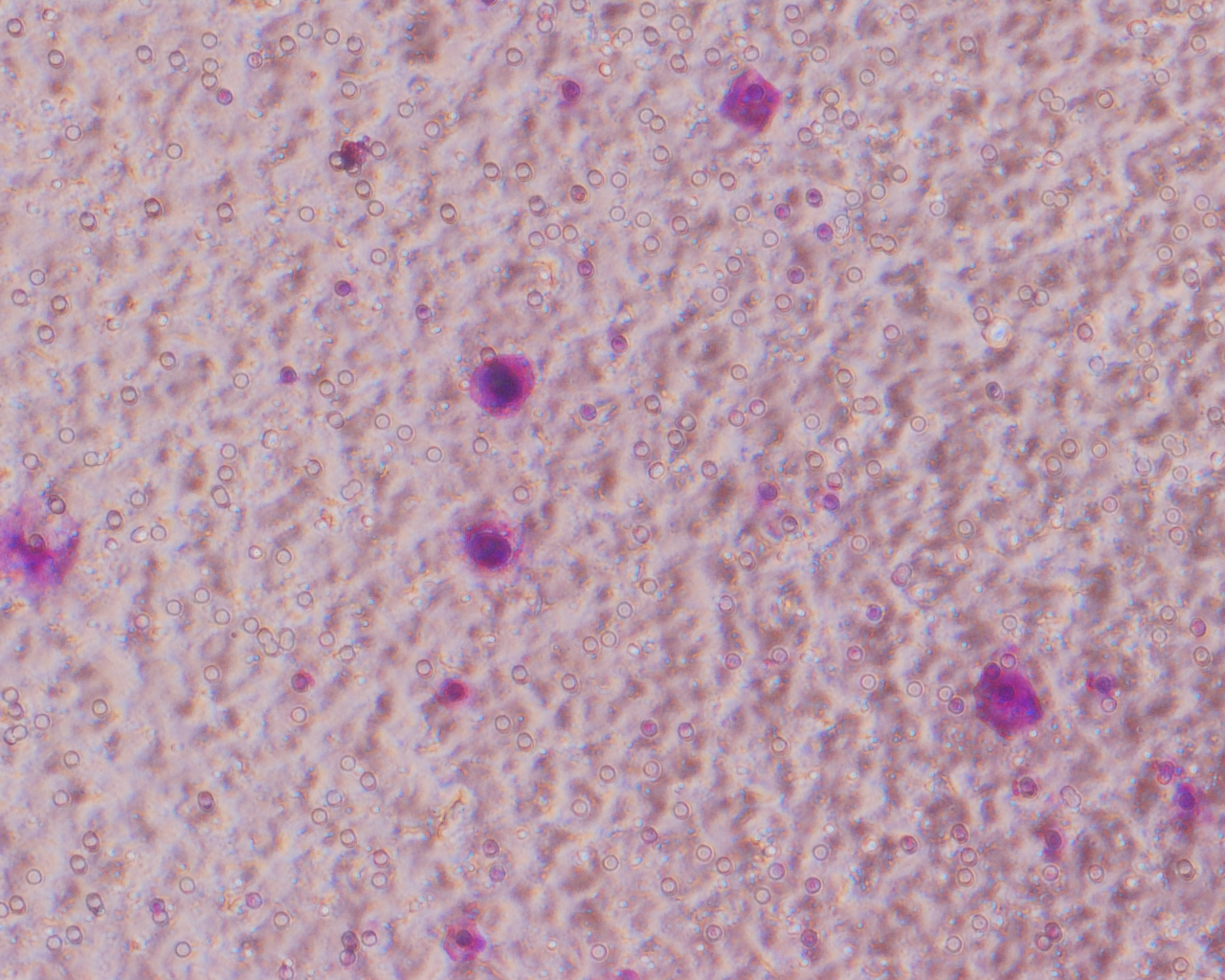

Supplement: S4 File — (ZIP) [file pgen.1010332.s004.zip › S4 File/S26 Invasion shvec-2.tif]

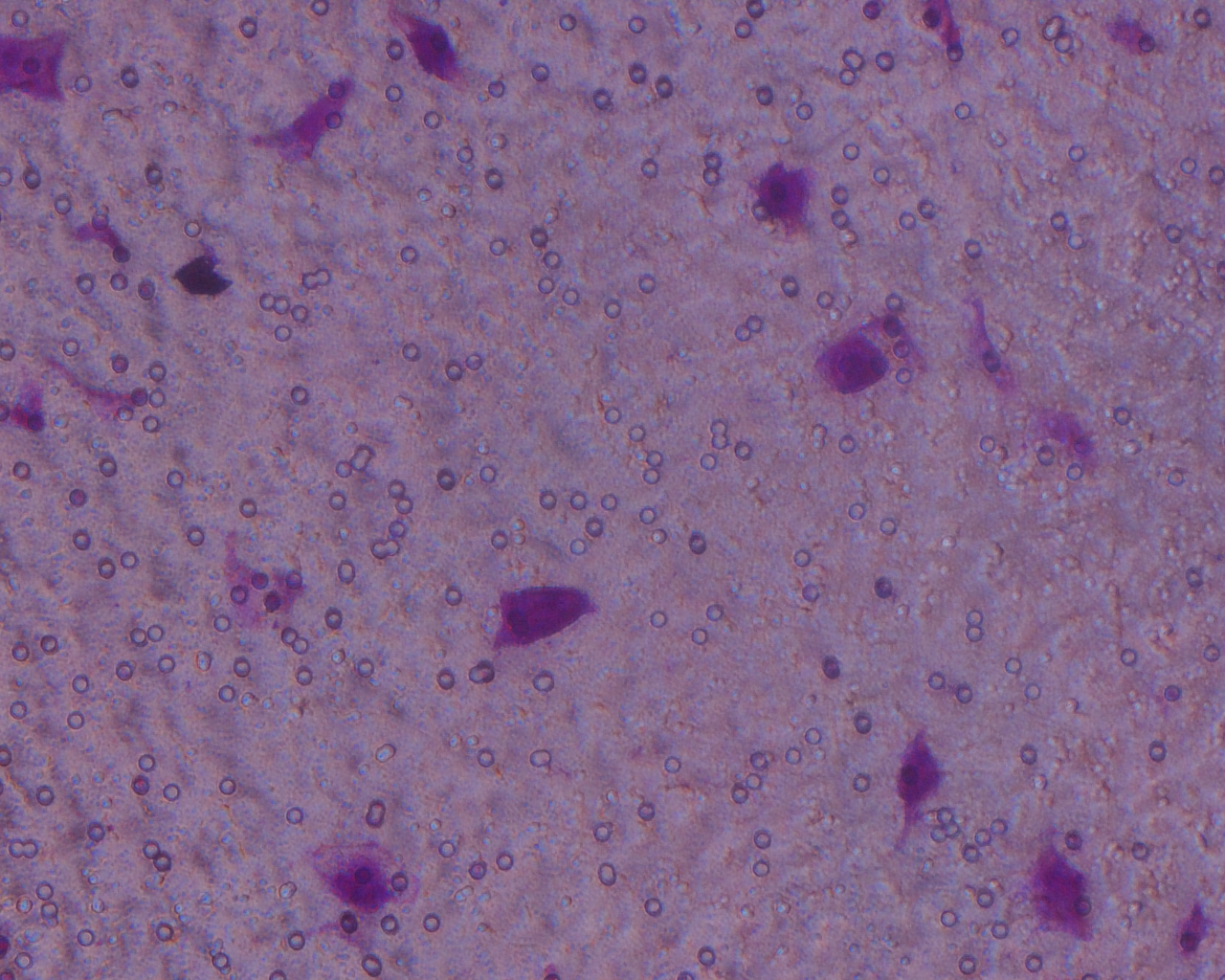

Supplement: S4 File — (ZIP) [file pgen.1010332.s004.zip › S4 File/S26 Migration shGFP.tif]

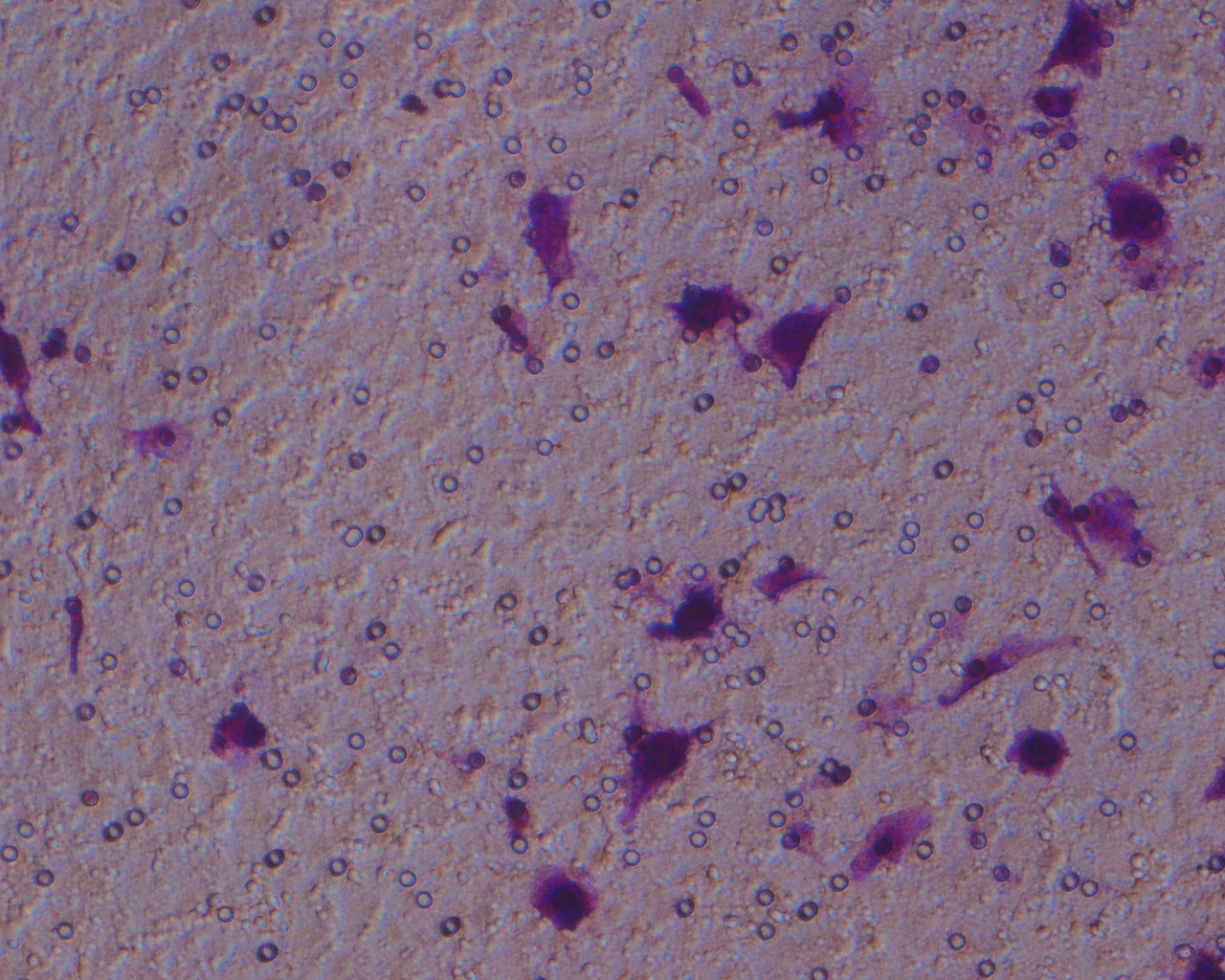

Supplement: S4 File — (ZIP) [file pgen.1010332.s004.zip › S4 File/S26 Migration shGFP-1.tif]

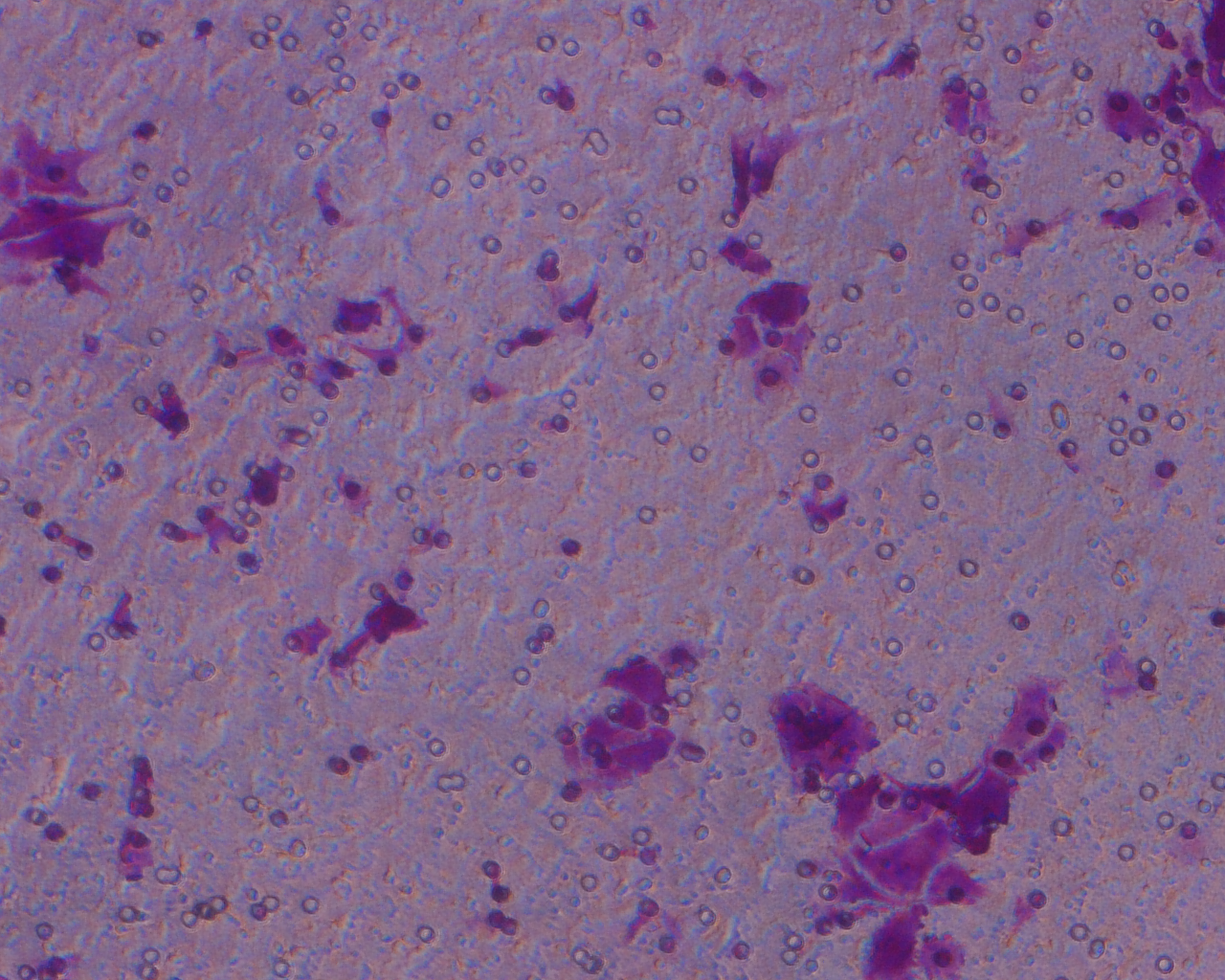

Supplement: S4 File — (ZIP) [file pgen.1010332.s004.zip › S4 File/S26 Migration shGFP-2.tif]

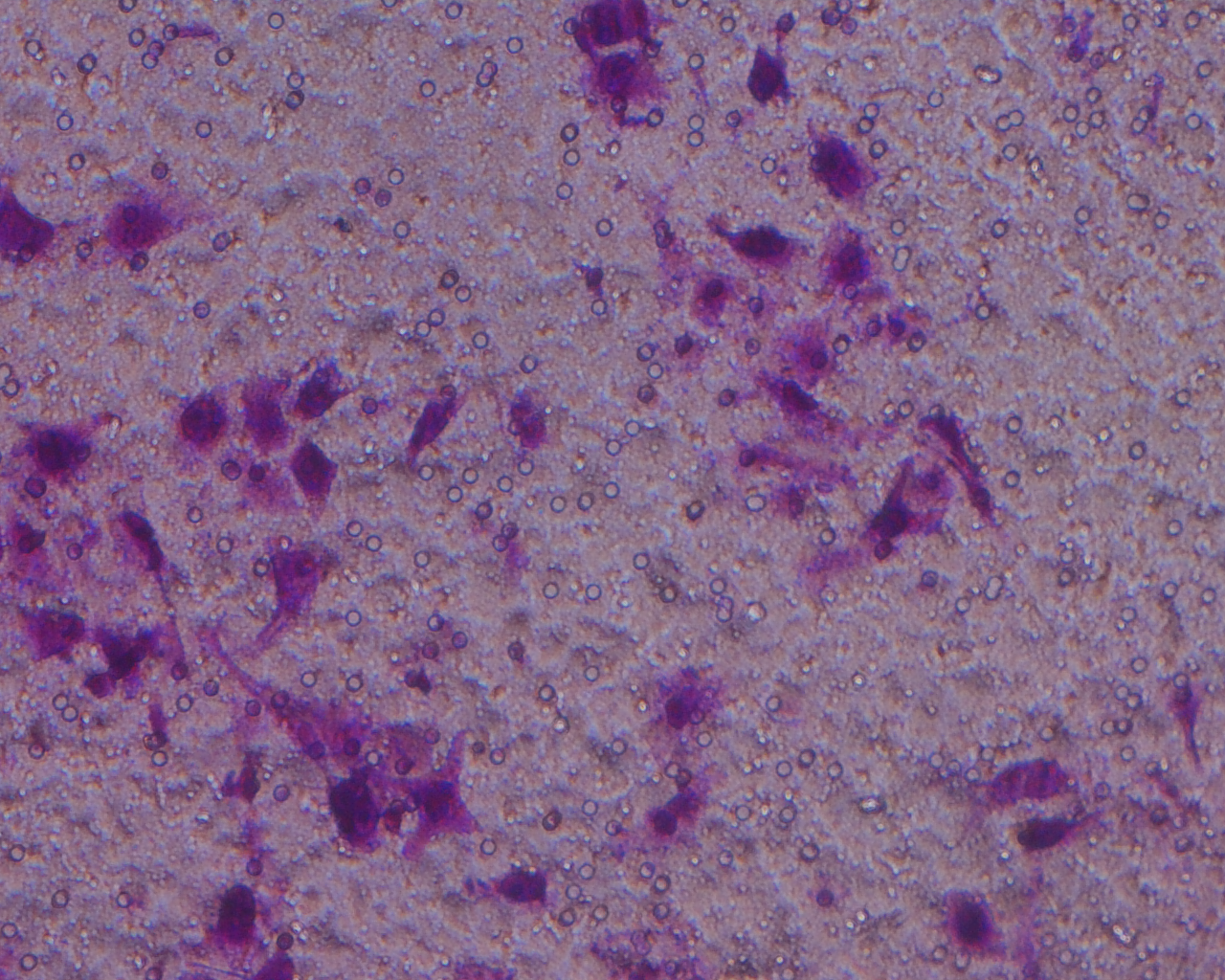

Supplement: S4 File — (ZIP) [file pgen.1010332.s004.zip › S4 File/S26 Migration shNK1.tif]

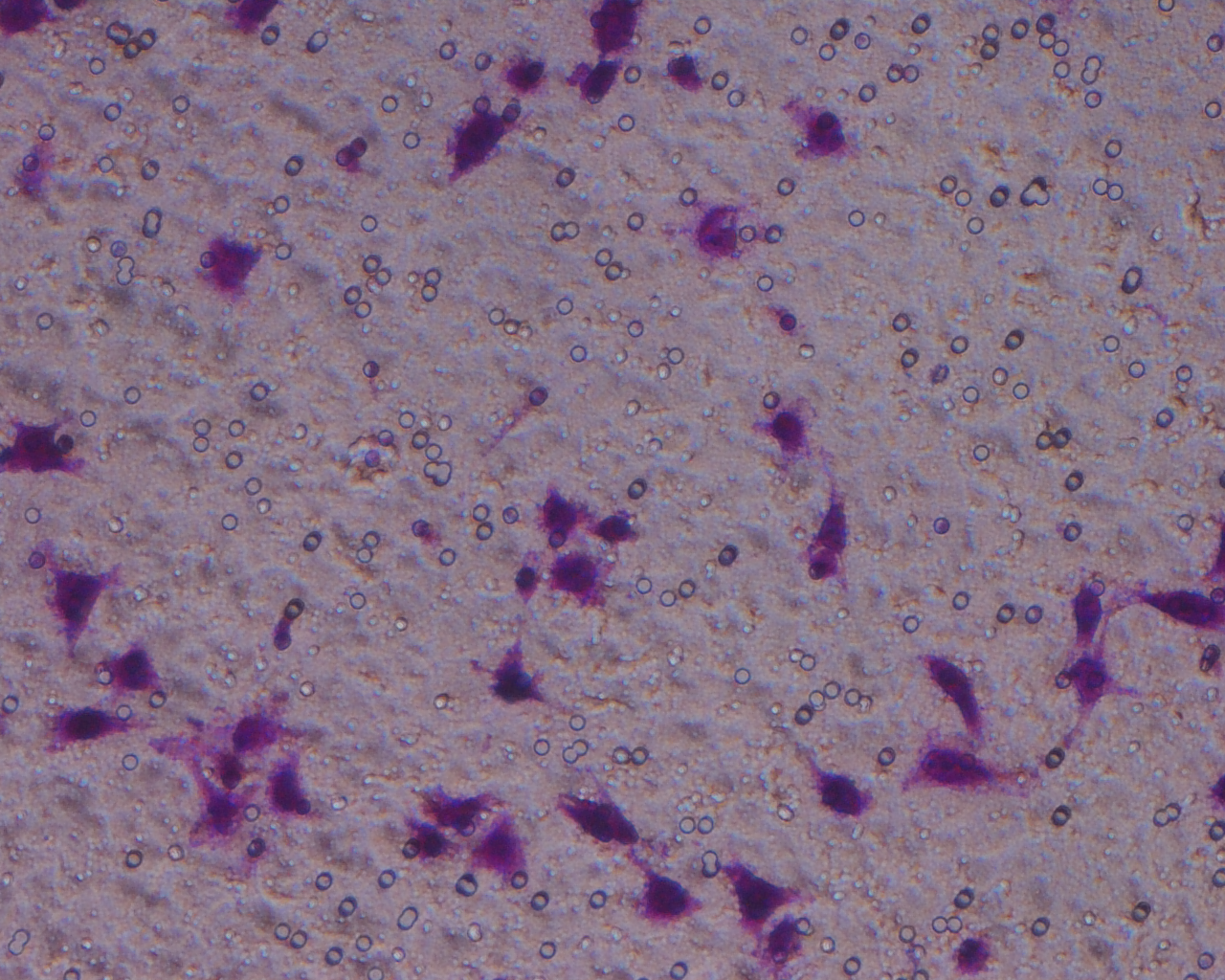

Supplement: S4 File — (ZIP) [file pgen.1010332.s004.zip › S4 File/S26 Migration shNK1-1.tif]

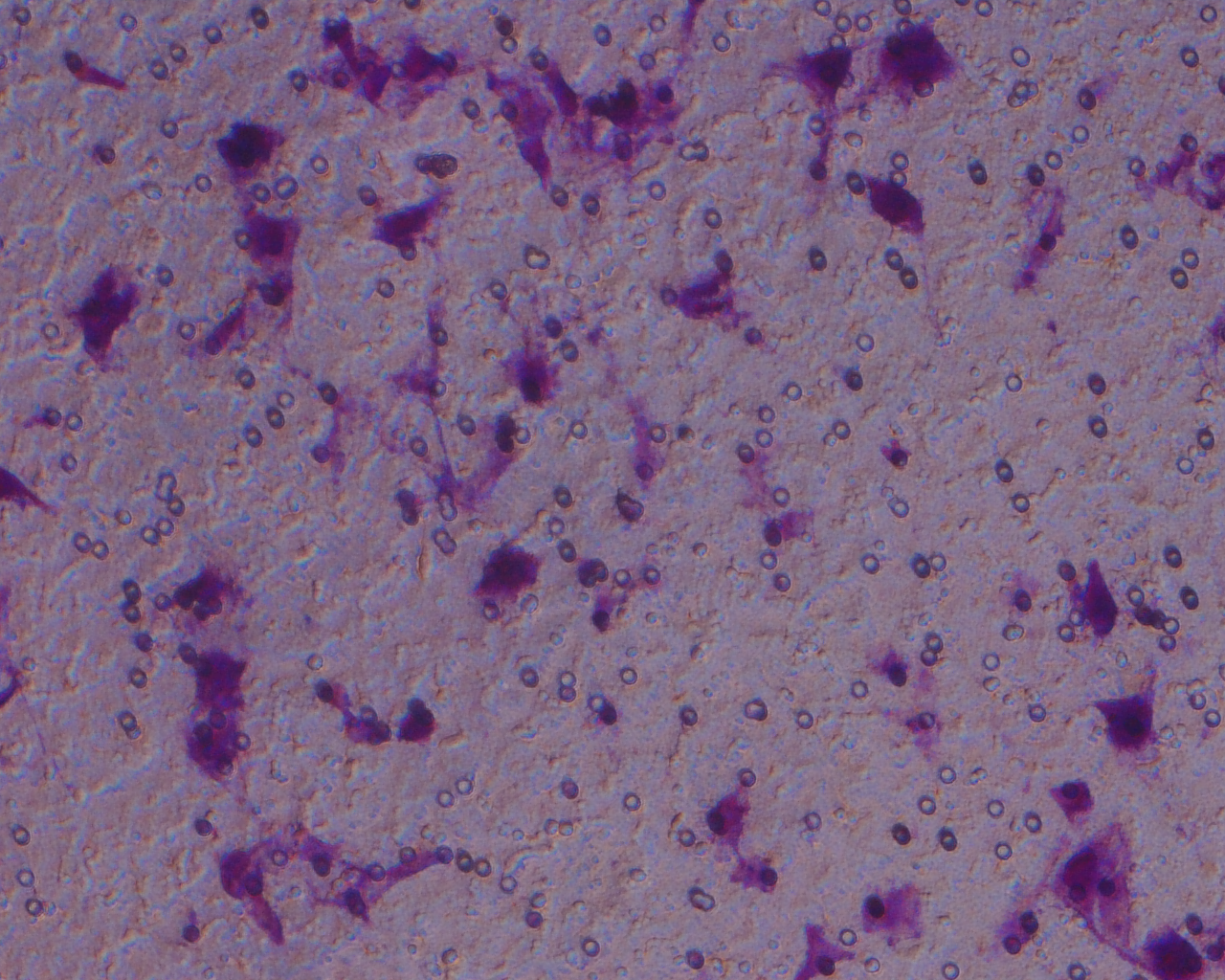

Supplement: S4 File — (ZIP) [file pgen.1010332.s004.zip › S4 File/S26 Migration shNK1-2.tif]

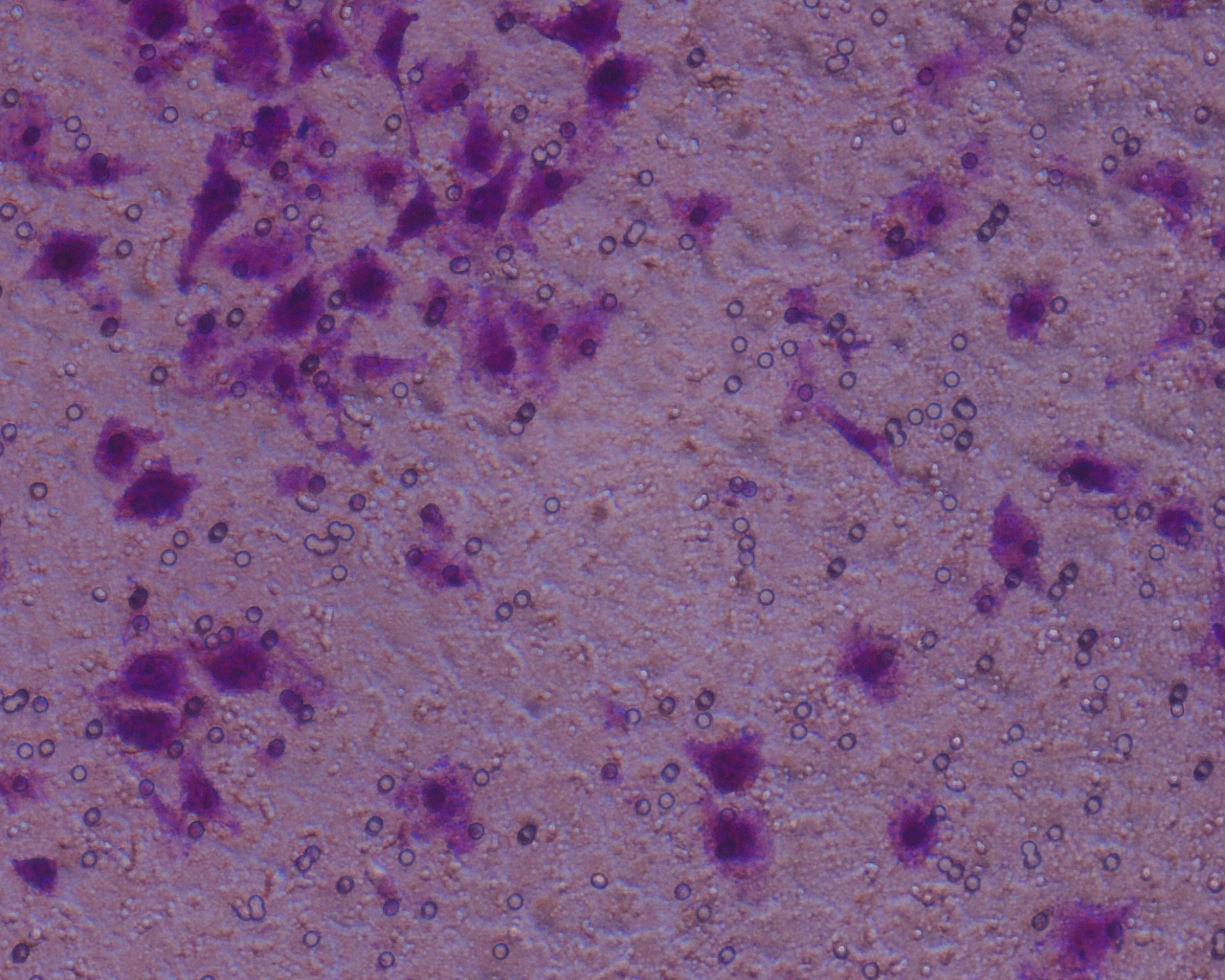

Supplement: S4 File — (ZIP) [file pgen.1010332.s004.zip › S4 File/S26 Migration shNK2.tif]

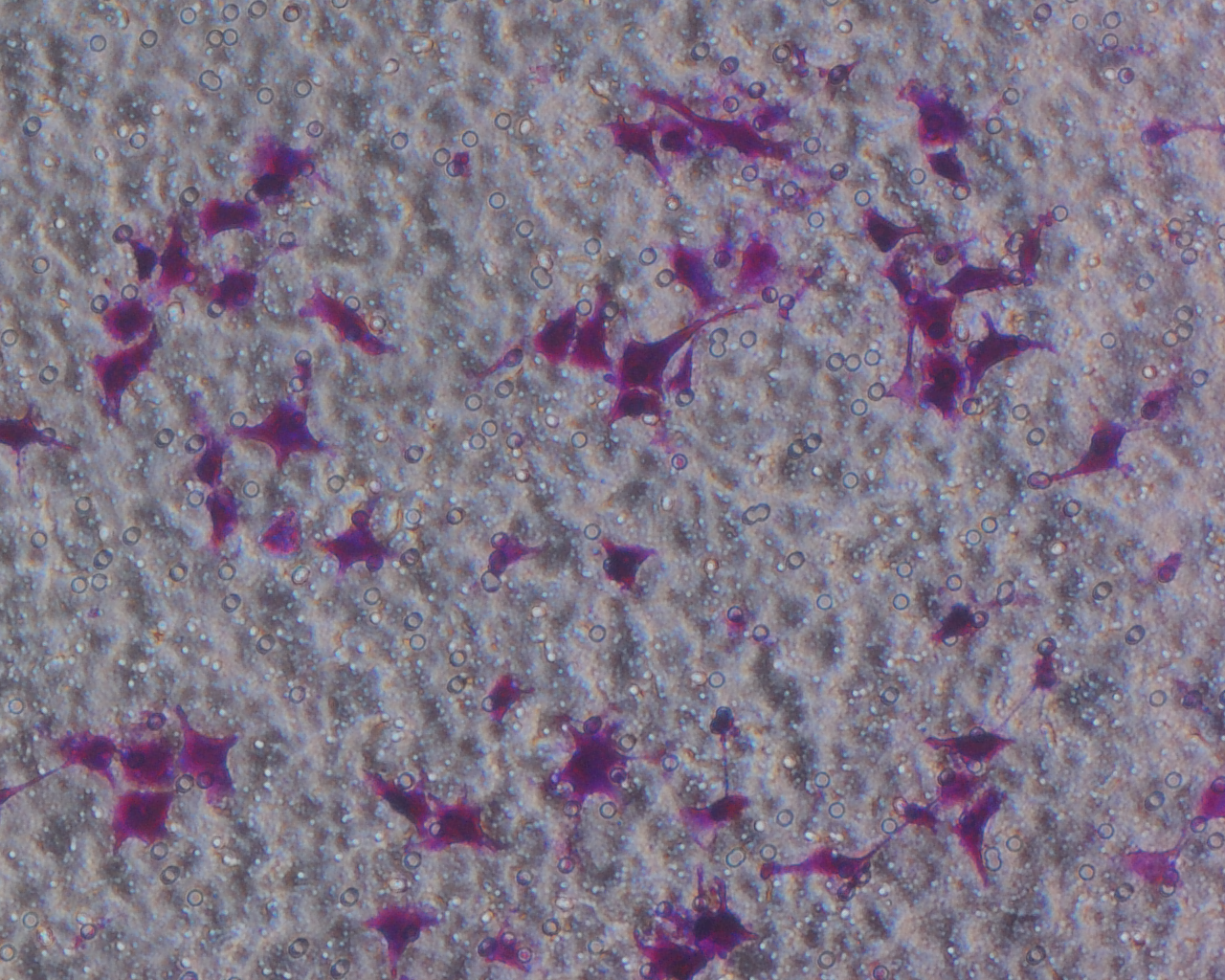

Supplement: S4 File — (ZIP) [file pgen.1010332.s004.zip › S4 File/S26 Migration shNK2-1.tif]

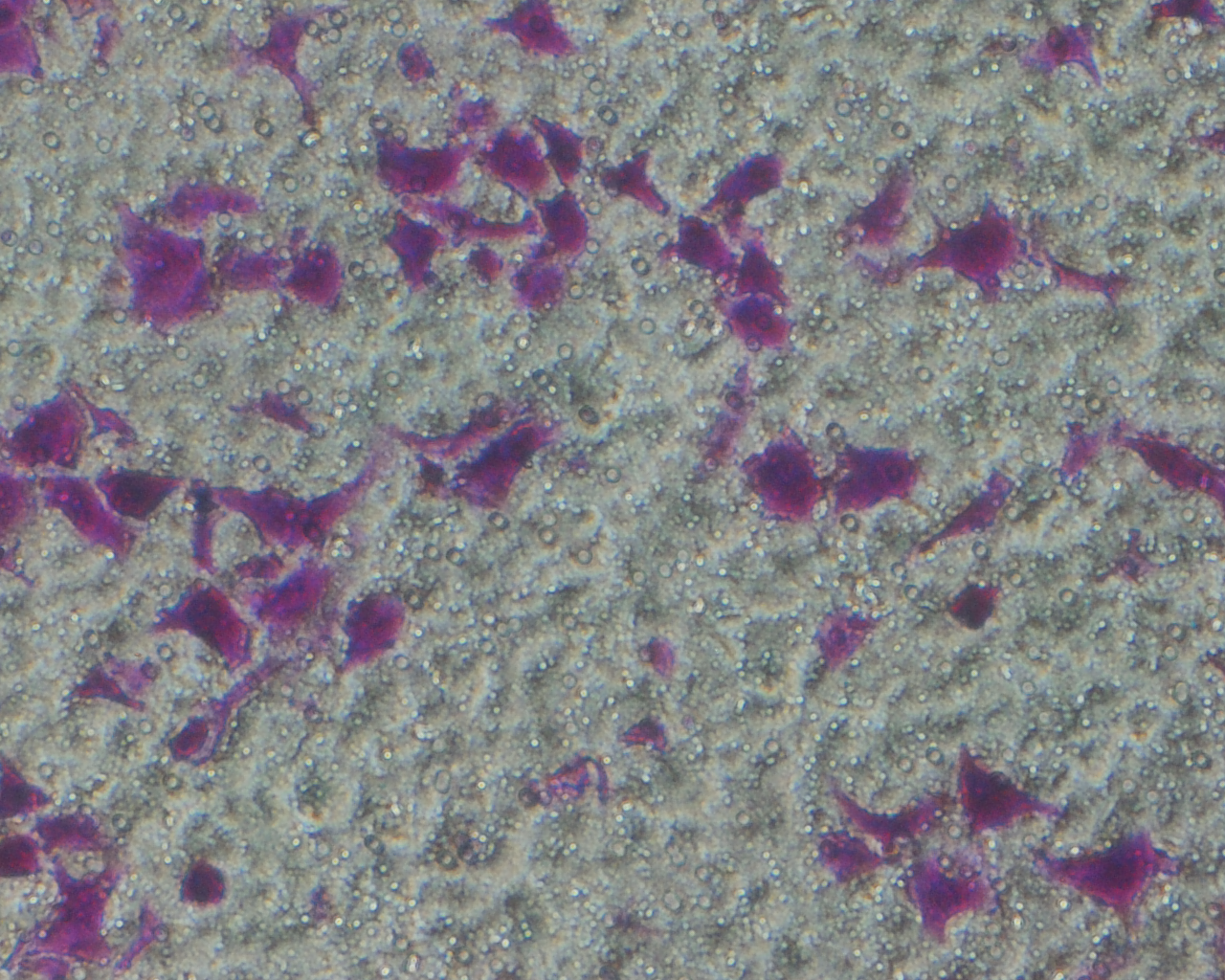

Supplement: S4 File — (ZIP) [file pgen.1010332.s004.zip › S4 File/S26 Migration shNK2-2.tif]

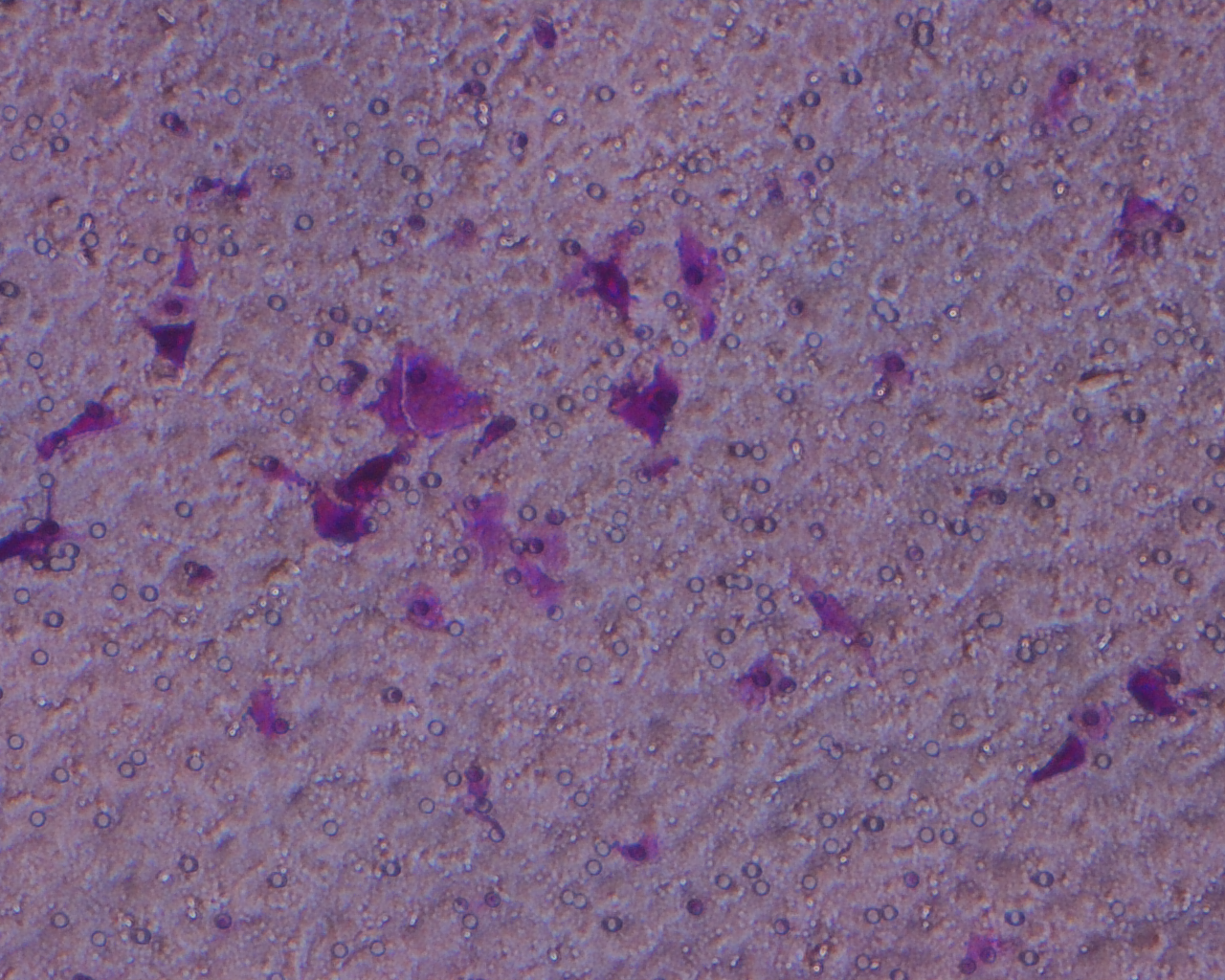

Supplement: S4 File — (ZIP) [file pgen.1010332.s004.zip › S4 File/S26 Migration shvec.tif]

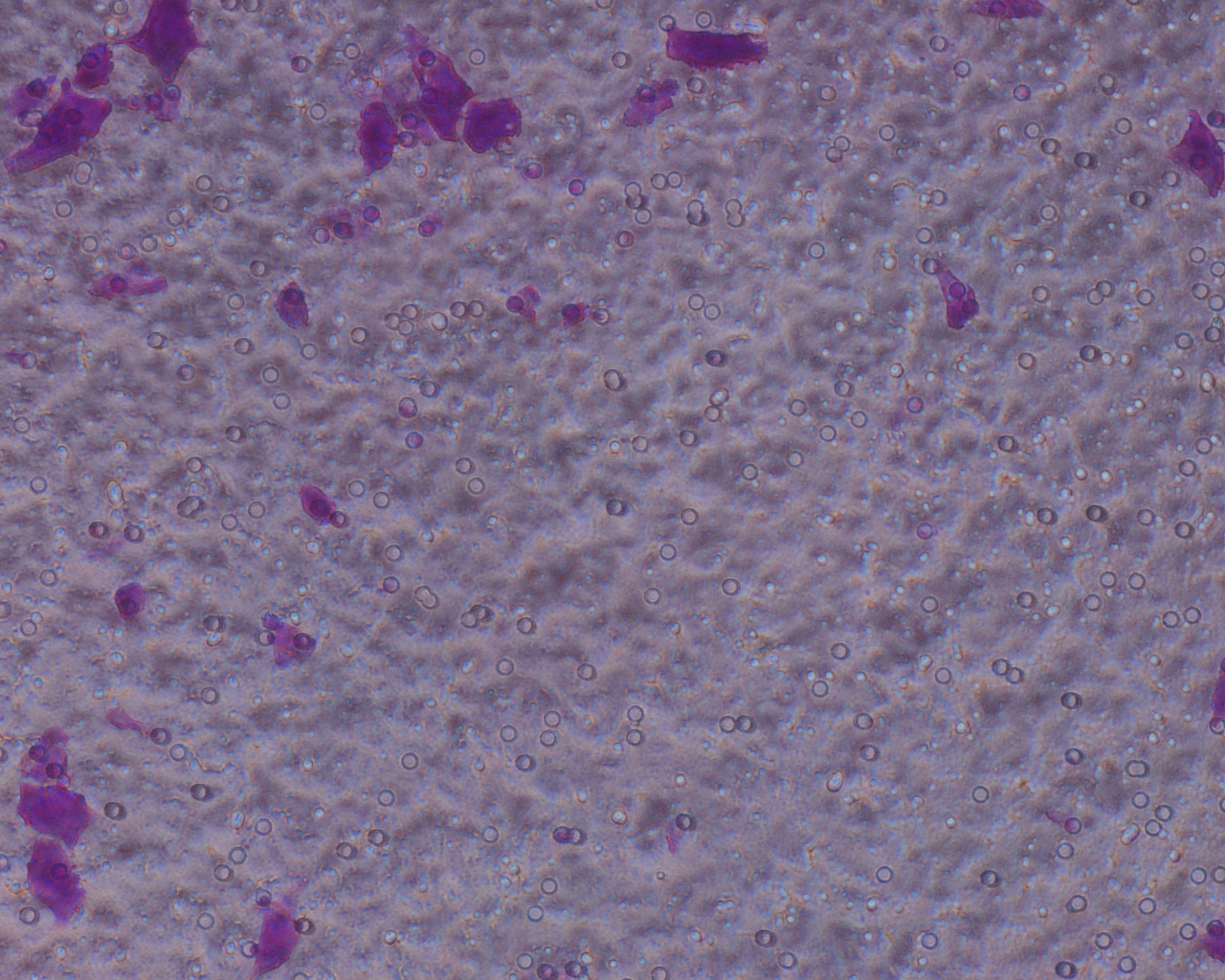

Supplement: S4 File — (ZIP) [file pgen.1010332.s004.zip › S4 File/S26 Migration shvec-1.tif]

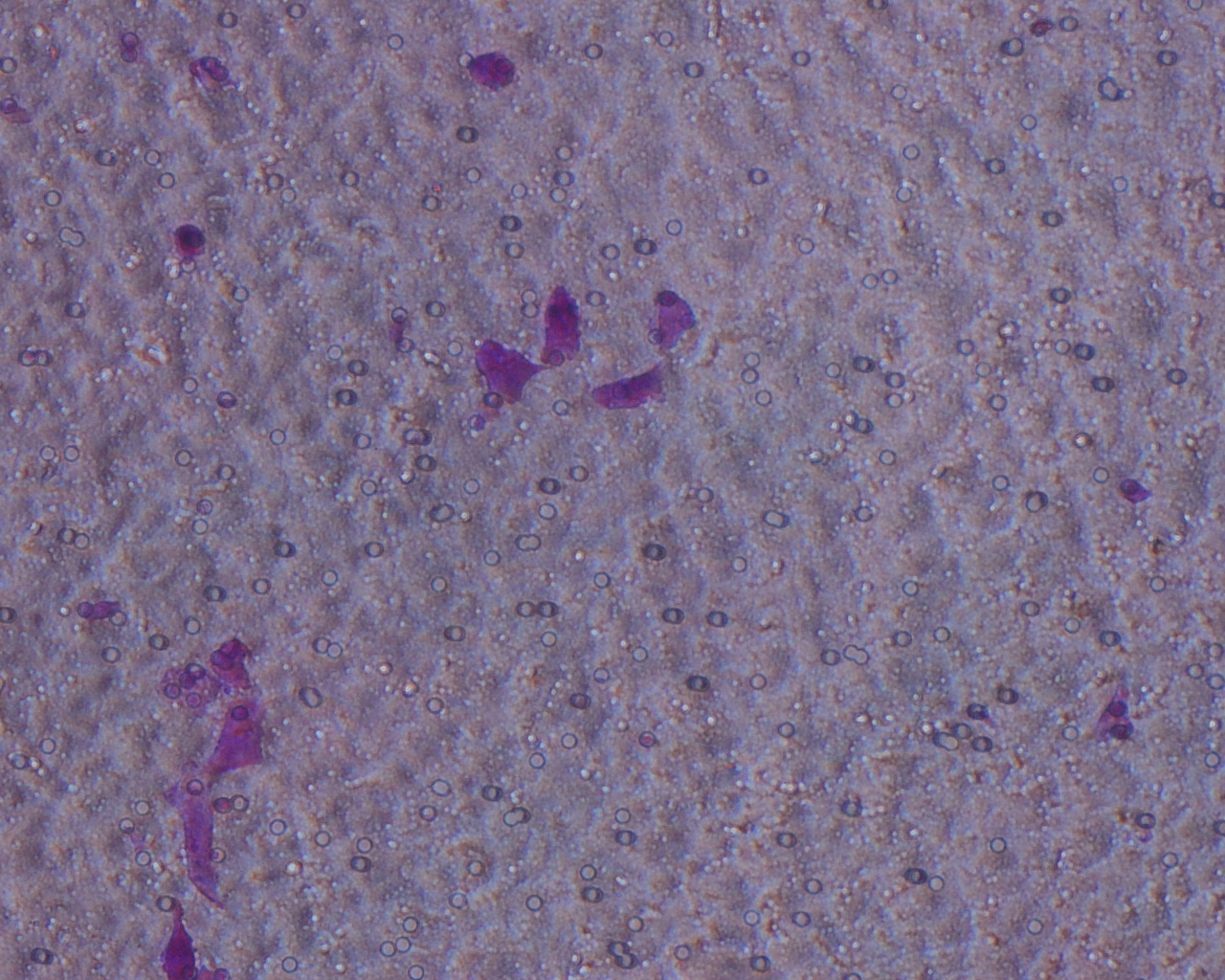

Supplement: S4 File — (ZIP) [file pgen.1010332.s004.zip › S4 File/S26 Migration shvec-2.tif]

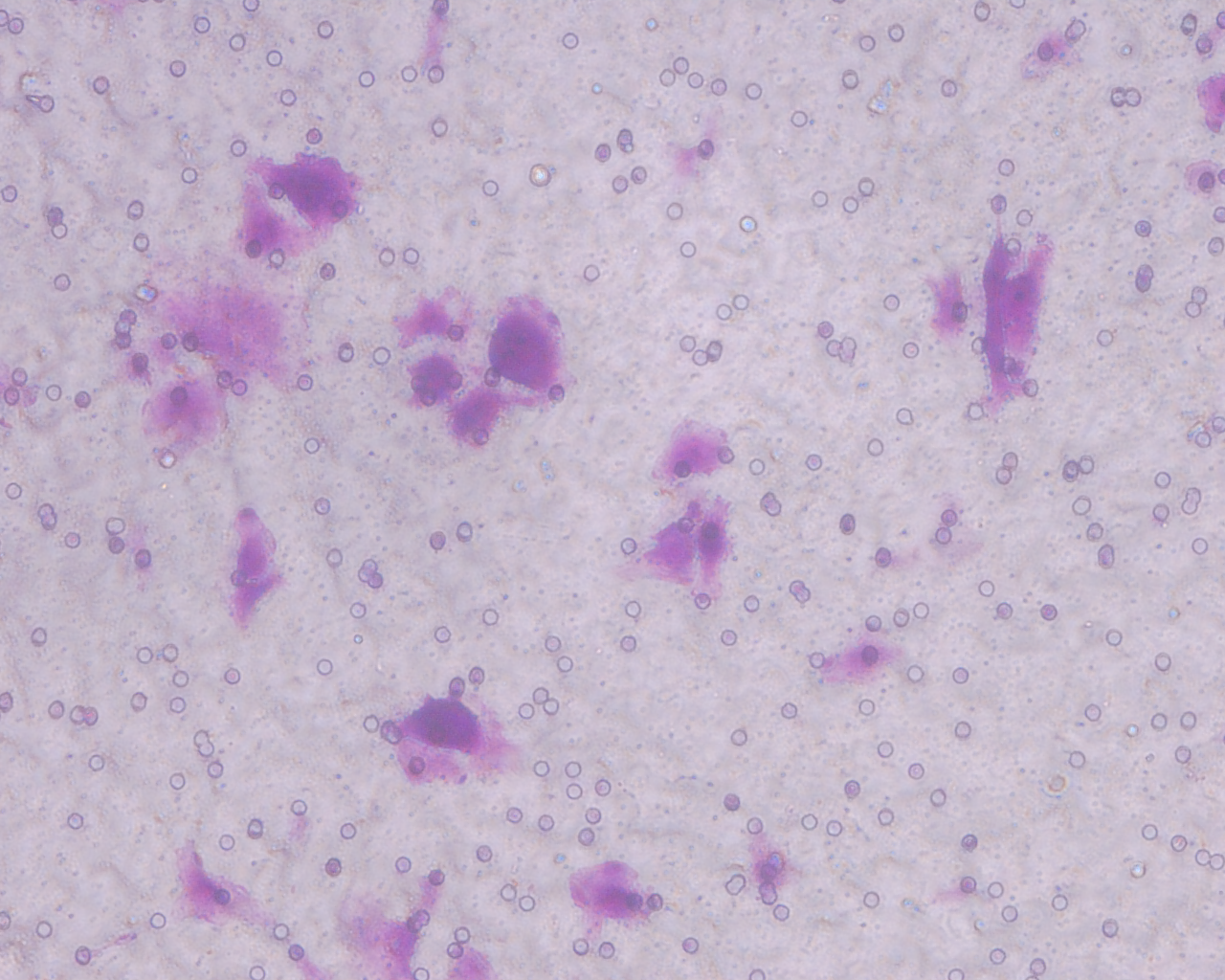

Supplement: S5 File — (ZIP) [file pgen.1010332.s005.zip › S5 File/Invasion NKILA.tif]

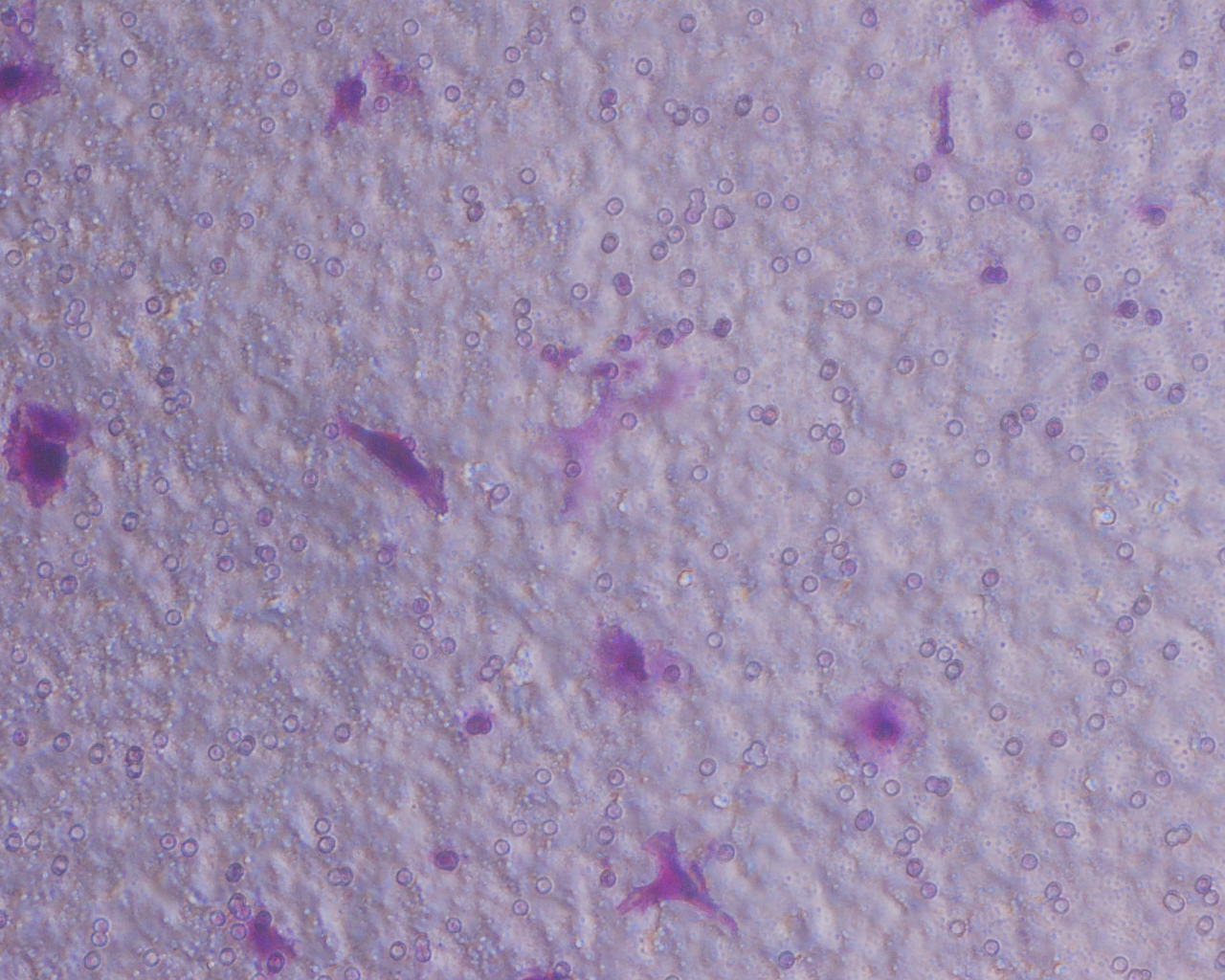

Supplement: S5 File — (ZIP) [file pgen.1010332.s005.zip › S5 File/Invasion NKILA+JSH.tif]

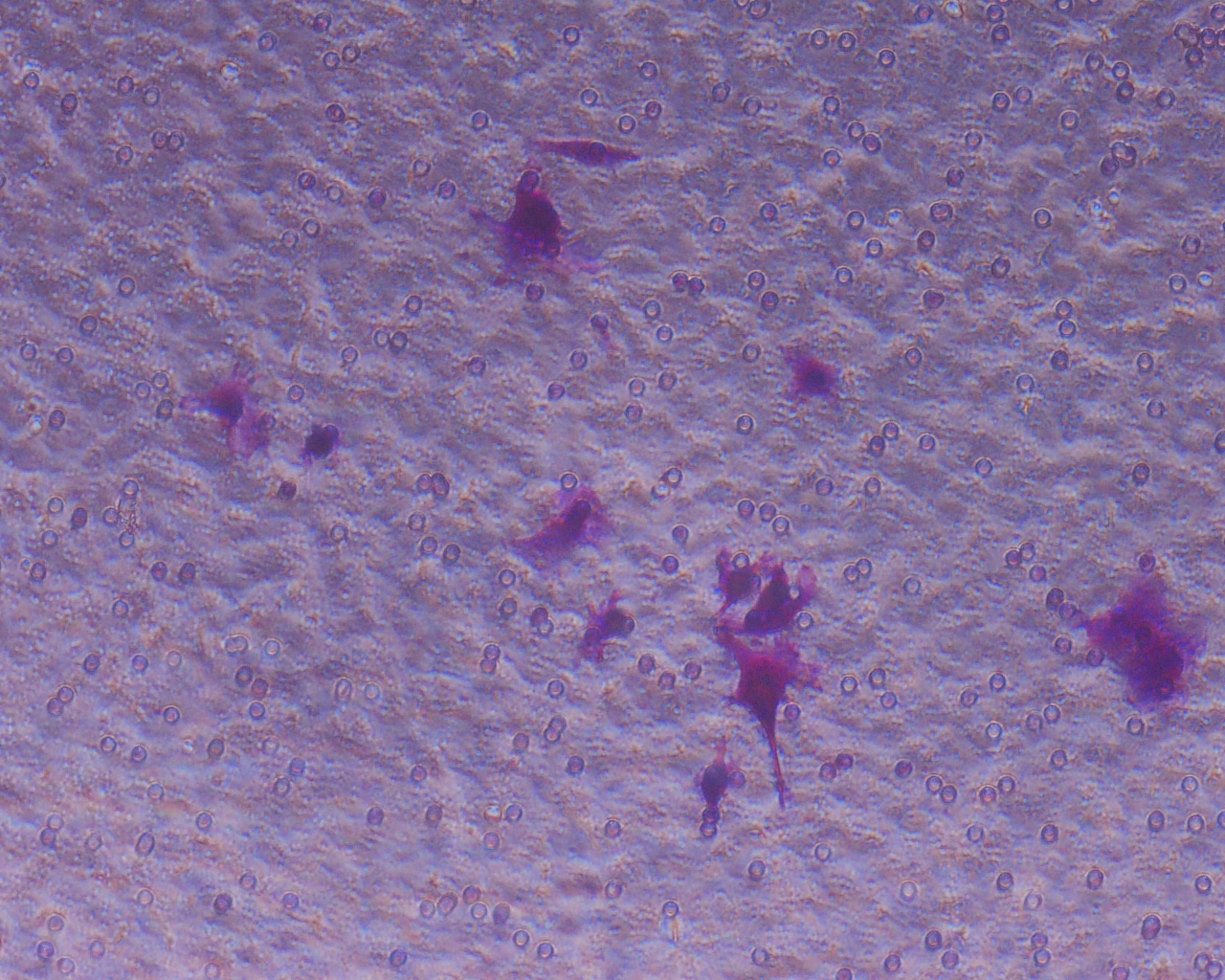

Supplement: S5 File — (ZIP) [file pgen.1010332.s005.zip › S5 File/Invasion NKILA+JSH-1.tif]

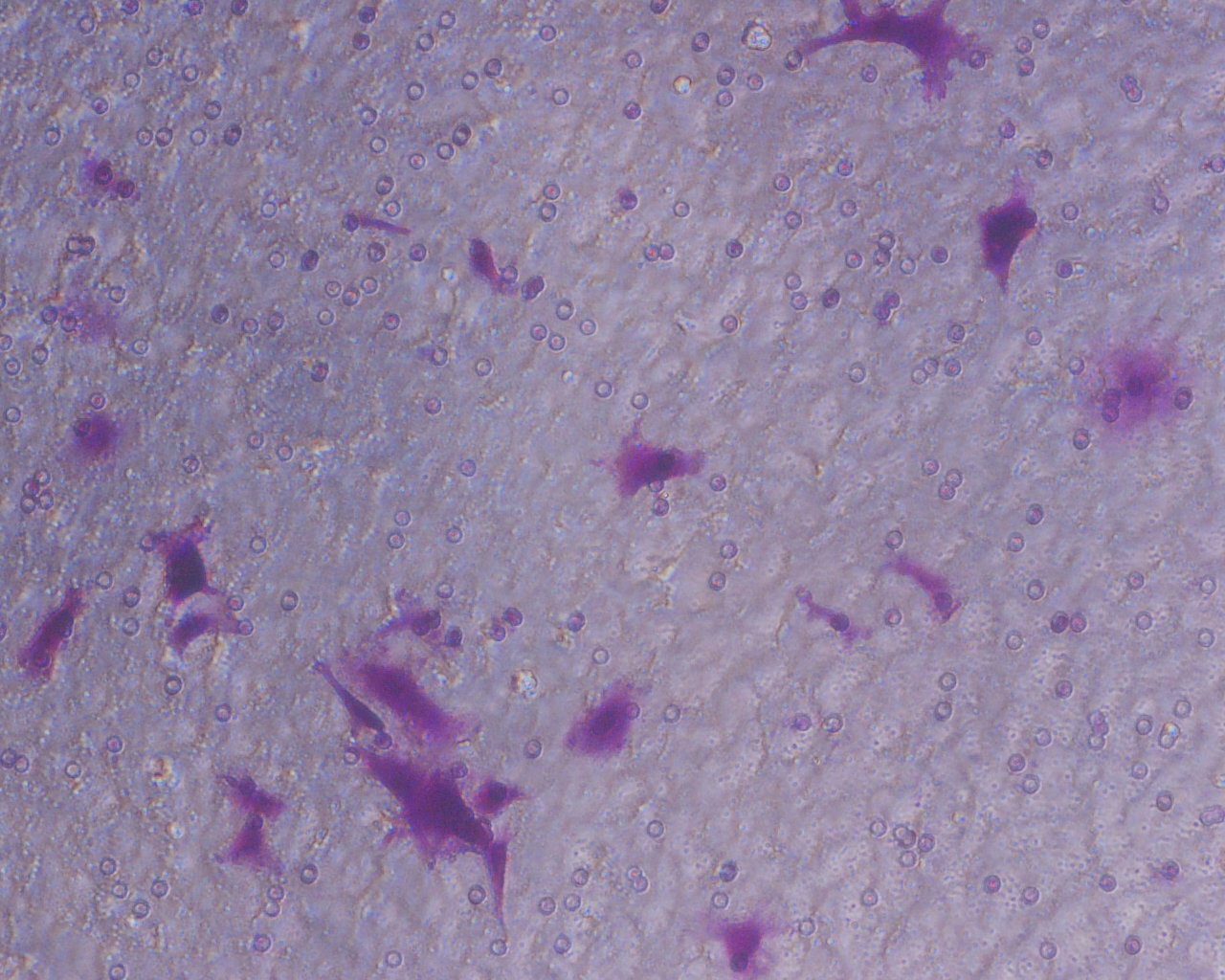

Supplement: S5 File — (ZIP) [file pgen.1010332.s005.zip › S5 File/Invasion NKILA+JSH-2.tif]

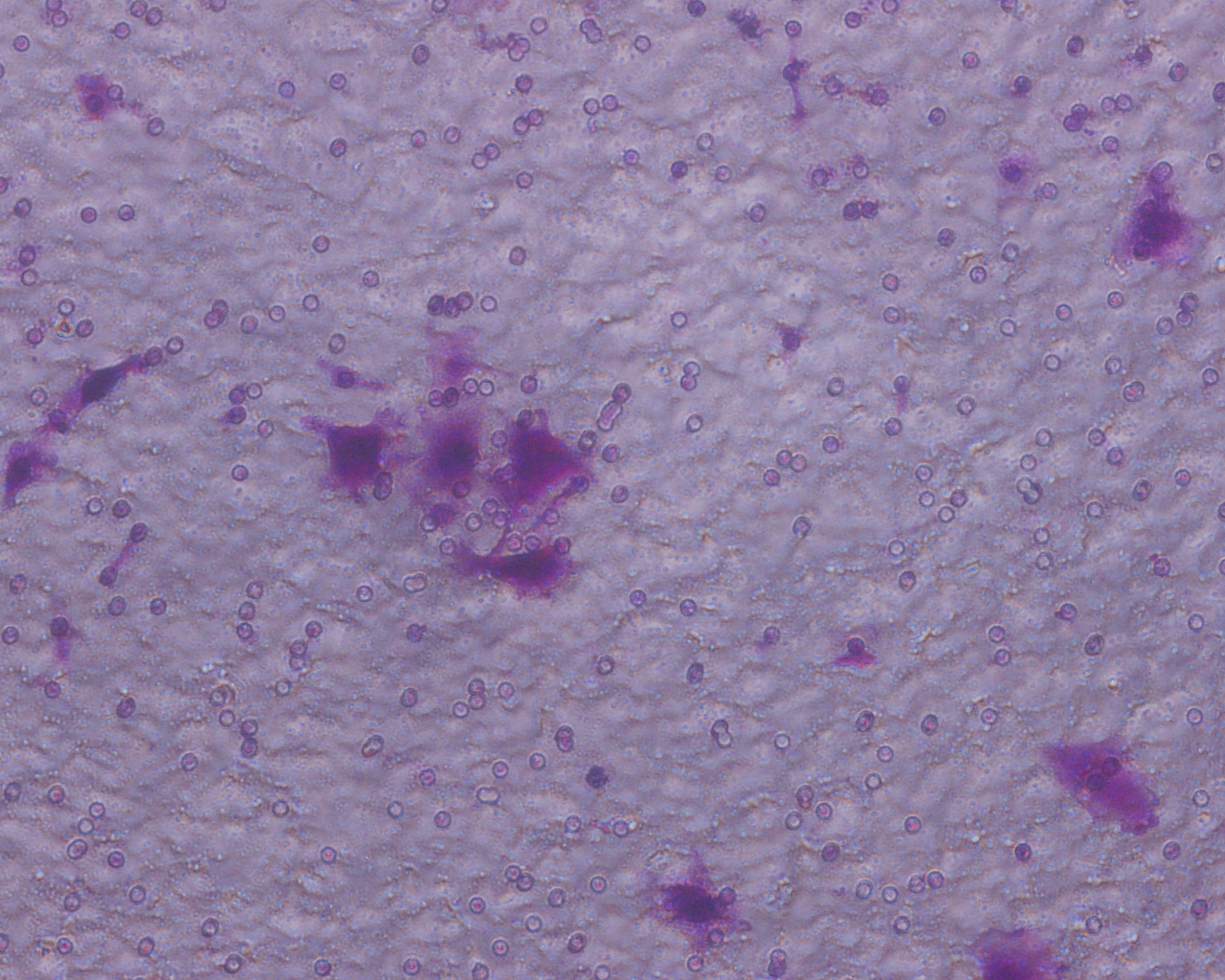

Supplement: S5 File — (ZIP) [file pgen.1010332.s005.zip › S5 File/Invasion NKILA+JSH-3.tif]

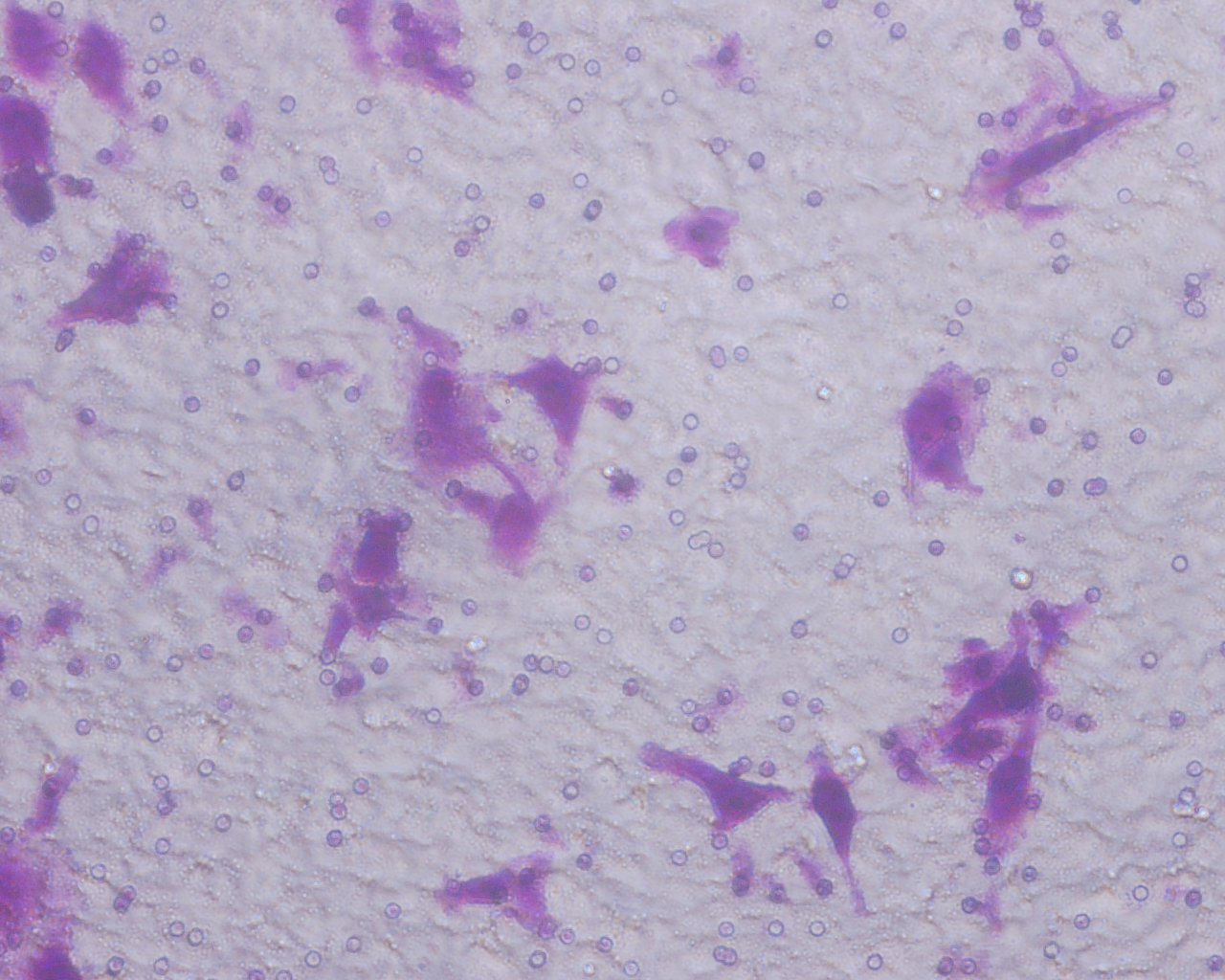

Supplement: S5 File — (ZIP) [file pgen.1010332.s005.zip › S5 File/Invasion NKILA+SC3060.tif]

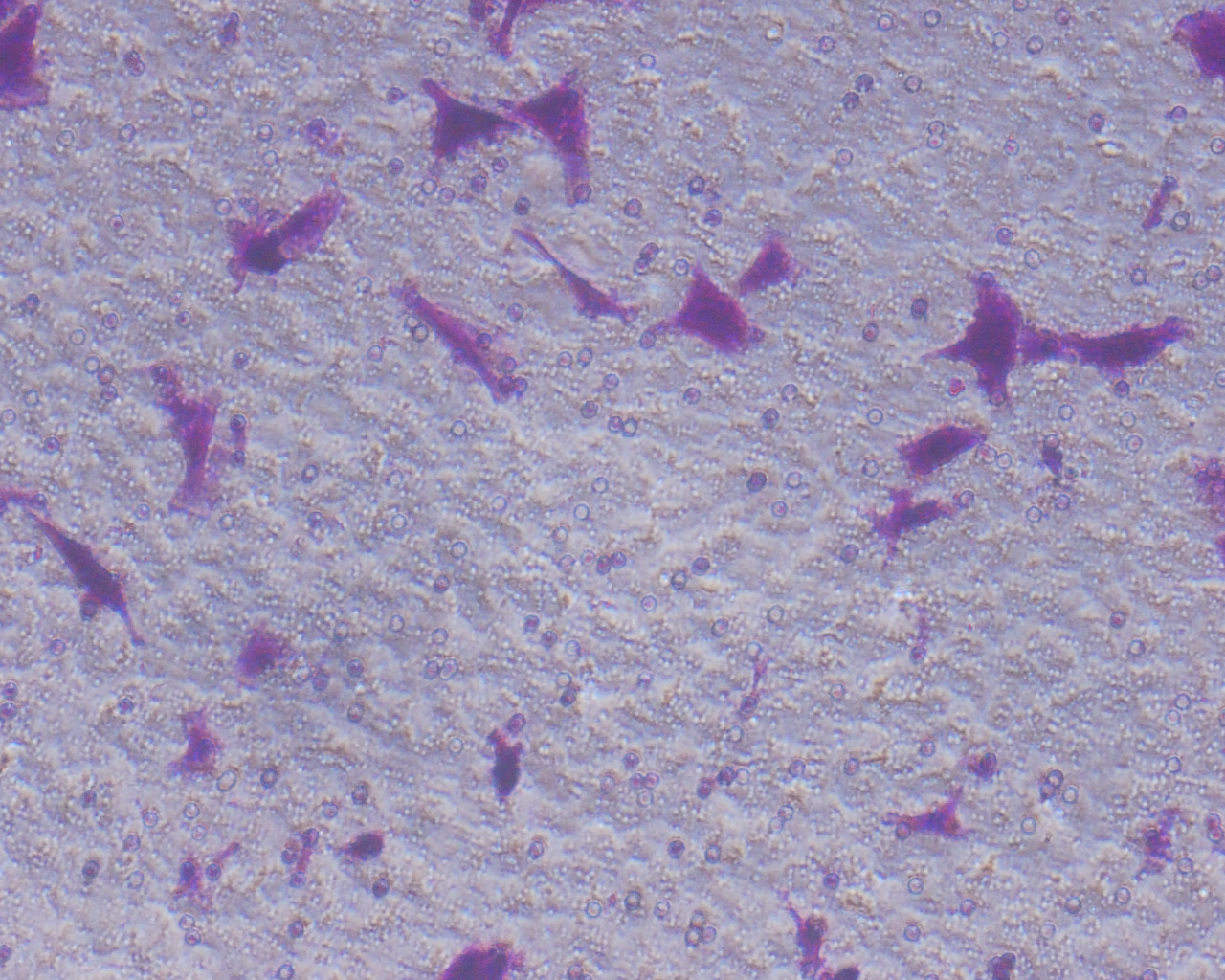

Supplement: S5 File — (ZIP) [file pgen.1010332.s005.zip › S5 File/Invasion NKILA+SC3060-1.tif]

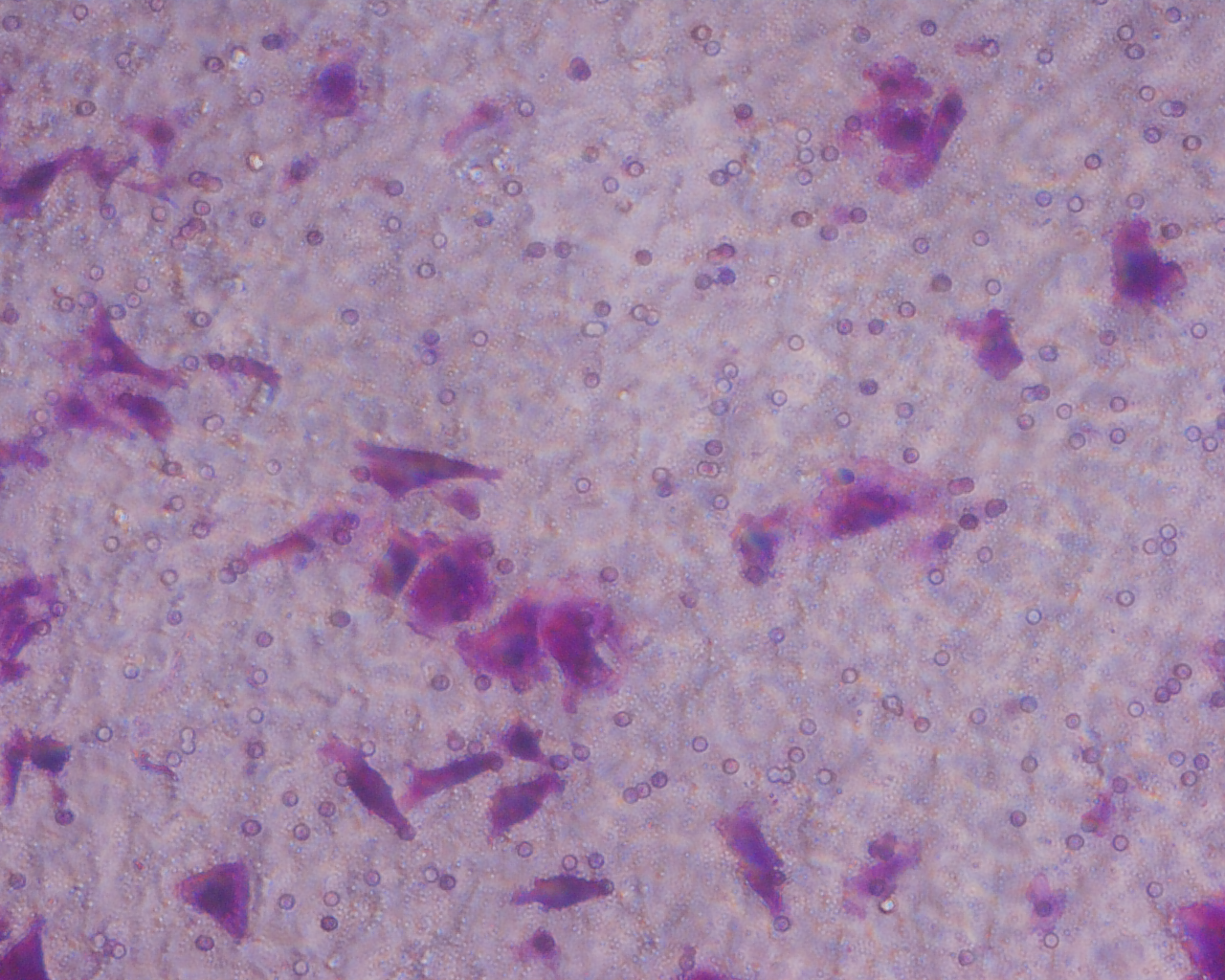

Supplement: S5 File — (ZIP) [file pgen.1010332.s005.zip › S5 File/Invasion NKILA+SC3060-2.tif]

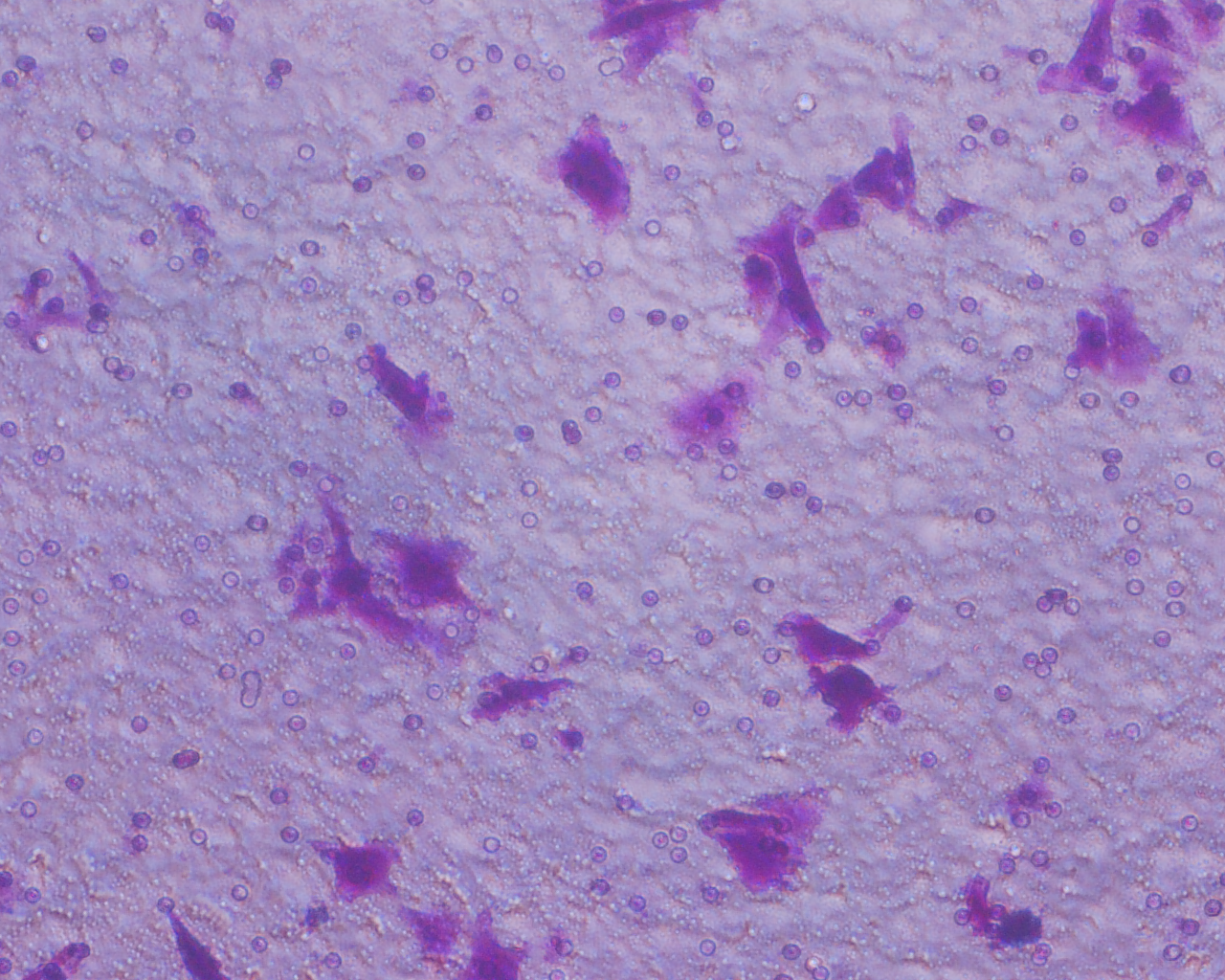

Supplement: S5 File — (ZIP) [file pgen.1010332.s005.zip › S5 File/Invasion NKILA+SC3060-3.tif]

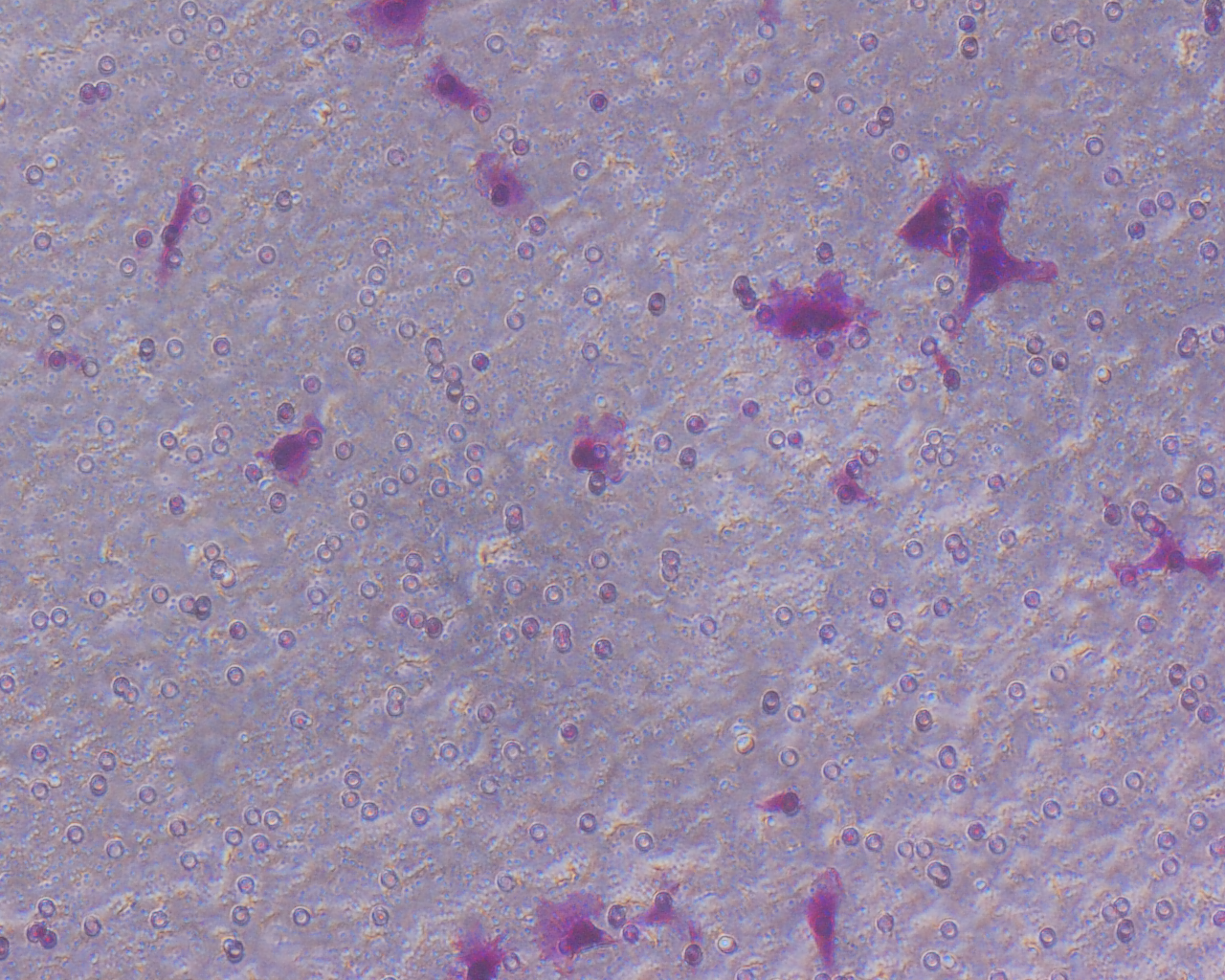

Supplement: S5 File — (ZIP) [file pgen.1010332.s005.zip › S5 File/Invasion NKILA-1.tif]

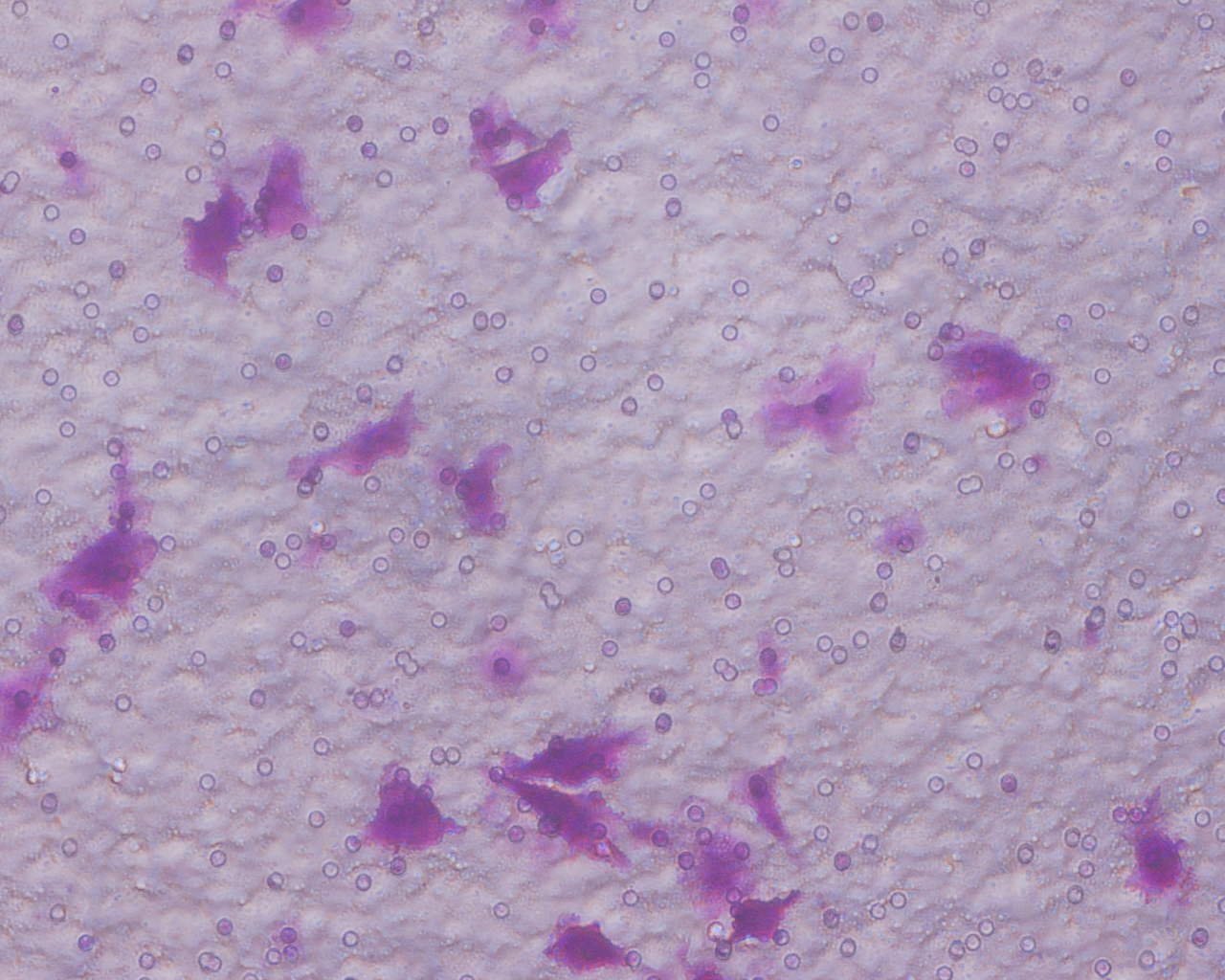

Supplement: S5 File — (ZIP) [file pgen.1010332.s005.zip › S5 File/Invasion NKILA-2.tif]

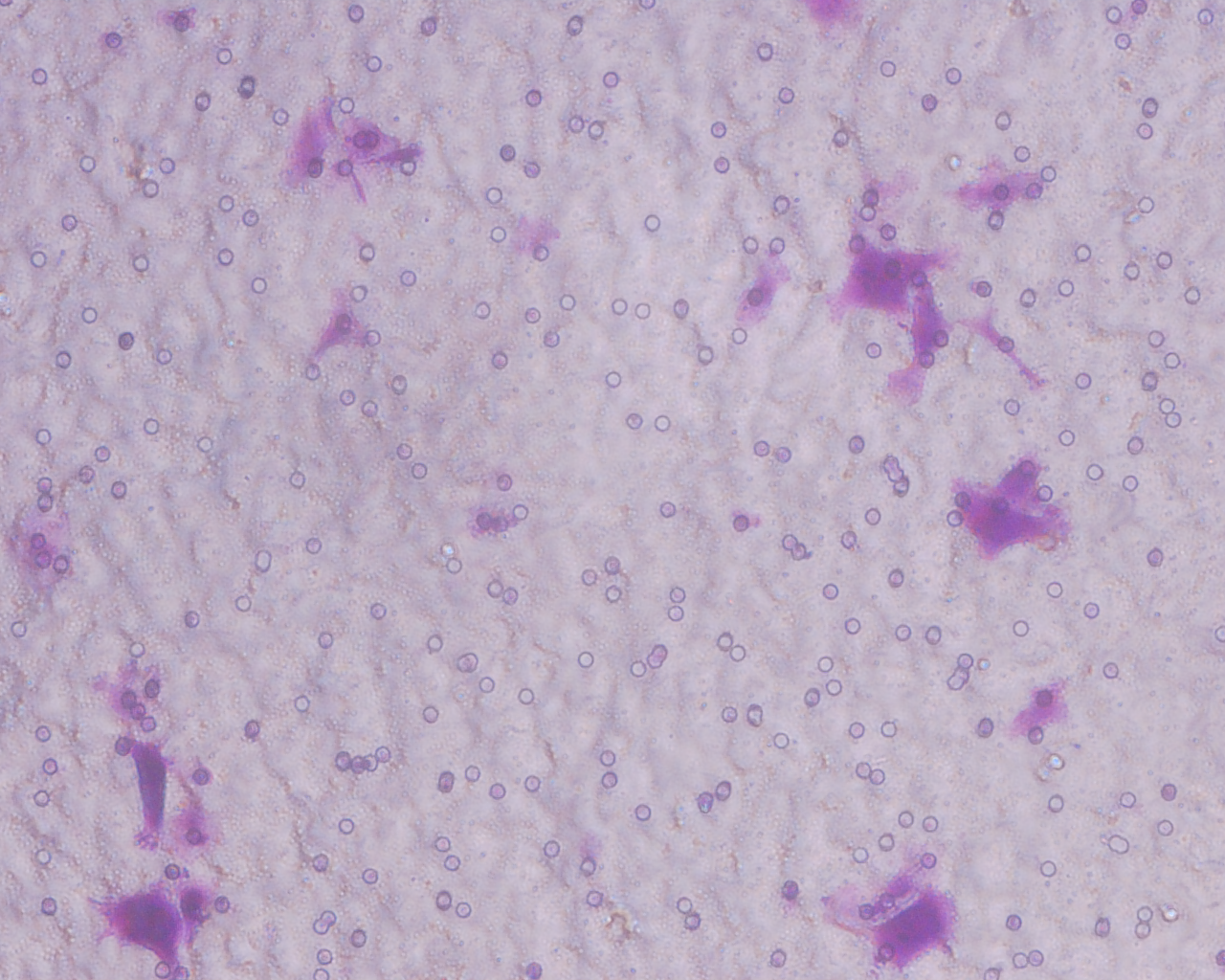

Supplement: S5 File — (ZIP) [file pgen.1010332.s005.zip › S5 File/Invasion NKILA-3.tif]

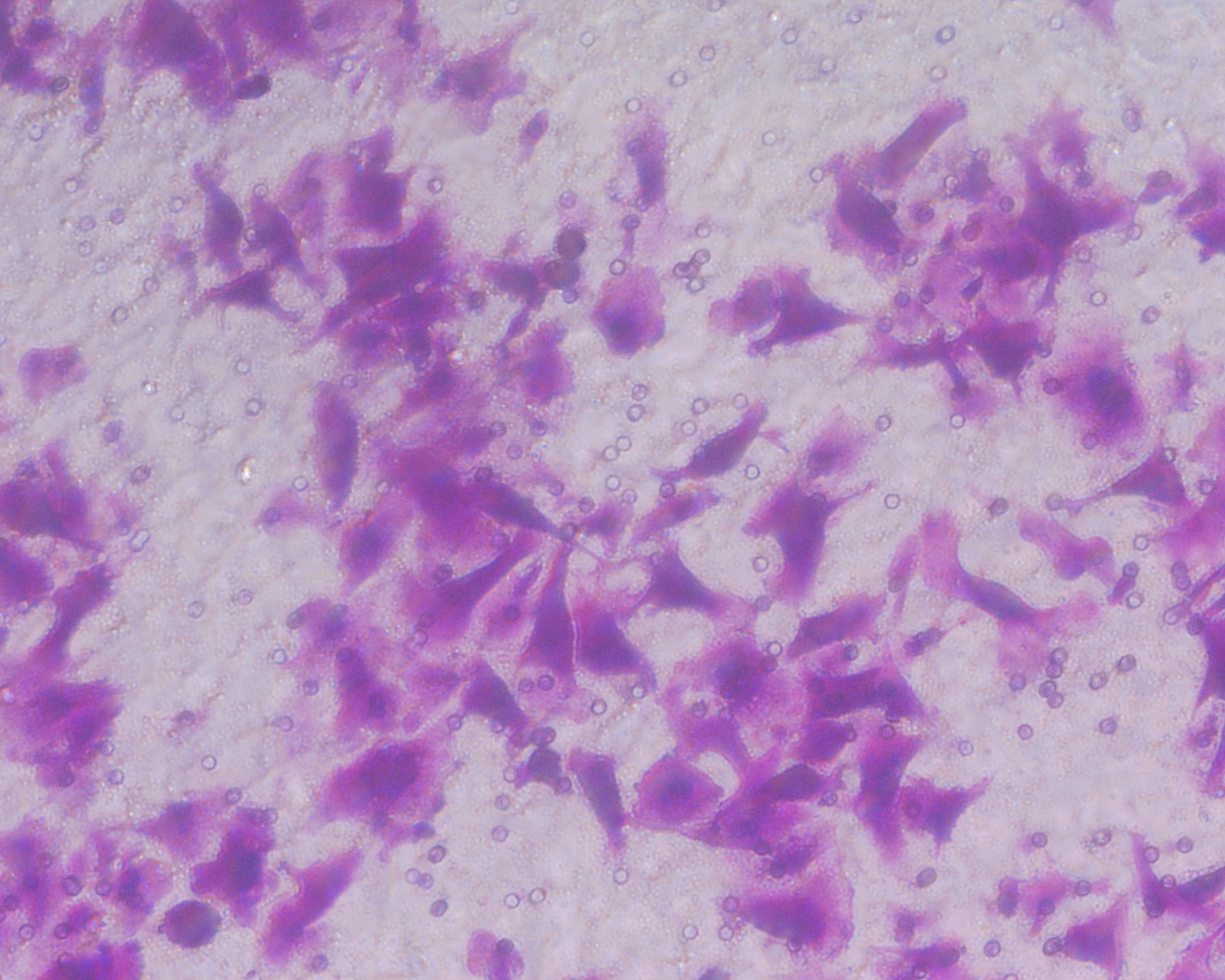

Supplement: S5 File — (ZIP) [file pgen.1010332.s005.zip › S5 File/Invasion VEC.tif]

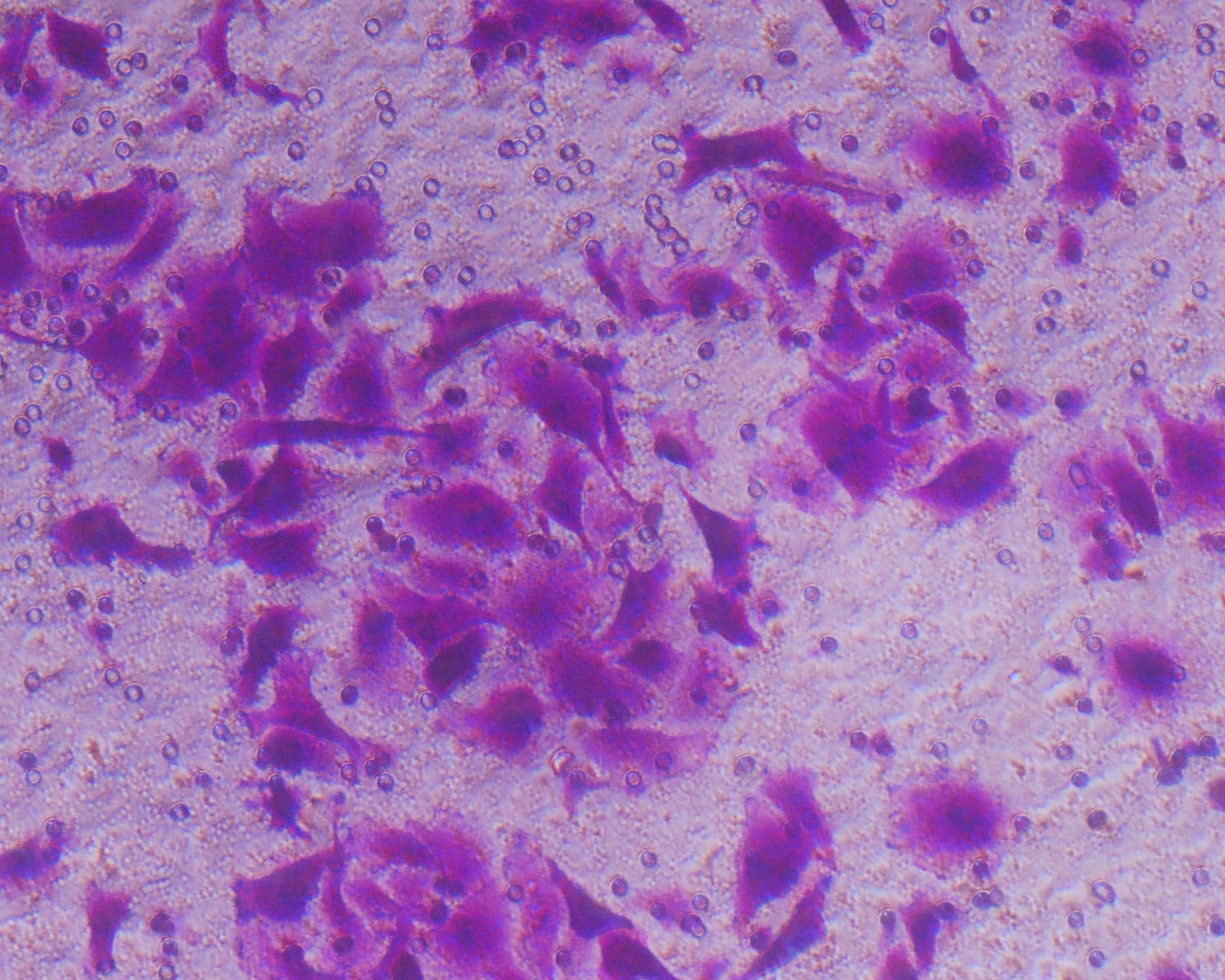

Supplement: S5 File — (ZIP) [file pgen.1010332.s005.zip › S5 File/Invasion VEC-1.tif]

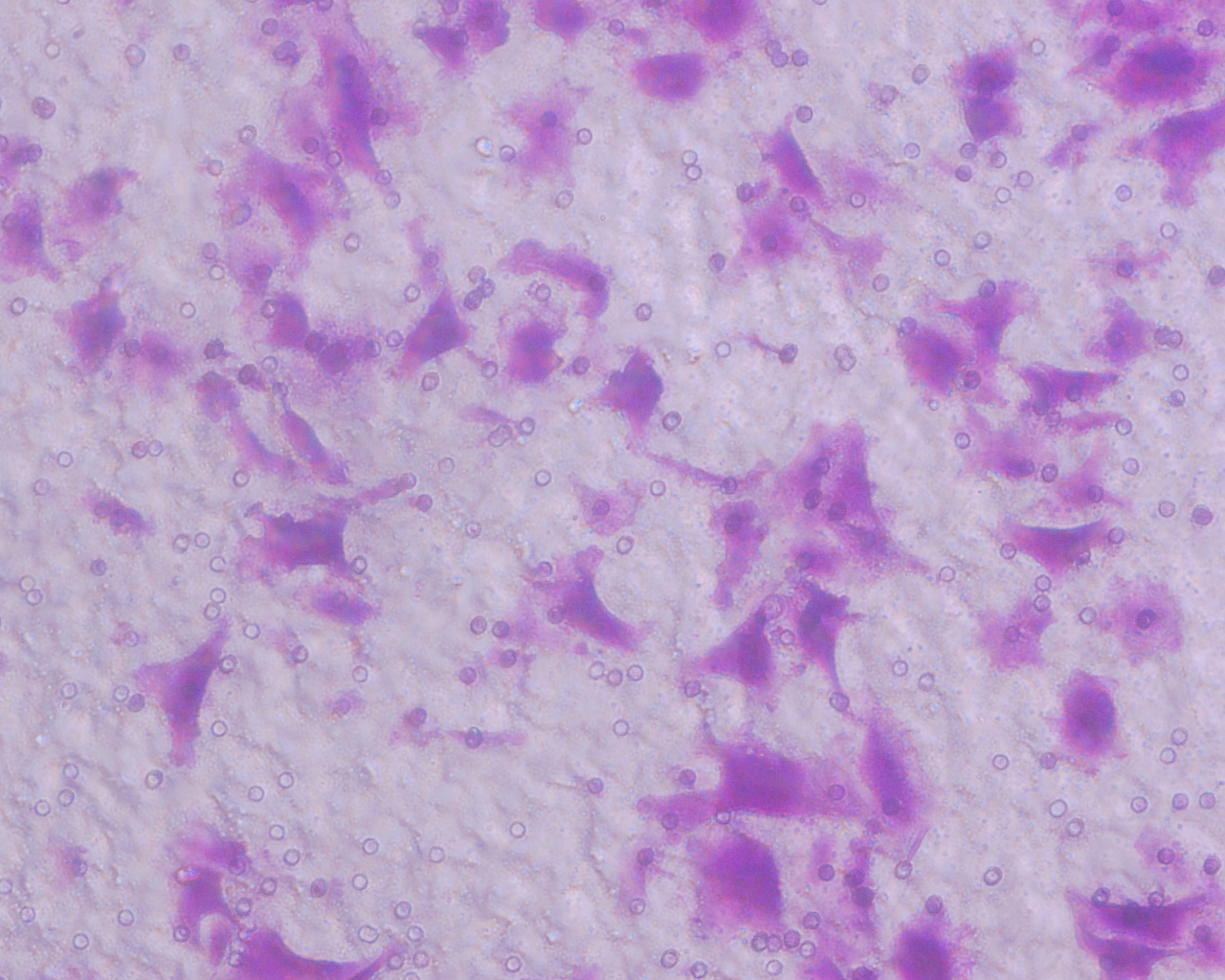

Supplement: S5 File — (ZIP) [file pgen.1010332.s005.zip › S5 File/Invasion VEC-2.tif]

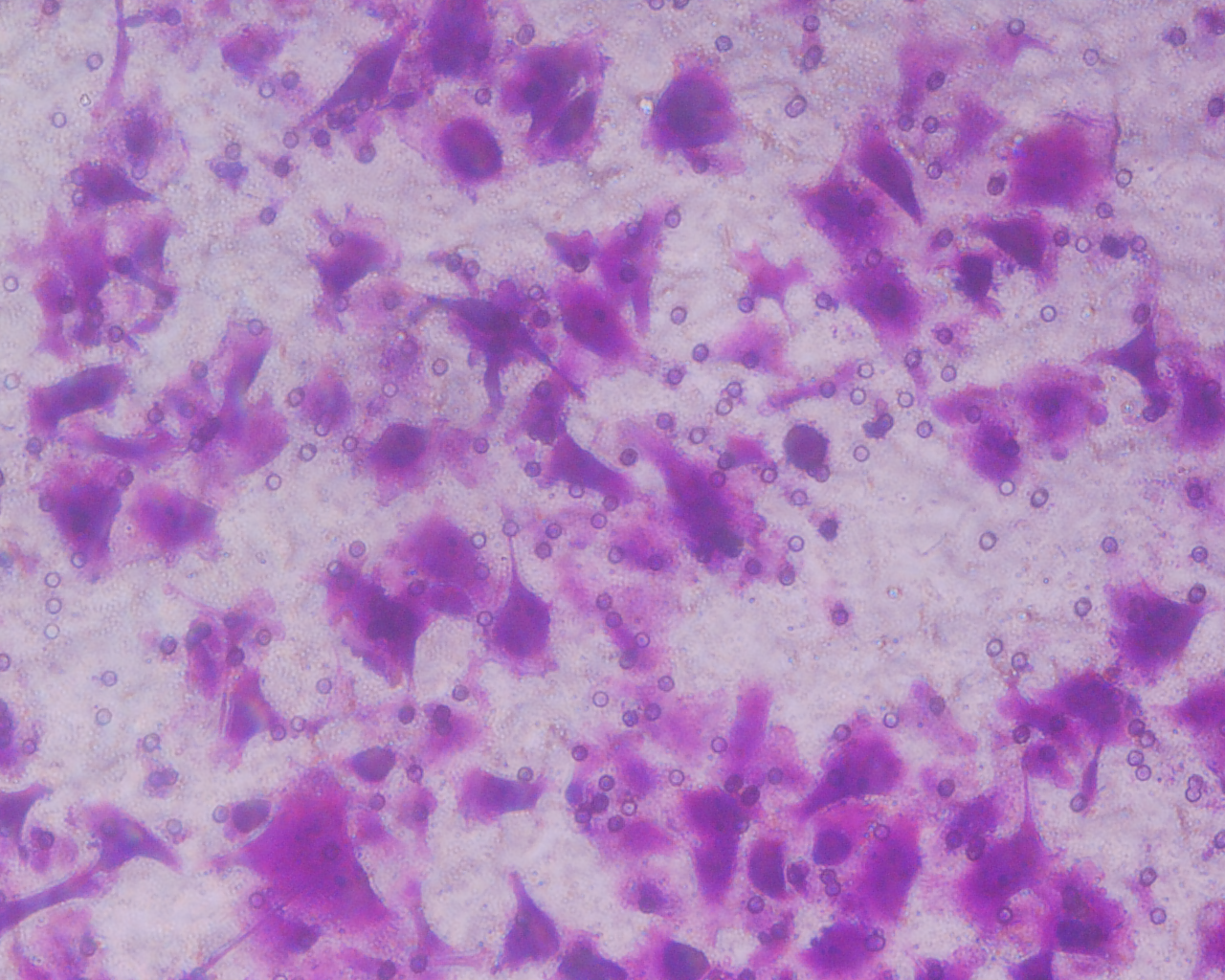

Supplement: S5 File — (ZIP) [file pgen.1010332.s005.zip › S5 File/Invasion VEC-3.tif]

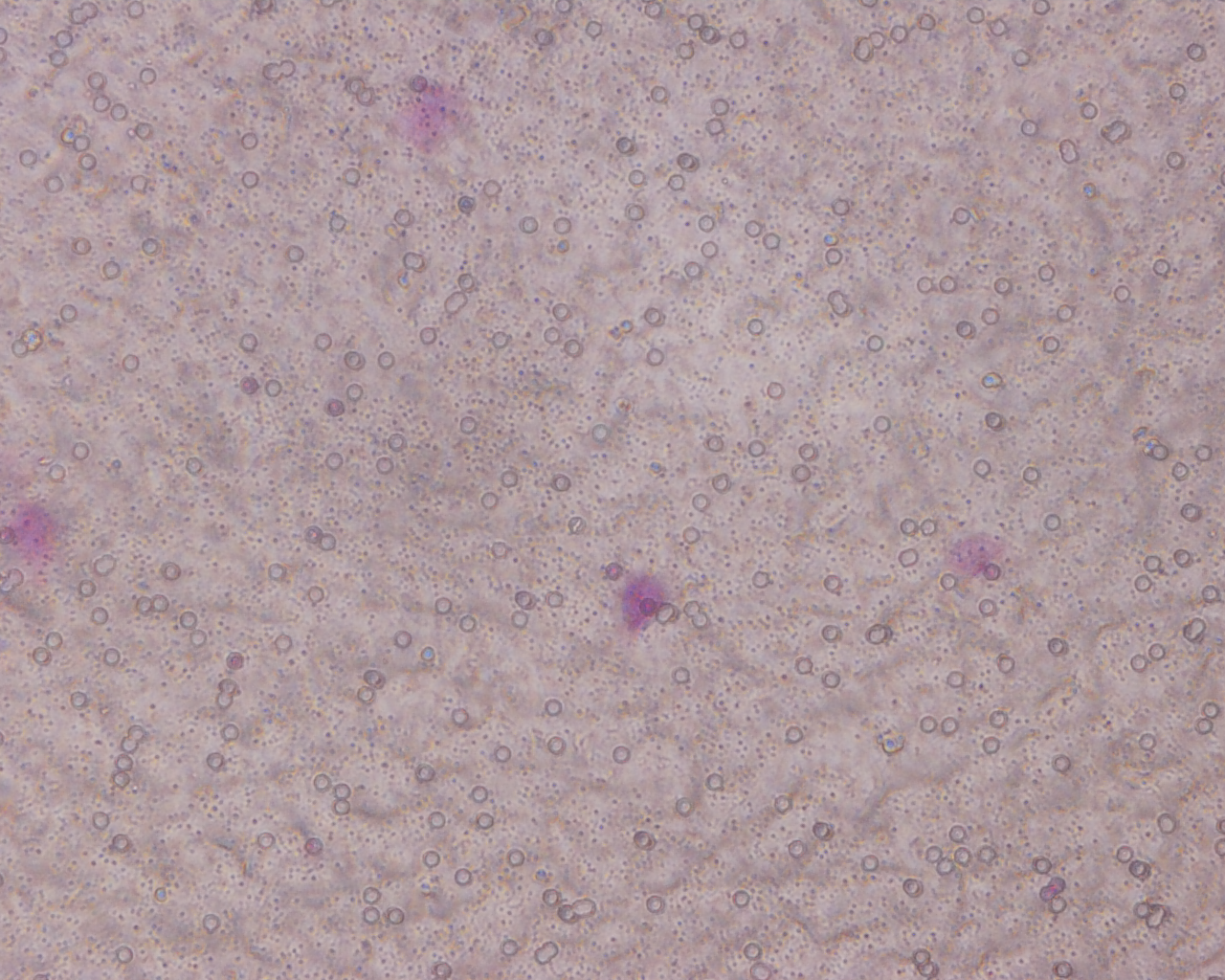

Supplement: S5 File — (ZIP) [file pgen.1010332.s005.zip › S5 File/Migration NKILA.tif]

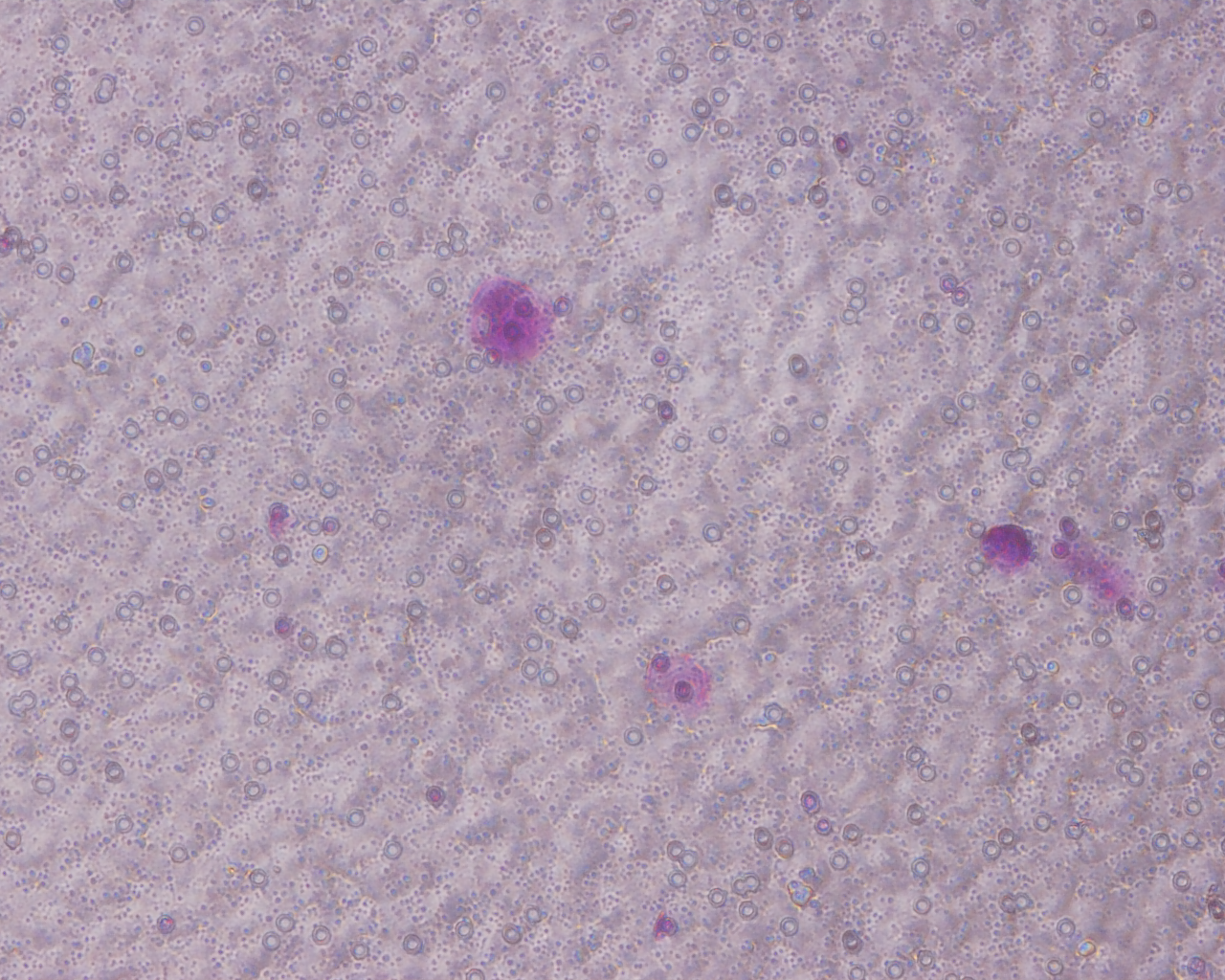

Supplement: S5 File — (ZIP) [file pgen.1010332.s005.zip › S5 File/Migration NKILA+JSH.tif]

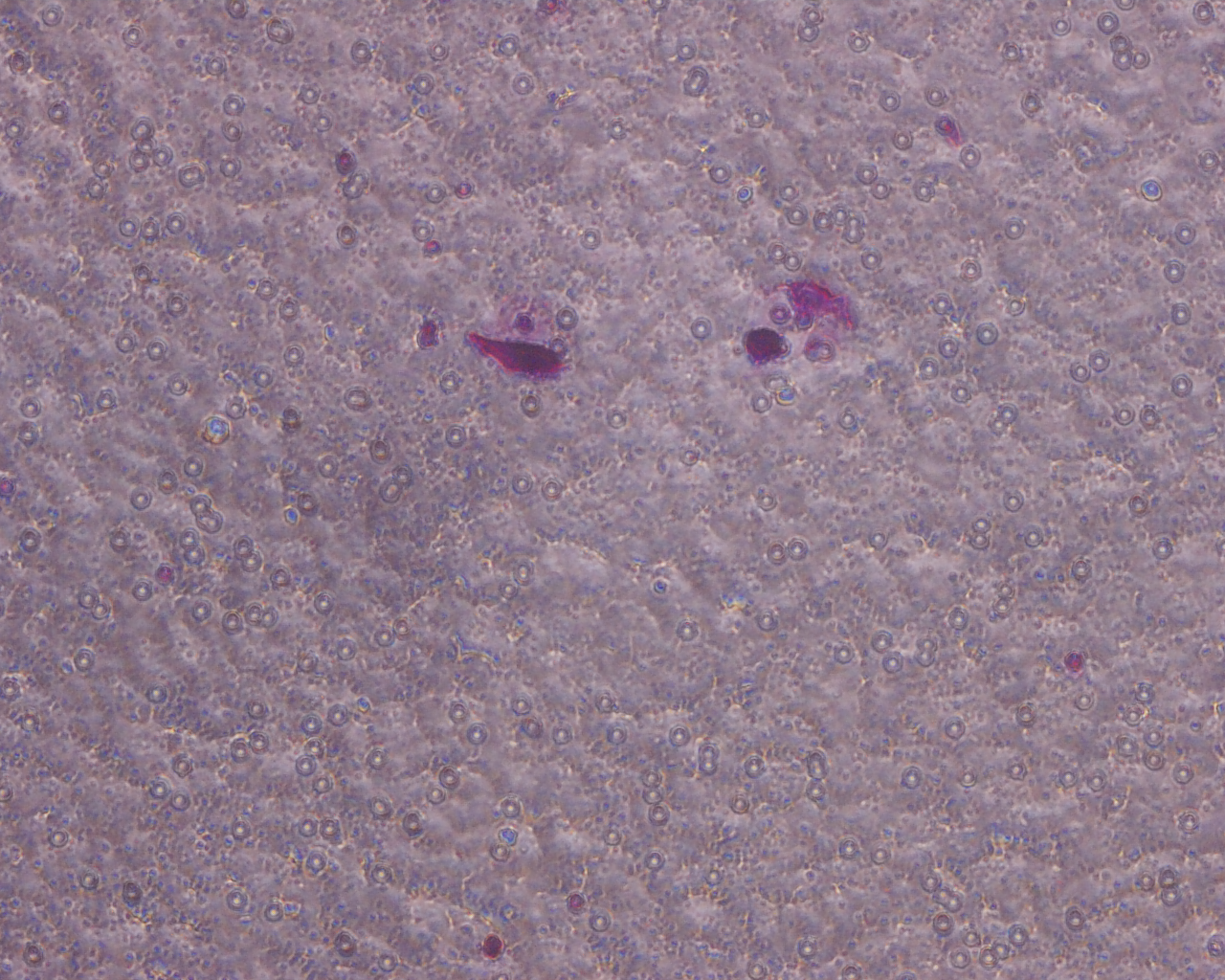

Supplement: S5 File — (ZIP) [file pgen.1010332.s005.zip › S5 File/Migration NKILA+JSH-1.tif]

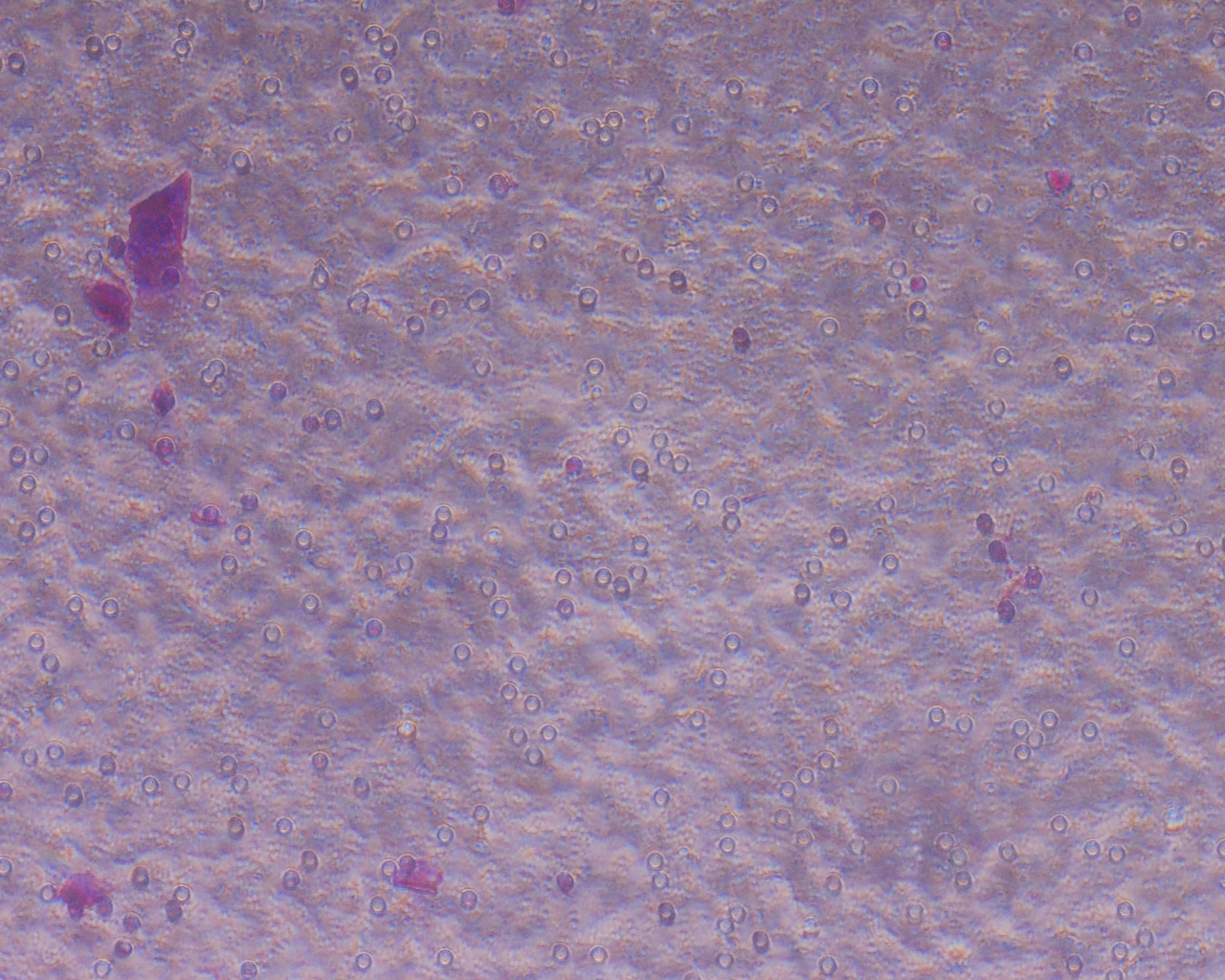

Supplement: S5 File — (ZIP) [file pgen.1010332.s005.zip › S5 File/Migration NKILA+JSH-2.tif]

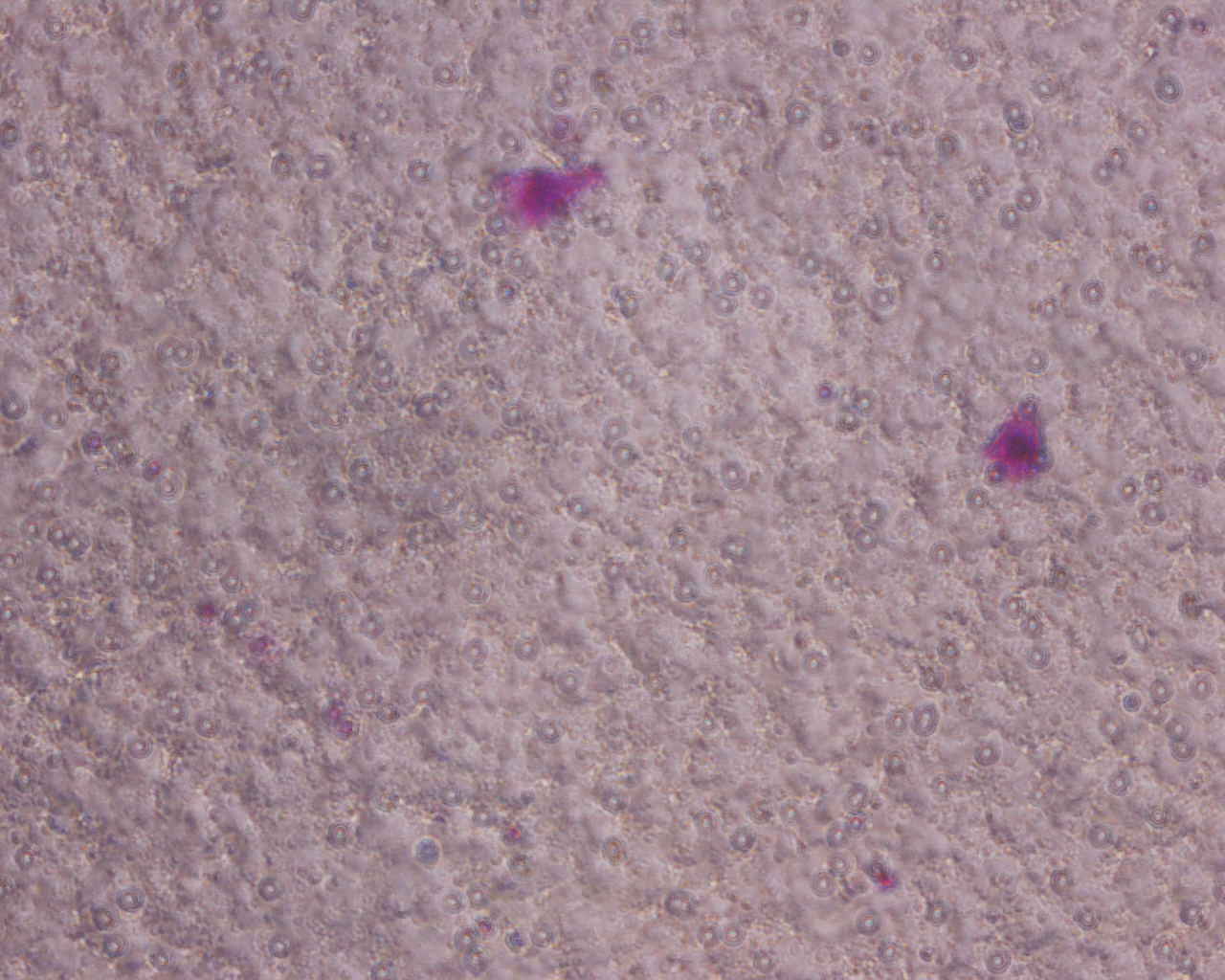

Supplement: S5 File — (ZIP) [file pgen.1010332.s005.zip › S5 File/Migration NKILA+JSH-3.tif]

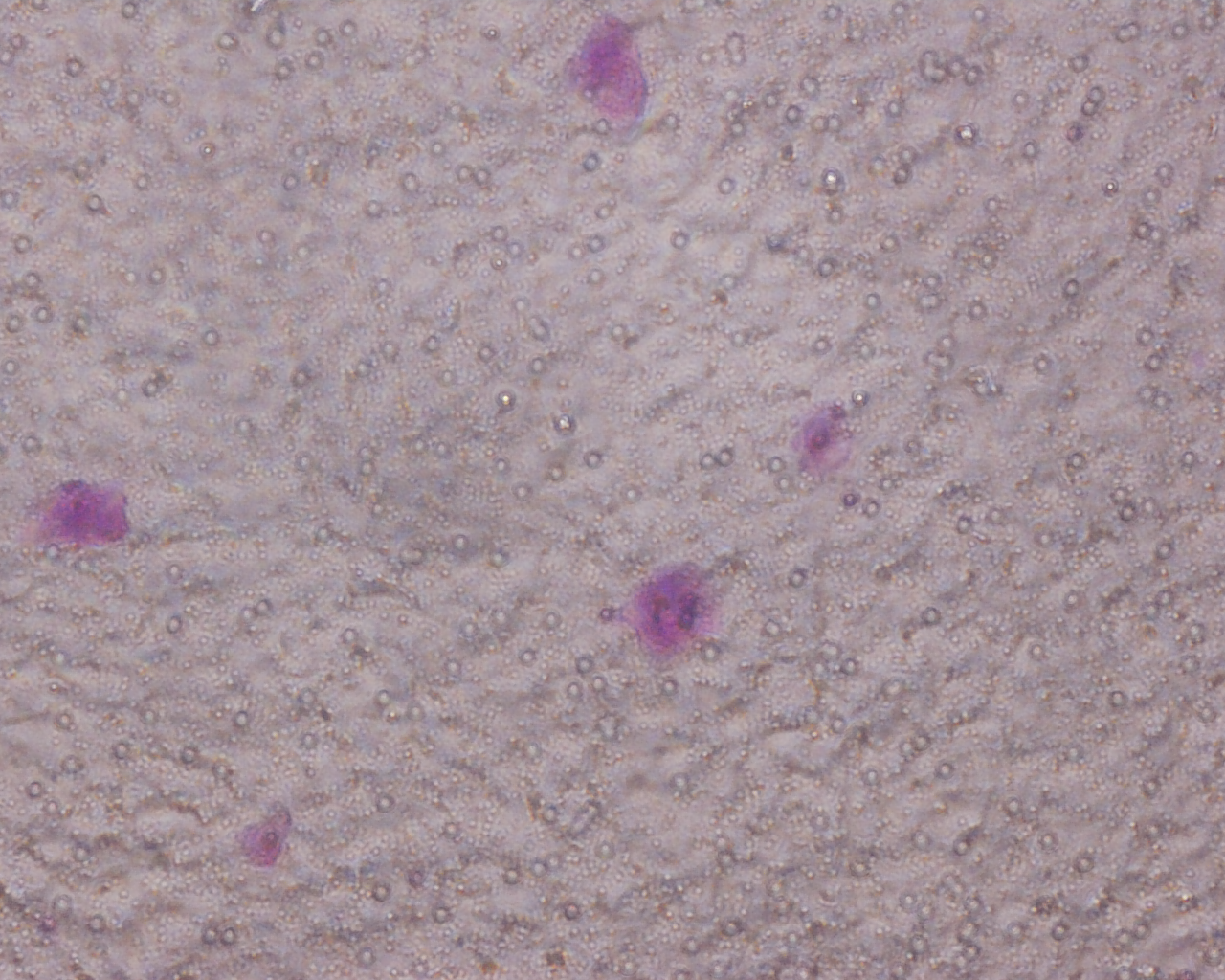

Supplement: S5 File — (ZIP) [file pgen.1010332.s005.zip › S5 File/Migration NKILA+SC3060.tif]

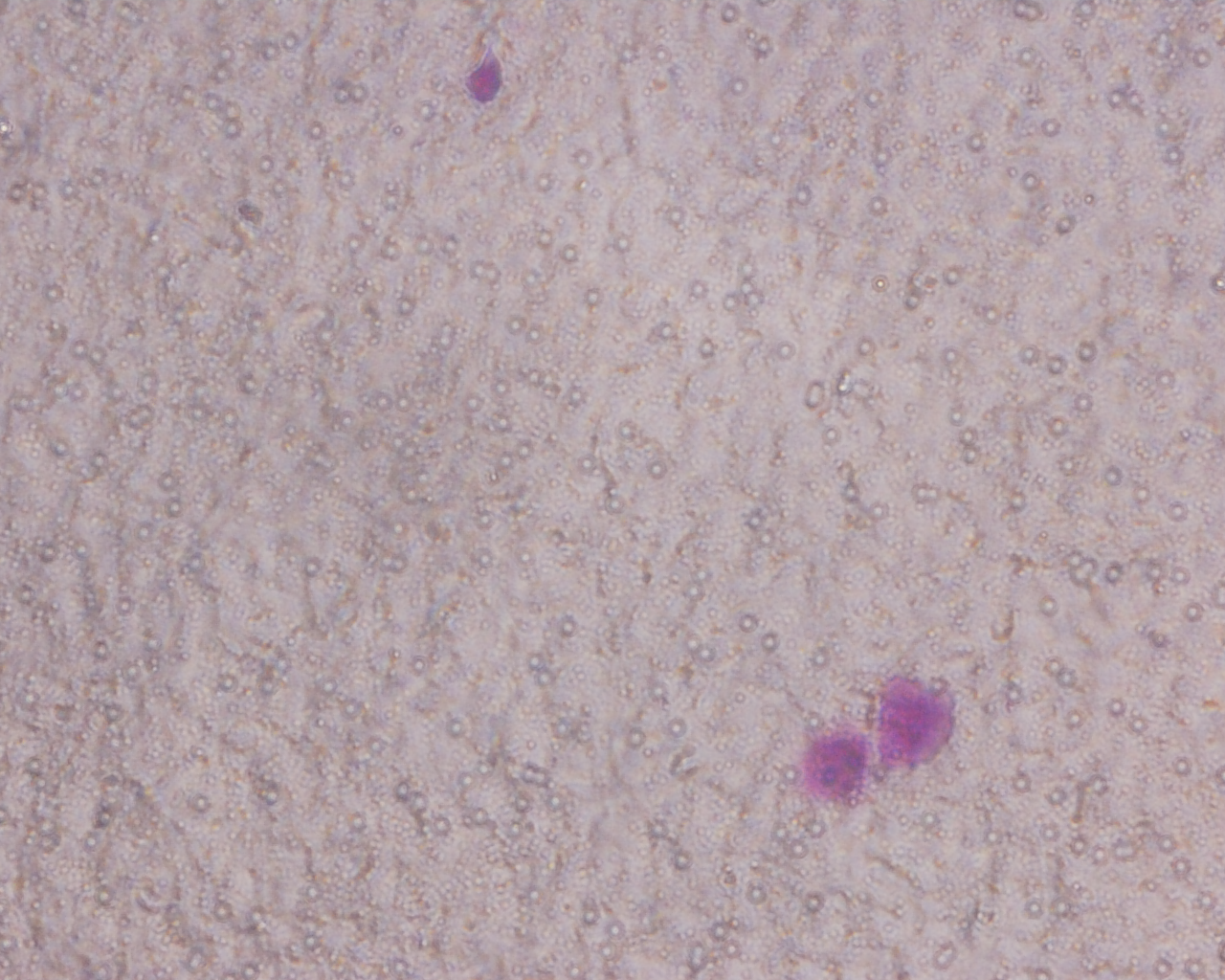

Supplement: S5 File — (ZIP) [file pgen.1010332.s005.zip › S5 File/Migration NKILA+SC3060-1.tif]

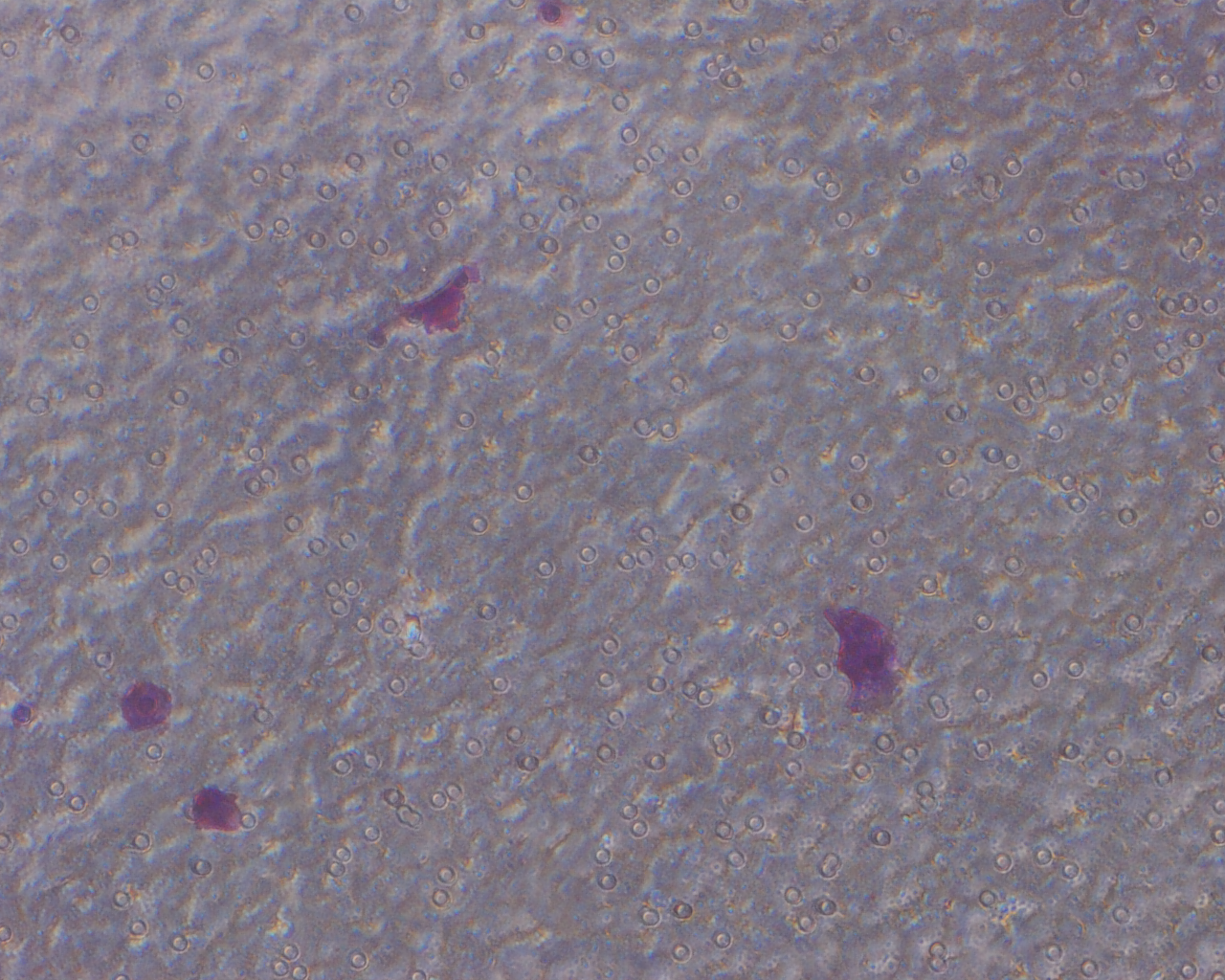

Supplement: S5 File — (ZIP) [file pgen.1010332.s005.zip › S5 File/Migration NKILA+SC3060-2.tif]

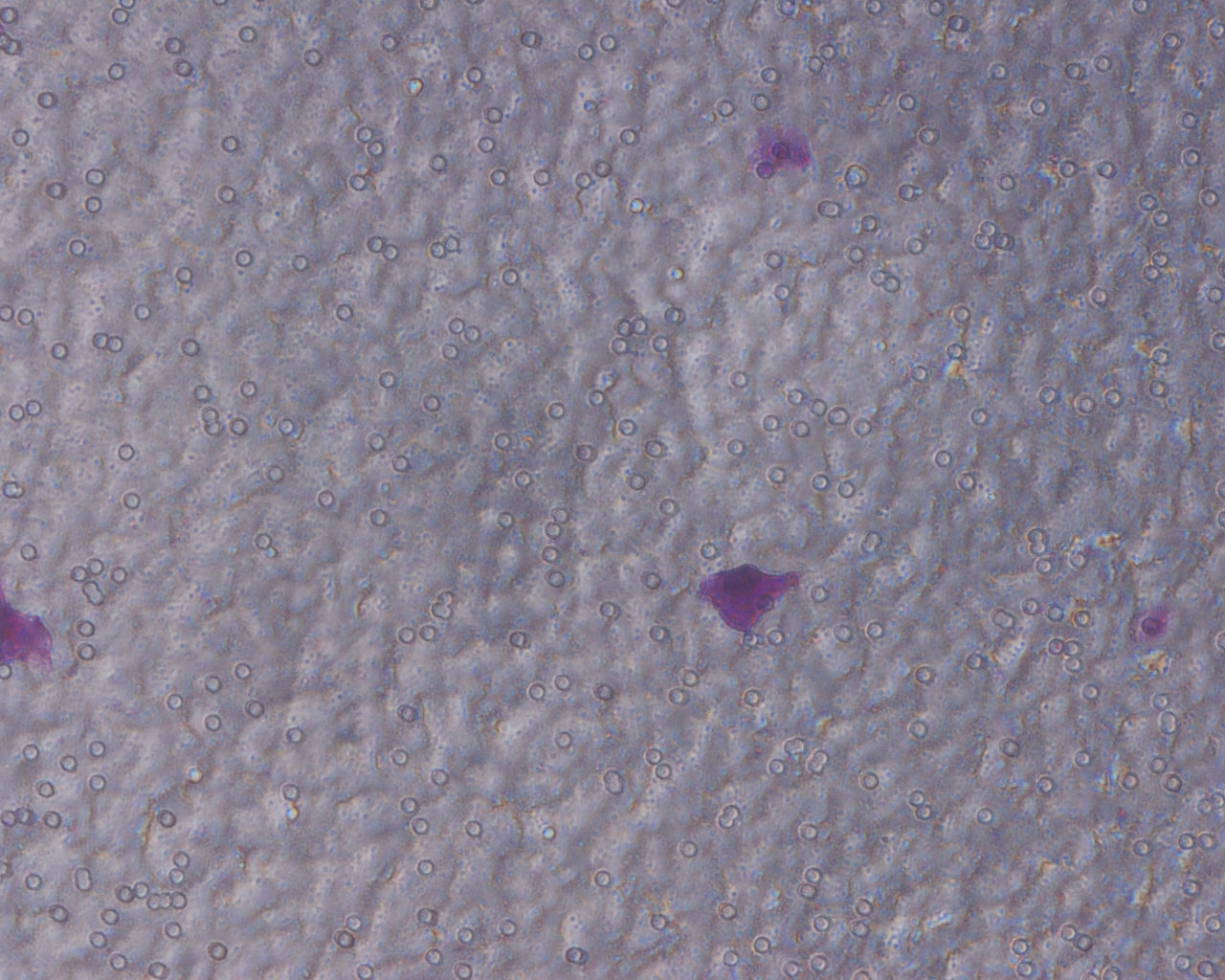

Supplement: S5 File — (ZIP) [file pgen.1010332.s005.zip › S5 File/Migration NKILA+SC3060-3.tif]

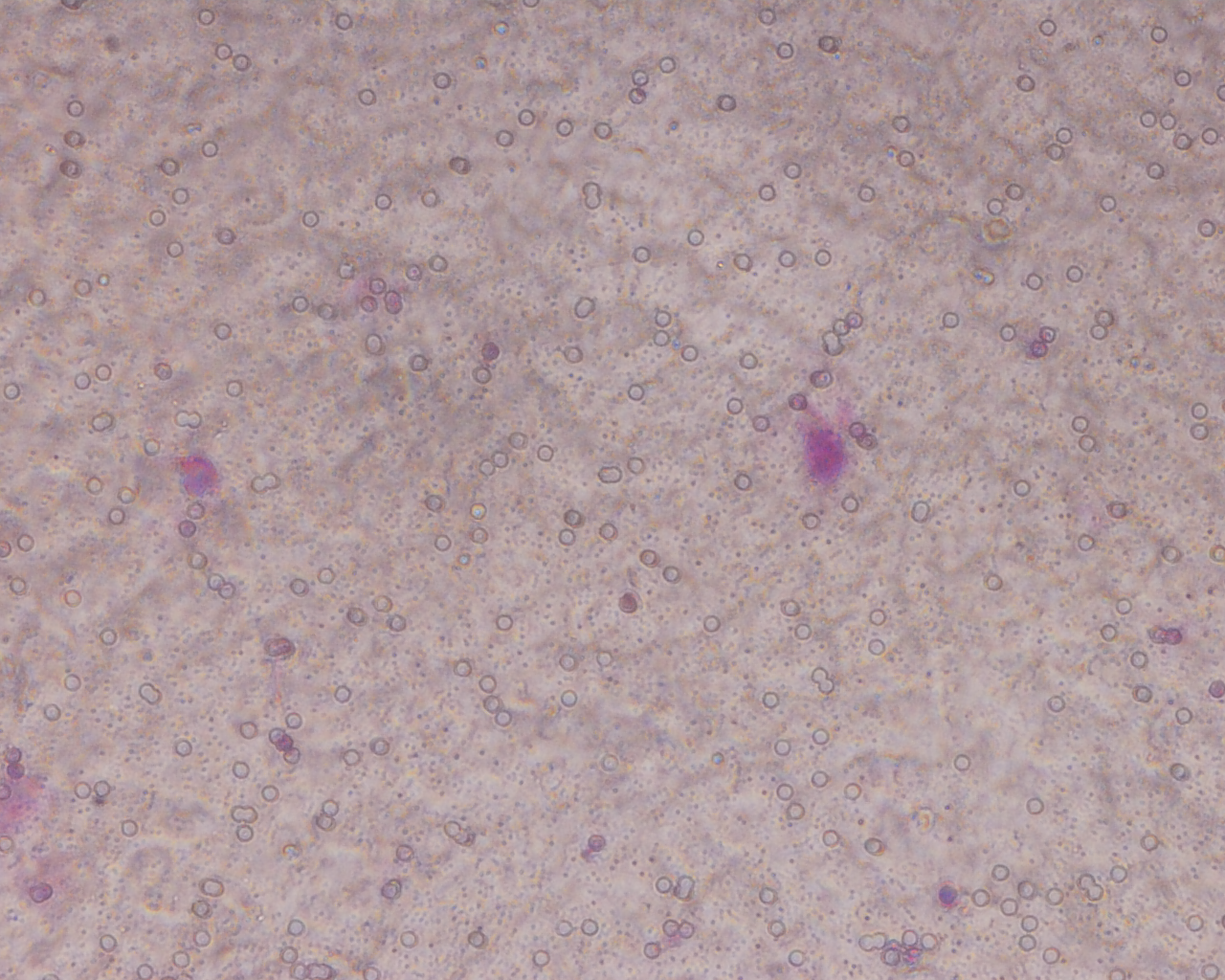

Supplement: S5 File — (ZIP) [file pgen.1010332.s005.zip › S5 File/Migration NKILA-1.tif]

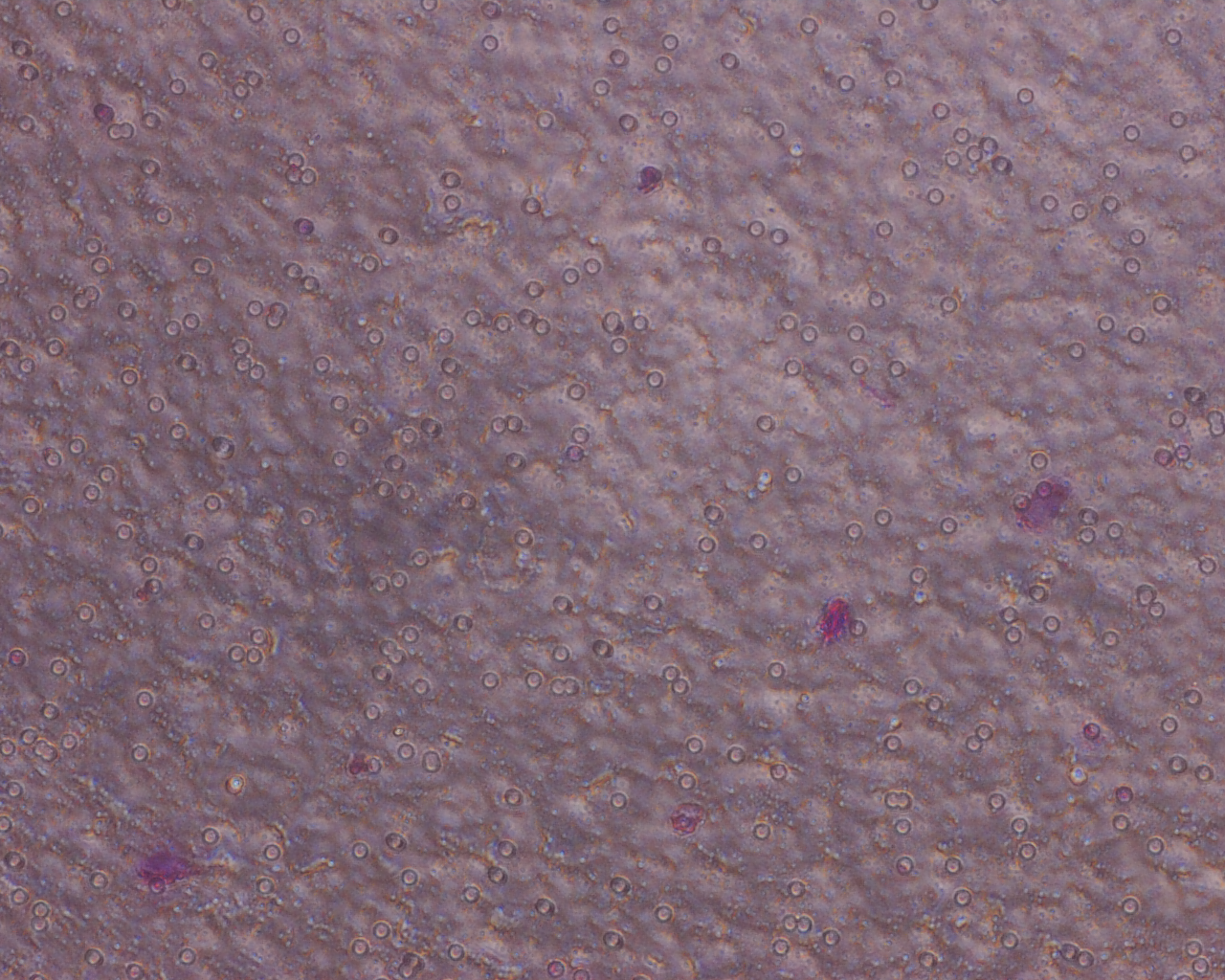

Supplement: S5 File — (ZIP) [file pgen.1010332.s005.zip › S5 File/Migration NKILA-2.tif]

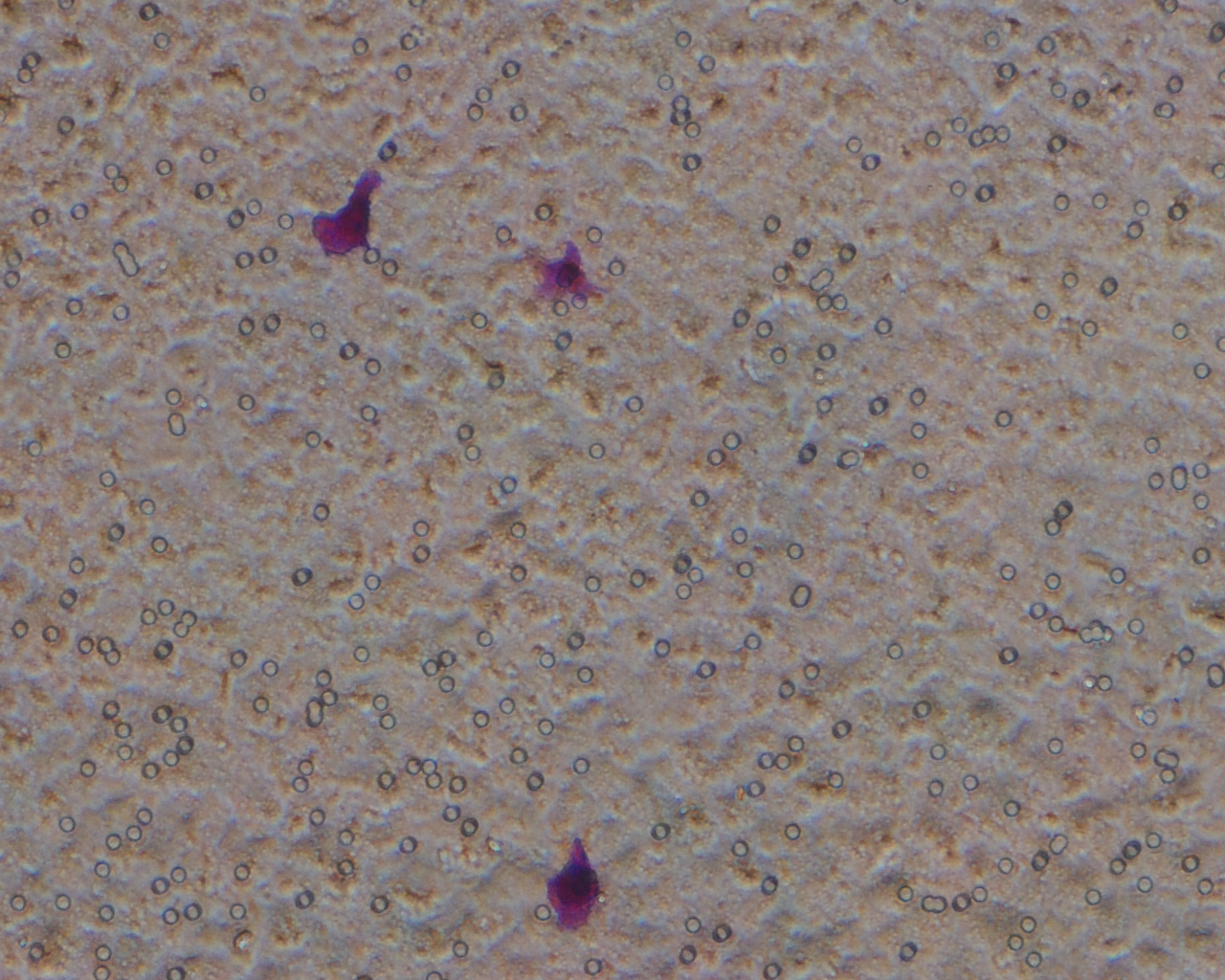

Supplement: S5 File — (ZIP) [file pgen.1010332.s005.zip › S5 File/Migration NKILA-3.tif]

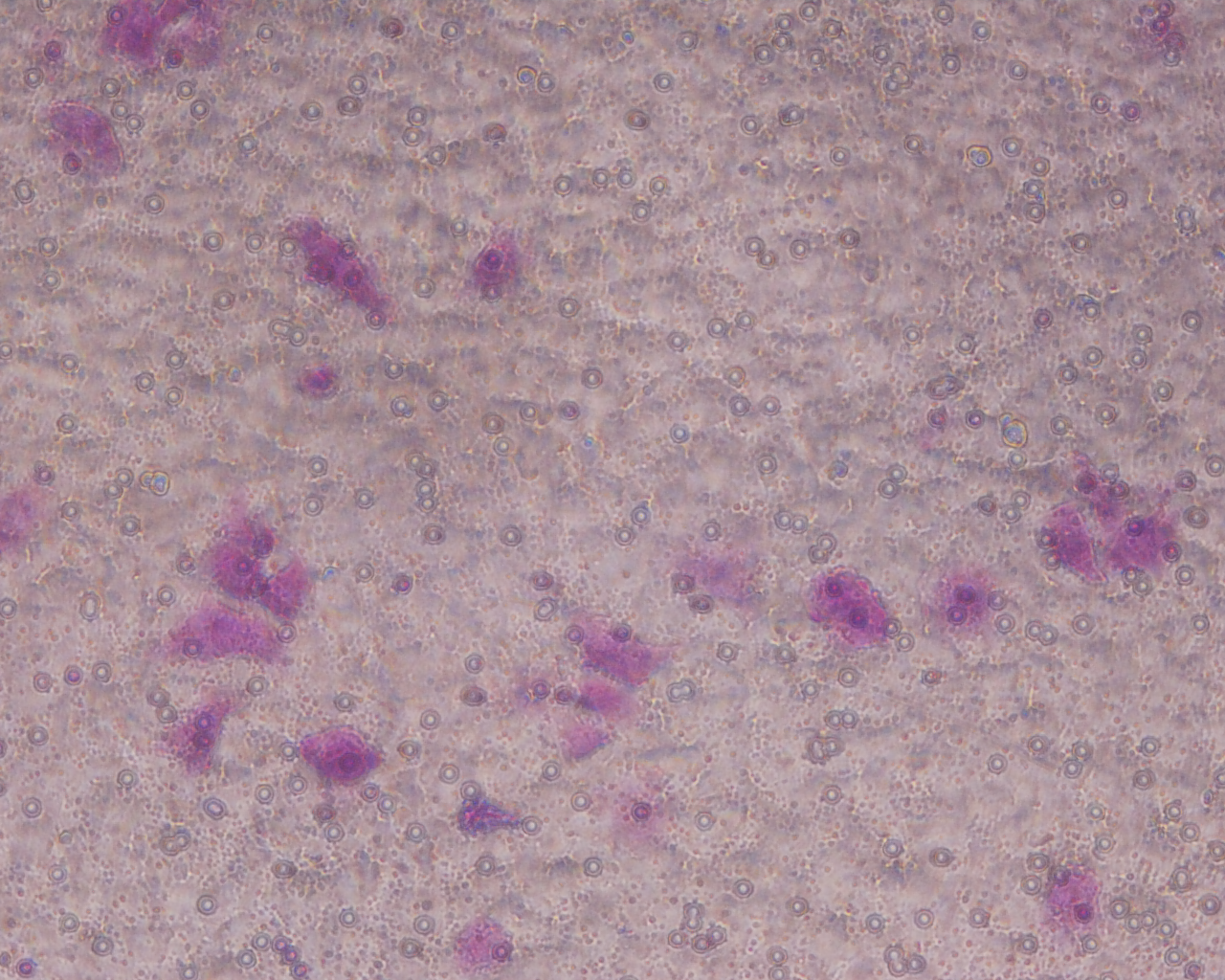

Supplement: S5 File — (ZIP) [file pgen.1010332.s005.zip › S5 File/Migration vec.tif]

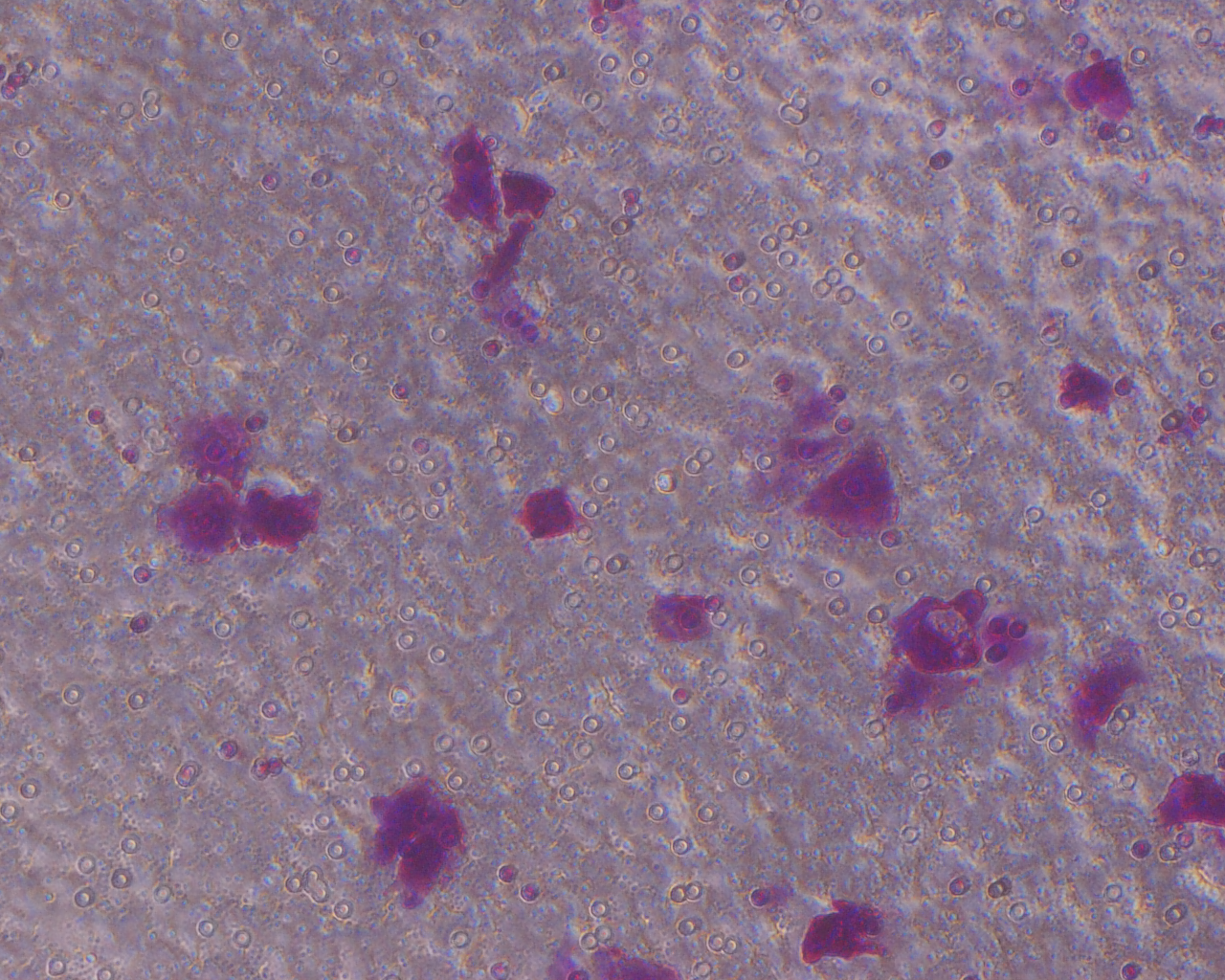

Supplement: S5 File — (ZIP) [file pgen.1010332.s005.zip › S5 File/Migration vec-1.tif]

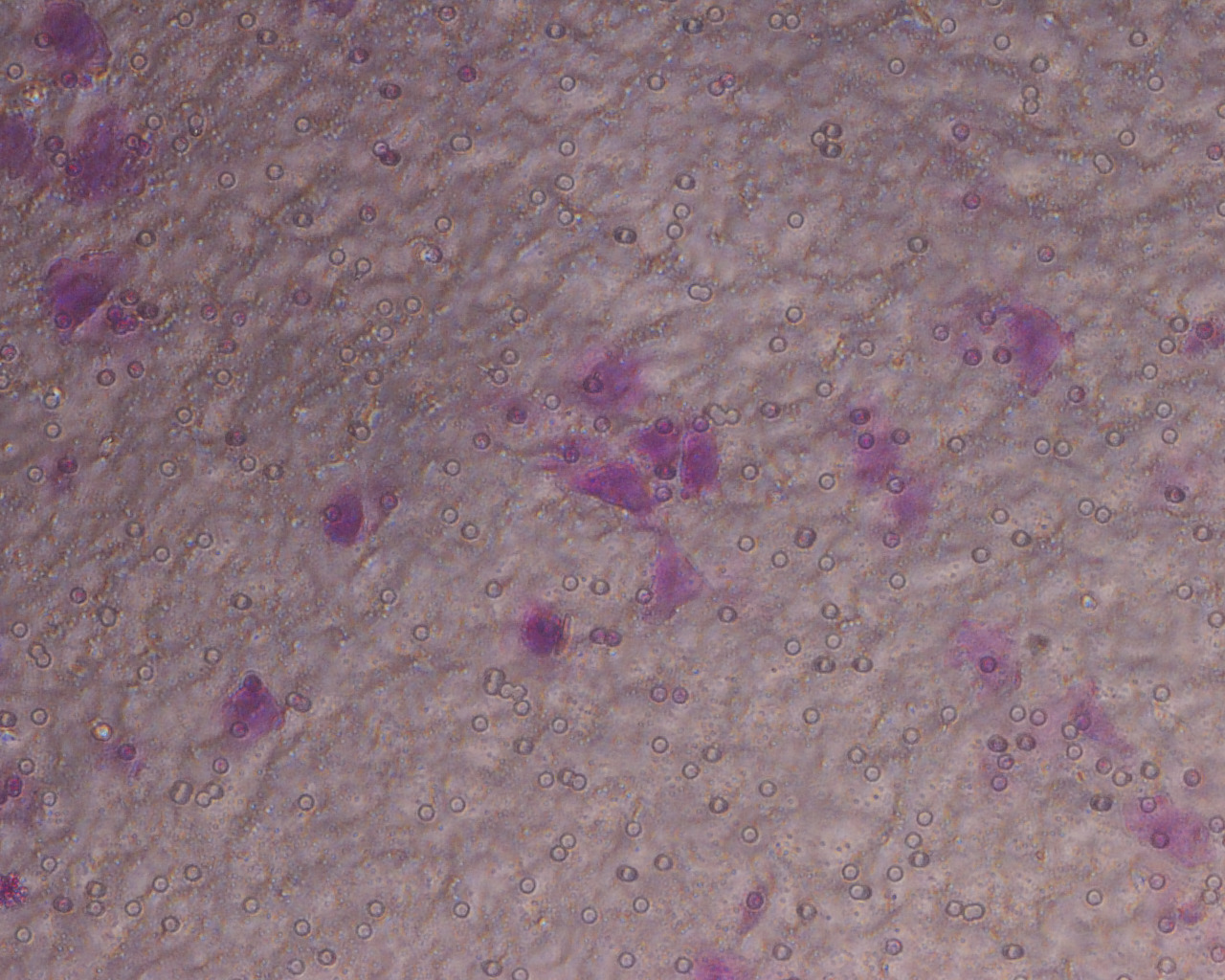

Supplement: S5 File — (ZIP) [file pgen.1010332.s005.zip › S5 File/Migration vec-2.tif]

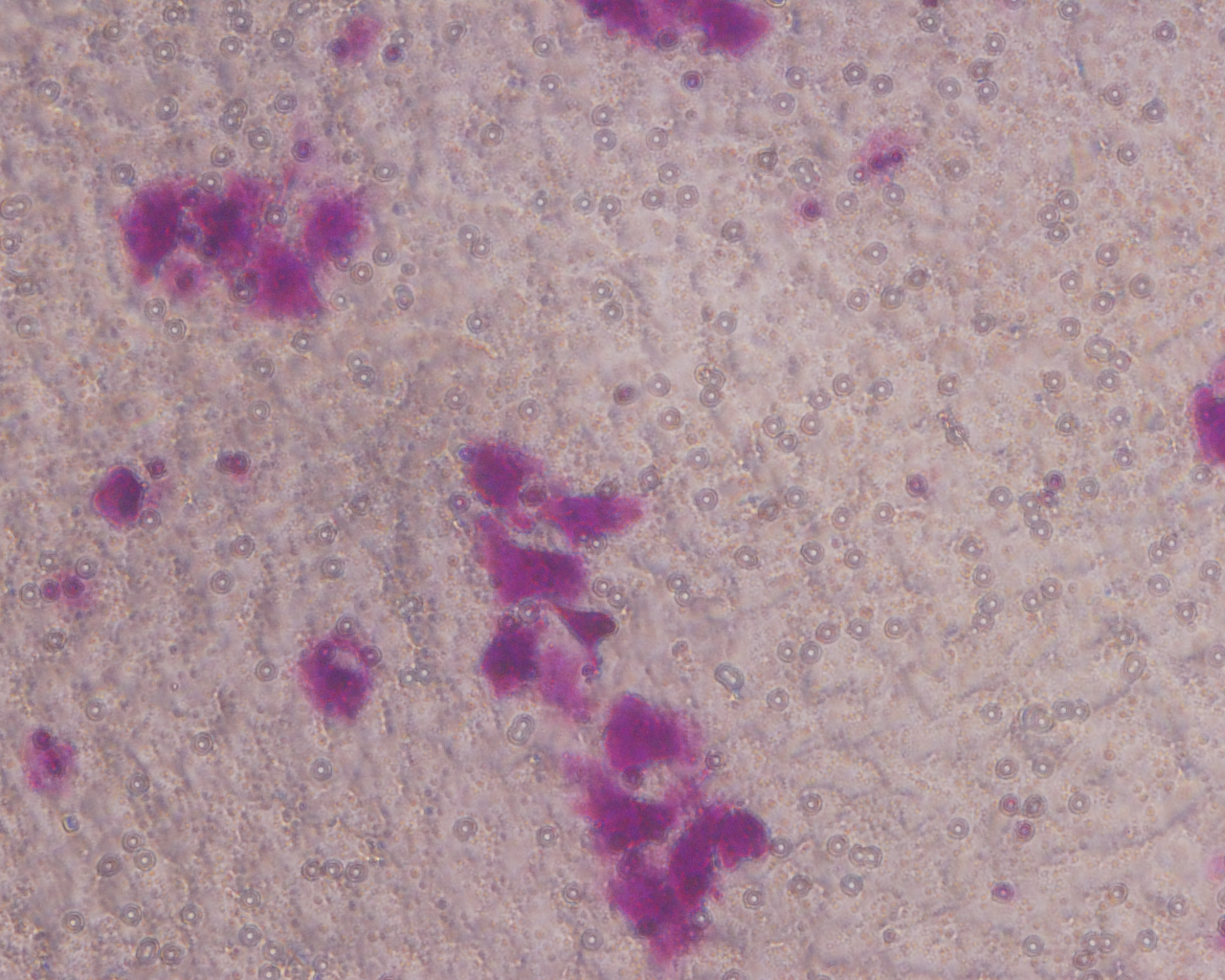

Supplement: S5 File — (ZIP) [file pgen.1010332.s005.zip › S5 File/Migration vec-3.tif]
